# Changes of signal transductivity and robustness of gene regulatory network in the carcinogenesis of leukemic subtypes via microarray sample data

**DOI:** 10.18632/oncotarget.25318

**Published:** 2018-05-04

**Authors:** Cheng-Wei Li, Tzu-Ying Lai, Bor-Sen Chen

**Affiliations:** ^1^ Laboratory of Control and Systems Biology, Department of Electrical Engineering, National Tsing Hua University, Hsinchu, Taiwan

**Keywords:** transductivity, network robustness, transductivity sensitivity, error sensitivity, basal sensitivity

## Abstract

Mutation accumulation and epigenetic alterations in genes are important for carcinogenesis. Because leukemogenesis-related signal pathways have been investigated and microarray sample data have been produced in acute myeloid leukemia (AML), myelodysplastic syndromes (MDS) and normal cells, systems analysis in coupling pathways becomes possible.

Based on system modeling and identification, we could construct the coupling pathways and their associated gene regulatory networks using microarray sample data. By applying system theory to the estimated system model in coupling pathways, we can then obtain transductivity sensitivity, basal sensitivity and error sensitivity of each protein to identify the potential impact of genetic mutations, epigenetic alterations and the coupling of other pathways from the perspective of energy, respectively. By comparing the results in AML, MDS and normal cells, we investigated the potential critical genetic mutations and epigenetic alterations that activate or repress specific cellular functions to promote MDS or AML leukemogenesis. We suggested that epigenetic modification of β-catenin and signal integration of CSLs, AP-2α, STATs, c-Jun and β-catenin could contribute to cell proliferation at AML and MDS. Epigenetic regulation of ERK and genetic mutation of p53 could lead to the repressed apoptosis, cell cycle arrest and DNA repair in leukemic cells. Genetic mutation of JAK, epigenetic regulation of ERK, and signal integration of C/EBPα could result in the promotion of MDS cell differentiation. According to the results, we proposed three drugs, decitabine, genistein, and monorden for preventing AML leukemogenesis, while three drugs, decitabine, thalidomide, and geldanamycin, for preventing MDS leukemogenesis.

## INTRODUCTION

Myelodysplastic syndromes (MDS) and acute myeloid leukemia (AML) are myeloid neoplasms. Gene expression analysis in leukemic cells including MDS and AML has revealed that hematopoietic stem cells (HSCs) are regulated for self-renewal ability, quiescence and differentiation [1]. About 10 to 30 percent of patients with MDS will progress to AML.

In oncogenesis and leukemogenesis, according to the 2-hit hypothesis mutations, genes involved in various signal transduction pathways (STPs), such as the MAPK, PI3K-AKT, NF-κB, JAK-STAT pathways, can disrupt the dynamic balance between apoptosis, cell survival, and proliferation [1–3]. Leukemic subtypes become insensitive to tumor necrosis factor (TNF)-mediated apoptotic pathways [4, 5]. The self-renewal pathways, such as transforming growth factor beta (TGFβ) superfamily, PI3 kinase, hedgehog, Notch, and Wnt signaling pathways, also dysregulate the self-renewal property of LSCs [1, 6, 7]. STPs mediate signal amplification (or attenuation) of cellular microenvironment signals to alter cellular function through transcriptional regulations. However, intrinsic variations, such as genetic mutations and epigenetic regulations, would perturb transcriptional regulations to influence cellular responses.

Although several studies have identified potential changes in PPIs [8] and genetic mutations [9, 10] during leukemogenesis, the identification of genetic and epigenetic changes from a patient group based on system theory using microarray sample data is a critical issue. In this study, we develop the transductivity of a protein in STPs and the robustness of gene regulatory networks (GRNs) based on systems theory to measure the amplification (or attenuation) ability of a protein in STPs and the ability of a GRN to tolerate intrinsic variations from the perspective of energy, respectively.

For systems analysis through microarray sample data from patients with leukemia, signal transductivity of coupling signaling pathways and information transductivity of GRNs have been proposed to infer the potential signal transduction change between attenuation and amplification from normal type to AML subtype in each node of coupling signaling pathways [11]. However, epigenetic regulations, such as acetylation, lysine and arginine methylation, phosphorylation, ubiquitination and sumoylation, and the impact of pathways other than the well-characterized STPs would play an important role in leukemogenesis [12, 13]. Therefore, in this study we define the transductivity sensitivity, basal sensitivity, and error sensitivity of each protein in STPs to suggest the dysregulated proteins in STPs leading to AML/MDS leukemogenesis and the potential leukemogenesis from MDS to AML from the perspective of energy transportation based on genetic and epigenetic changes.

Because large amounts of microarray sample data from patients with leukemia are available, system modeling and identification are applied to leukemogenesis-related STPs to identify the genetic and epigenetic changes through the defined transductivity sensitivity, basal sensitivity, and error sensitivity of each protein during leukemogenesis. The transductivity sensitivity, basal sensitivity, and error sensitivity are used to suggest the potential impact of signal transduction change, the epigenetic alteration and the coupling of other pathways leading to leukemogenesis, respectively. According to the transductivity sensitivities in AML and MDS, we proposed potential drugs for treating the patients with AML or MDS using drug response genome-wide microarray data.

## RESULTS

### More network robustness of GRN in seven leukemic subtypes than in normal type

**Figure 1:**
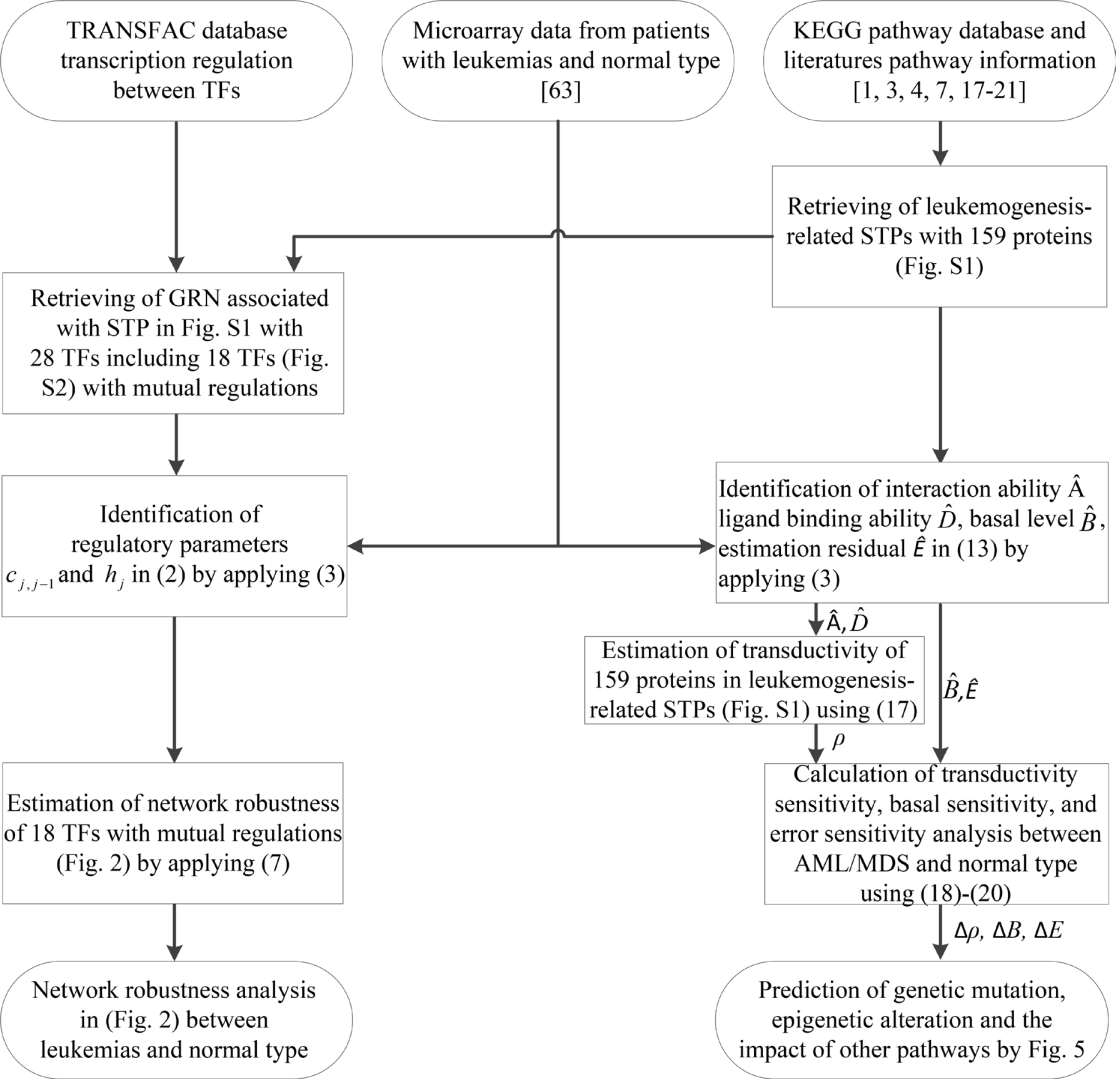
Flowchart of the proposed method for estimating network robustness of the GRNs and for predicting the impact of genetic mutations, epigenetic alterations and the coupling of other pathways of the proteins in the coupling STPs (Supplementary Figure 1)

**Figure 2:**
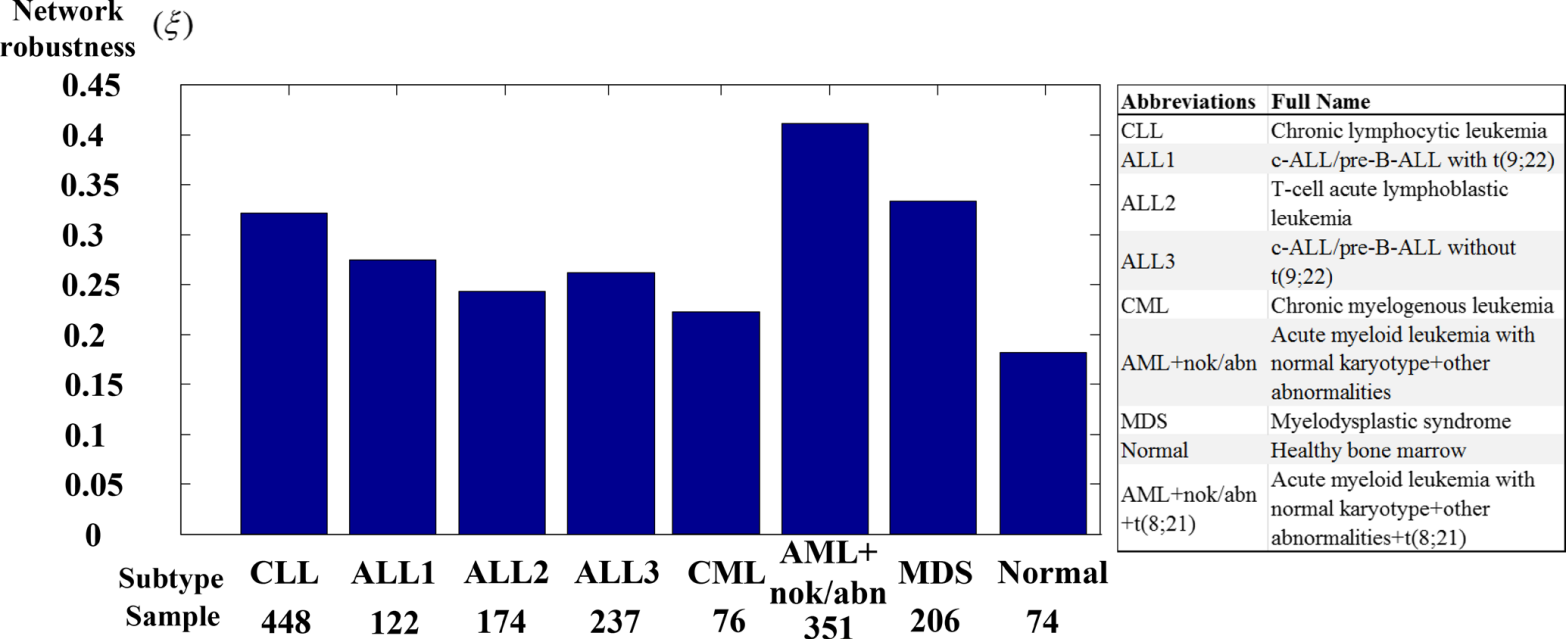
The network robustness of Supplementary Figure 2 across seven leukemic subtypes and one normal type The normal type is with the smallest robustness (0.18) by comparison to other leukemic subtypes. It means that normal type is less tolerable to intrinsic perturbation such as genetic mutation or epigenetic alteration and more responsible to external molecular signals than leukemic subtypes. Among these leukemic subtypes, the GRNs of AML+nok/abn and MDS with higher robustness than other leukemic subtypes can give harbor to a diversity of intrinsic genetic mutation and epigenetic alteration and further develop a variety of evolutionary process to leukemogenesis than other leukemic subtypes.

A flowchart of the method for estimating the transductivity sensitivity, basal sensitivity, and error sensitivity of each protein in STPs and the network robustness in GRNs is presented in Figure 1. In nucleus of the leukemogenesis-related STPs (Supplementary Figure 1; also extracted in Supplementary Figure 2 in Supplementary Materials), we measure network robustness of the GRN across 7 leukemic subtypes and the normal type using microarray sample data based on system identification in (3) and system theory in (7). In Figure 2, the result shows that the normal type causes the smallest robustness and the AML (or AML with normal karyotype plus other abnormalities) and MDS subtypes cause higher robustness than the others. For the intrinsic variations, including the molecular alteration and genetic and epigenetic changes, in the nucleus, we suggested that the GRNs in the patients with AML and MDS would harbor more variations to drive the diversity and progression of leukemic cells during leukemogenesis. Furthermore, we measure the transductivity sensitivities of the 28 TFs in the STPs of the patients with AML, MDS and normal type to evaluate the potential signal transduction changes during leukemogenesis.

### Transductivity sensitivities of downstream 28 TFs in the coupling STPs of AML, MDS and normal type

**Figure 3:**
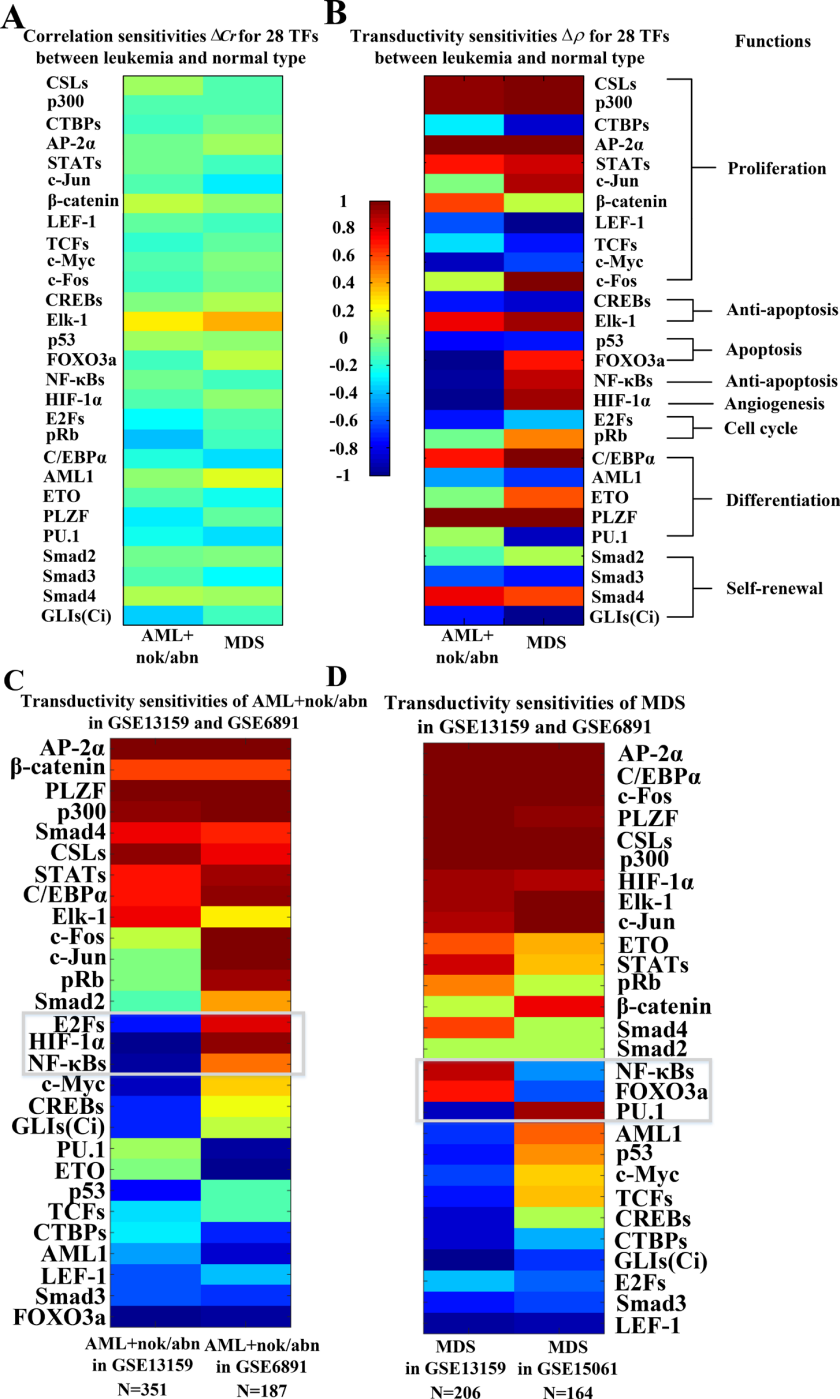
Comparison of correlation sensitivities (**A**) and transductivity sensitivities (**B**) for 28 TFs between AML+nok/abn and MDS in the same dataset (GSE6891). Comparison of transductivity sensitivities for 28 TFs at AML+nok/abn (**C**) or at MDS (**D**) between two datasets, i.e., between GSE13159 and GSE6891 at AML+nok/abn or between GSE13159 and GSE15061 at MDS. According to top 3 TFs with the largest differences between two datasets at AML+nok/abn (C) and at MDS (D) including FOXO3a, NF-κBs, HIF-1α, E2Fs, and PU.1, the TFs also have large differences between AML+nok/abn and MDS in (B). Therefore, we inferred that the 5 TFs with the largest differences in transductivity sensitivity result from the effect of individual difference on the induction of redundant cellular functions to promote AML/MDS leukemogenesis. We suggested that the proposed results (B) to compare the transductivity sensitivities between AML+nok/abn and MDS in the same dataset (GSE13159) are reliable.

The transductivity sensitivities Δ*ρ*, which mean the changes of transductivity between leukemic subtypes and normal type (see Materials and Methods), and the corresponding transductivities *ρ* of 28 TFs at AML and MDS cells are all shown in Supplementary Figure 3. The overall transductivity sensitivities Δ*ρ* of 28 TFs at seven leukemic subtypes and one normal type can refer to Figure 3B. In Figure 3B, we sort rows based on cellular functions. The color bars are associated with transductivity sensitivities from -1 (blue) to +1 (red). A TF in red color denotes its high signal transduction ability in leukemic subtype comparing to normal type, while a TF in blue color denotes its high transduction ability in normal type comparing to leukemic subtype. The aberrant signal transduction ability of a TF in leukemic subtype could be due to the accumulated genetic and epigenetic changes in the STPs.

In Figure 3B, the result shows that six proliferation-related TFs, CSLs, p300, STATs, c-Jun, β-catenin, and AP-2α, and the differentiation-related TF C/EBPα lose more functions at AML and MDS subtypes than normal type. Two proliferation-related TFs, c-Jun and c-Fos, and the apoptosis-related TF FOXO3a lose more functions at normal type and AML subtype than MDS subtype. The results can be supported by the fact that AML is characterized by differentiation and apoptosis blocking while MDS is characterized by impaired differentiation and apoptosis.

## DISCUSSION

### Increased network robustness of GRN from normal type to AML in leukemogenic process

In the result (Figure 2), we calculated the network robustness of the GRN with the downstream 28 TFs in the coupling STPs (Supplementary Figure 1). It shows that the GRN in AML cause the highest network robustness. It implicates that AML causes the reduced response of GRN to cellular signals and high tolerance to genetic and epigenetic changes. Clinically diagnosed AML patients have genetic diversity, which lead to difficult treatment interventions. The incidence of AML increased with age [14, 15]. Therefore, the observation can support our result in network robustness. Furthermore, we calculated transductivity sensitivity, basal sensitivity, and error sensitivity of each protein, including 28 TFs, in the well-characterized STPs (Supplementary Figure 1) to suggest the potential impact of signal transduction change, the epigenetic alteration and the coupling of other pathways in leukemogenic process. We select seven proliferation-related TFs (CSLs, p300, CTBPs, AP-2α, STATs, c-Jun and β-catenin), two apoptosis-related TFs (p53, FOXO3a) and three differentiation-related TFs (C/EBPα, AML1, and ETO), the critical TFs to integrate signals from multiple pathways to transcriptionally regulate target genes in Supplementary Figure 1, to discuss their gain or lose cellular functions in leukemia using transductivity sensitivity, basal sensitivity, and error sensitivity.

### Epigenetic modification of β-catenin and signal integration of CSLs, AP-2α, STATs, c-Jun and β-catenin contributing to cell proliferation at AML and MDS

**Figure 4:**
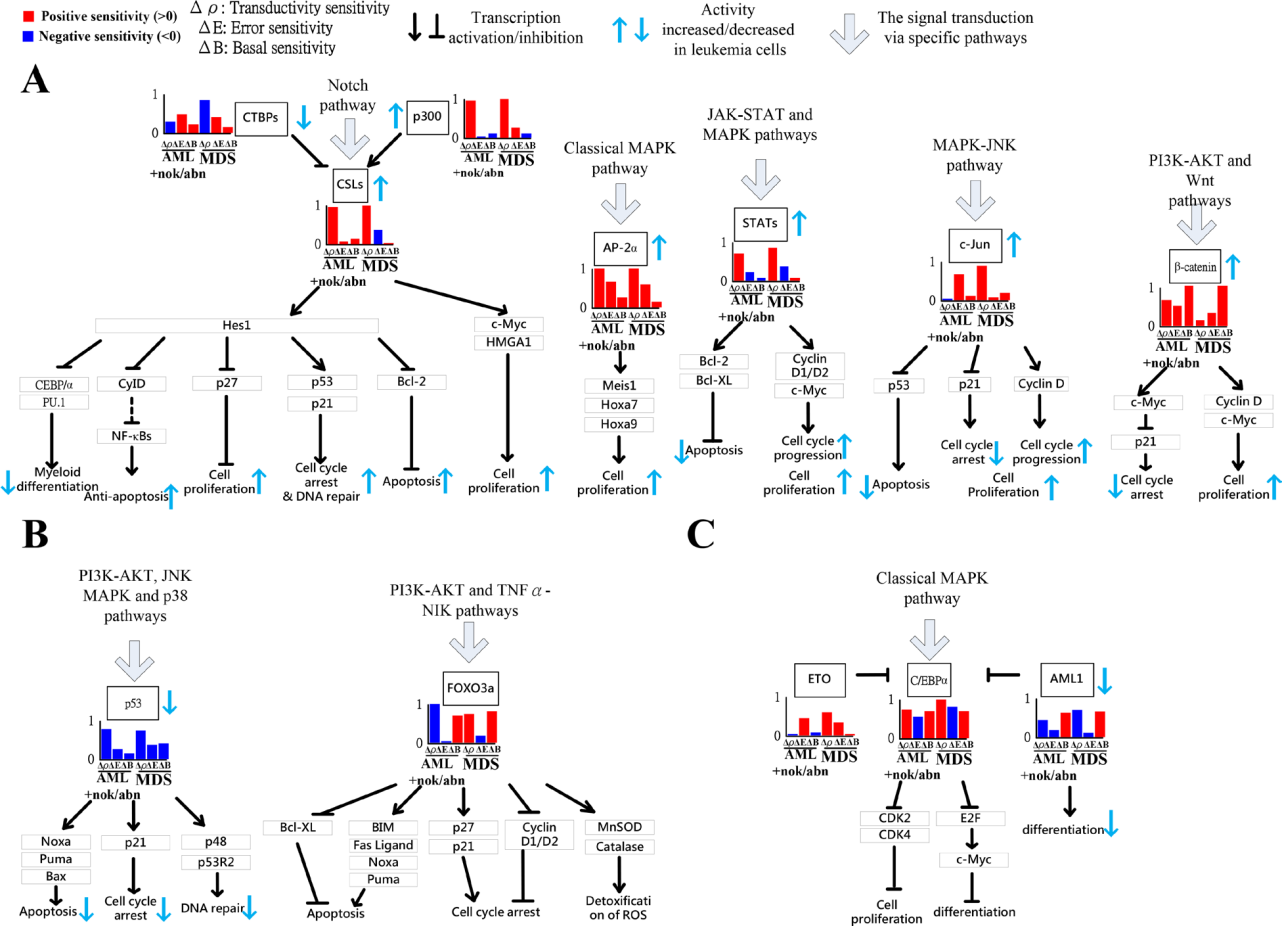
Gain or lose of cellular functions of five proliferation-related TFs (CSLs, AP-2α, STATs, c-Jun and β-catenin) (**A**), two apoptosis-related TFs (p53, FOXO3a) (**B**) and three differentiation-related TFs (C/EBPα, AML1, and ETO) (**C**) in AML/MDS leukemogenesis. If the transductivity sensitivity of a TF, such as CSLs in (A), in a leukemic subtype was positive, Δρ > 0 (Red bar), its activity was increased in the leukemic subtype when comparing to normal type. In contrast, the activity of p53 in (B) was decreased at leukemic subtypes. The large error sensitivity of a TF in a leukemic subtype, such as AP-2α in (A), and C/EBPα in (C) in both AML+nok/abn, and MDS, and c-Jun in (A) in AML+nok/abn, represents the probable impact of pathways other than those in Supplementary Figure 1. Additionally, the large basal sensitivity of a TF in a leukemic subtype, such as AML1, C/EBPα, β-catenin, and FOXO3a in (A–C) in both AML+nok/abn and MDS, implicates the probable impact of epigenetic regulation on the TF-coding gene.

As shown in Figures 3B and 4A, five proliferation-related TFs (CSLs, AP-2α, STATs, c-Jun and β-catenin) integrate signals from the Notch pathway, the classical MAPK pathway, the JAK-STAT and MAPK coupling pathways, the MAPK-JNK pathway, and the PI3K-AKT and Wnt coupling pathways to activate cell proliferation by regulating target genes, respectively. The increased transductivity of p300 and the decreased transductivity of CTBPs at both AML and MDS than normal type activate and repress CSLs to promote its downstream transcription regulations, respectively. It has been reported that, within the nucleus, interaction of CSLs with the intracellular part of the Notch (ICN) leads to the activated transcription of Hairy and Enhancer of Split 1 (HES1) and High-mobility group AT-HOOK 1 (HMGA1) through Bcl-2, C/EBPα, p21, *SPI1* (encoding PU.1) and p53 to induce cell proliferation during leukemogenesis [16, 17].

Additionally, the positive transductivity sensitivities of AP-2α, STATs, c-Jun, and β-catenin at both AML and MDS transduce more signals to cellular functions by activating or repressing their downstream genes. It leads to that AP-2α and c-Jun gain more functions of cell proliferation and c-Jun gains more functions of prevention from cell death at AML and MDS. The result in the transductivity sensitivity analyses of AP-2α and c-Jun is consistent with the dysfunctions which contribute to leukemogenesis [18, 19]. Since AP-2α has high error sensitivity and also plays an important role in the development of hepatocellular carcinoma, nasopharyngeal carcinoma, gastric adenocarcinoma, prostate cancer, pancreatic cancer and breast cancer, the dysregulated AP-2α would be attributed to pathways other than the leukemogenesis-related coupling STPs (Supplementary Figure 1). The activation of STATs target genes c-Myc, cyclin D, Bcl-XL and Bcl-2 dysregulates cellular function, which would lead to cellular escape from apoptosis and cell proliferation during leukemogenesis [15, 20, 21]. The experimental observations support our result in Figure 3B.

In Figure 4B, the basal sensitivity of β-catenin is high at AML and MDS. We suggested that the epigenetic changes of β-catenin results in the up-regulated cell proliferation and the down-regulated cell cycle arrest. It has also been observed that the epigenetic modification of β-catenin could promote cell proliferation and lead to escape from quiescence of HSC during leukemogenesis [1, 22].

### Epigenetic modification of FOXO3a contributing to the repressed apoptosis at AML

As shown in Figures 3B and 4B, two apoptosis-related TFs (p53, FOXO3a) integrate signals from the PI3K-AKT, JNK MAPK and p38 coupling pathways and PI3K-AKT and TNFα-NIK coupling pathways to repress apoptosis in AML subtype. The down-regulated apoptosis, DNA repair and cell cycle arrest in AML tumorigenesis through the dysregulated p53 can also be supported [23–25].

In addition, the result in Figure 4B shows that the forkhead protein FOXO3a (forkhead transcription factor O subfamily member 3a) has negative transductivity sensitivity and high basal sensitivity in AML subtype. We suggested that the epigenetic change of FOXO3a causes the repressed apoptosis in AML tumorigenesis. The methylation of FOXO3a has been also reported to dysregulate cellular function in AML subtype [26, 27].

### Epigenetic modification of C/EBPα and AML1 and signal integration of C/EBPα from various pathways contributing to cellular differentiation at AML and MDS

As shown in Figures 3B and 4C, three differentiation-related TFs (C/EBPα, AML1, and ETO) integrate signals from the classical MAPK pathway to repress apoptosis. The result in Figure 4C shows that ETO has positive transductivity sensitivity in MDS subtype, and C/EBPα and AML1 have high basal sensitivities in AML and MDS subtypes. We suggested that the epigenetic changes of C/EBPα and AML1 participate in AML and MDS leukemogenesis, and ETO only transduces more signals at MDS, which leads to MDS cells to retain their ability to differentiate. The epigenetic modifications of C/EBPα [28] and AML1 [13, 29] at AML and MDS, which participate in AML and MDS leukemogenesis, and the intact differentiation ability of MDS [30] are also supported. Other pathways such as the endoplasmic reticulum (ER) stress pathway, not involved in the 8 leukemogenesis-related coupling STPs (Supplementary Figure 1), have been also suggested to participate in the induction of transcriptional regulation by C/EBPα in AML and MDS leukemogenesis [31, 32].

### Prediction of the impact of the genetic mutation, the epigenetic regulation, or the coupling of other pathways in the leukemogenesis-related coupling STPs at AML and MDS

**Figure 5:**
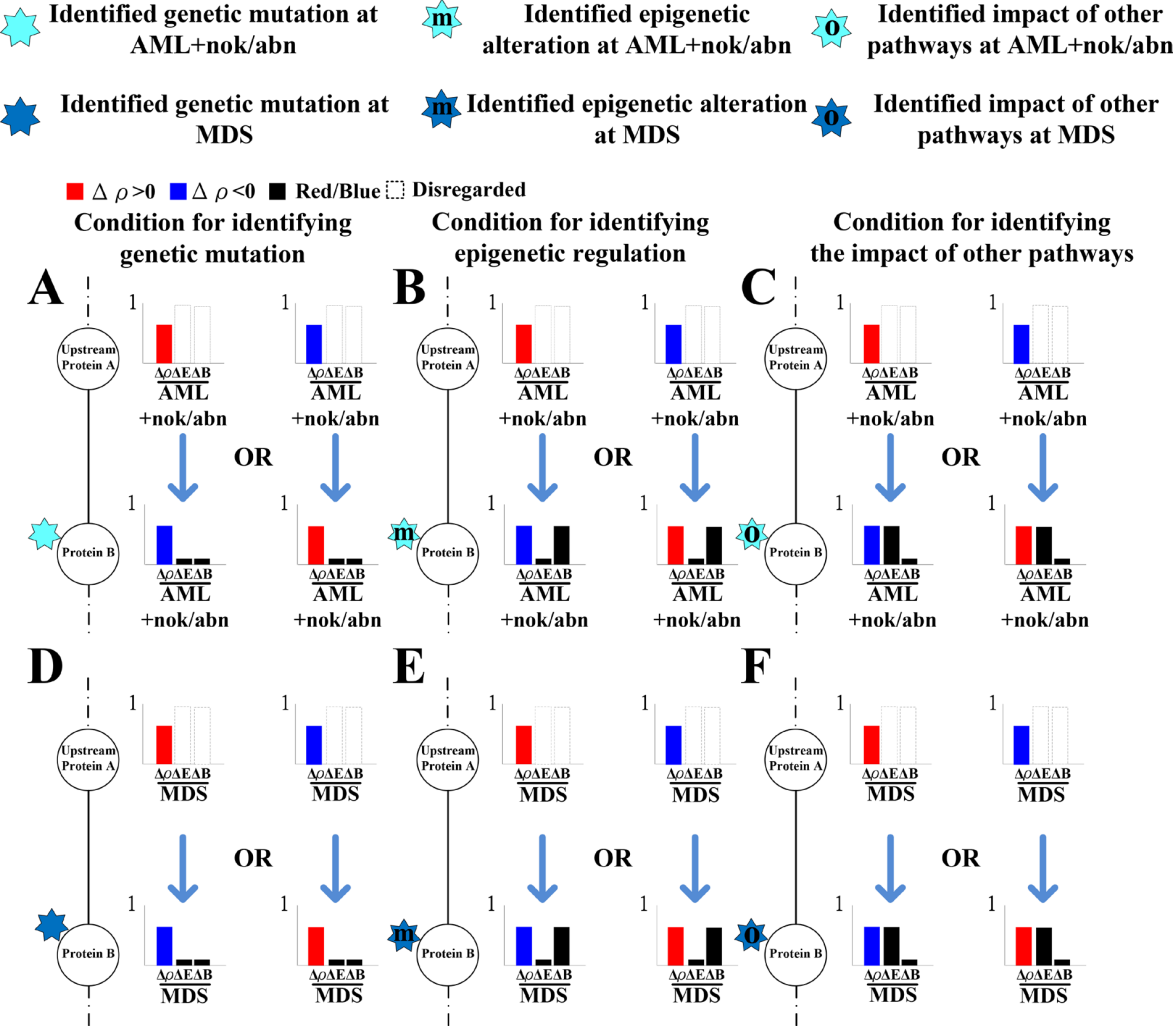
The illustration for identifying the impact of genetic mutation, epigenetic regulation and the coupling of other pathways on the downstream protein The sign change of the transductivity sensitivities between two proteins implicated the impact of genetic mutation, epigenetic regulation or the coupling of other pathways on the downstream protein B. The black bar denotes the absolute value of the error sensitivity ΔE or basal sensitivity ΔB. The absolute values of the basal and error sensitivities in protein B were higher than 0.5 indicated epigenetic regulation ((**B**) at AML and (**E**) at MDS) and the impact of other pathways ((**C**) at AML and (**F**) at MDS) on B, respectively. Otherwise, genetic mutation occurred in protein B ((**A**) at AML and (**D**) at MDS).

**Figure 6:**
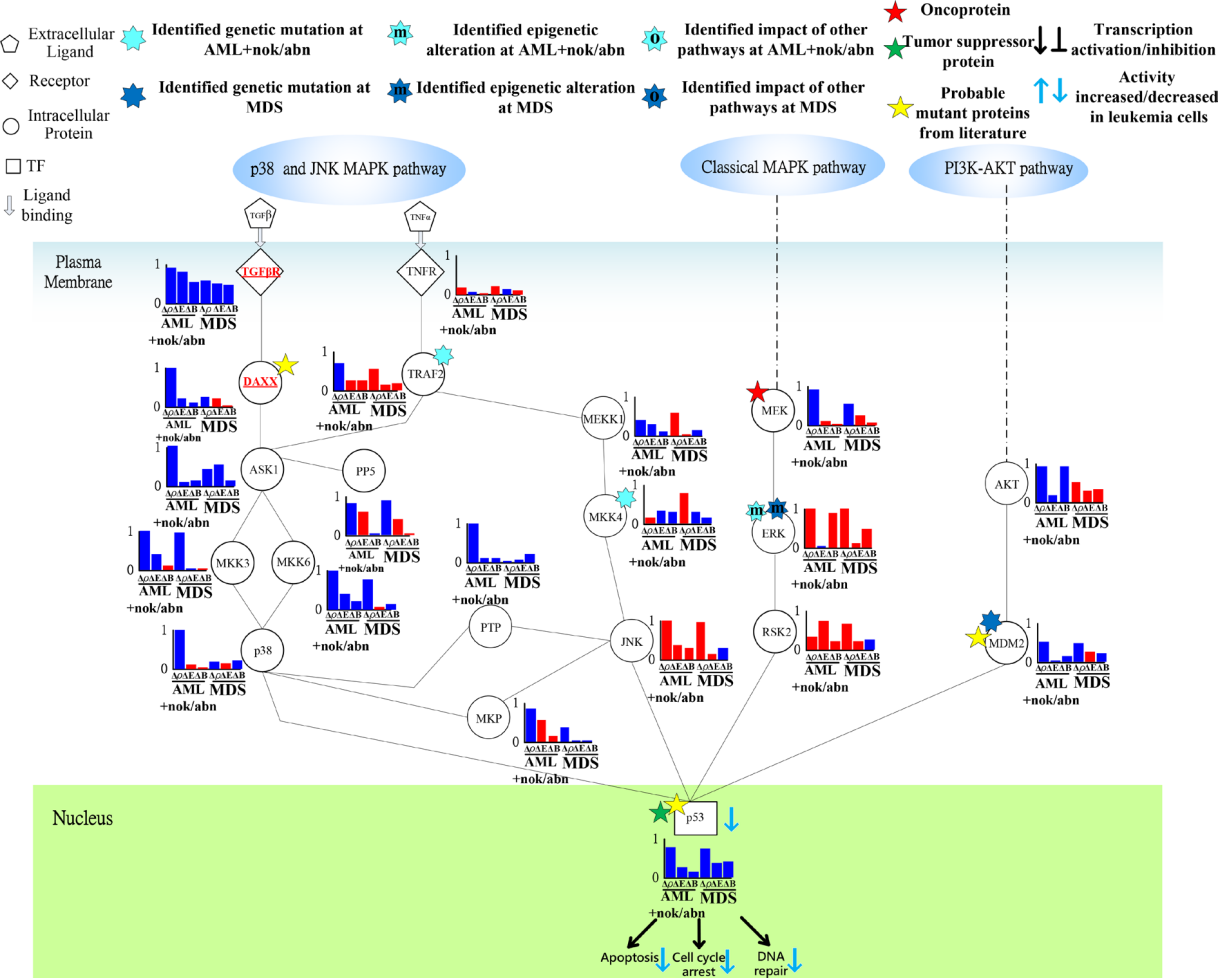
Transductivity sensitivity, error sensitivity, and basal sensitivity of the coupled pathways contributing to the loss of transductivity of p53 at AML+nok/abn and MDS cells when comparing to the normal type The identified genetic mutations on the genes TRAF2, and MKK4 at AML+nok/abn cell, and MDM2 at MDS cell were by the sign change of the transductivity sensitivity at leukemic subtype when comparing to the upstream protein. The identified effect of epigenetic regulation on the gene ERK at both leukemic subtypes was by not only the sign change of the transductivity sensitivity at leukemic subtype, but also the large basal sensitivity of ERK at both leukemic subtypes. The proteins shown in red symbols with underline in the STPs are analyzed as the main causes of dysfunction of TF.

**Figure 7:**
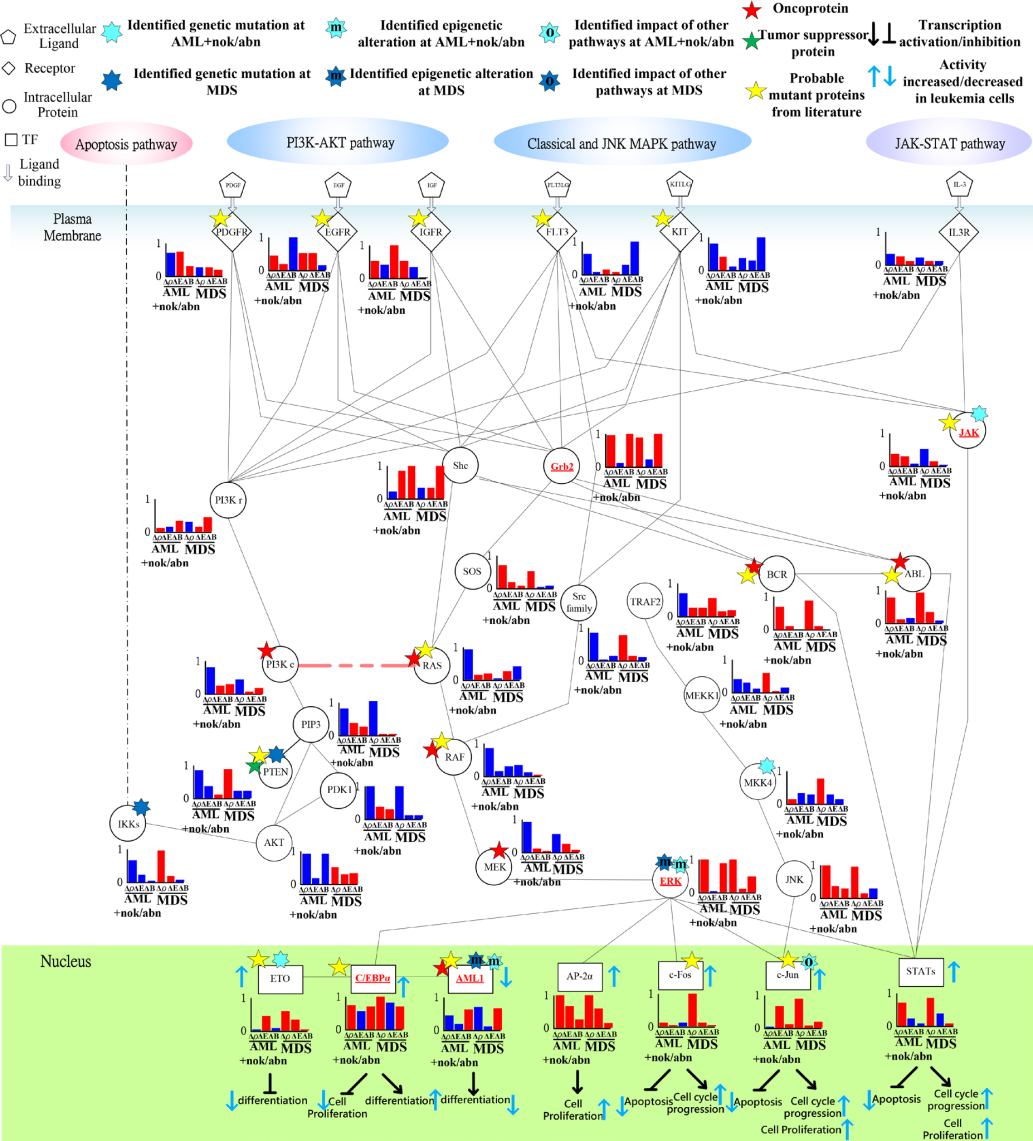
Transductivity sensitivity, error sensitivity, and basal sensitivity of the coupled pathways contributing to gain of transductivity of STATs and C/EBPα at AML+nok/abn and MDS cells when comparing to the normal type The identified genetic mutations on the genes JAK, MKK4, and ETO at AML+nok/abn cell, and PTEN, and IKKs at MDS cell were due to the sign change of the transductivity sensitivity at leukemic subtype when comparing to the upstream protein. The identified effect of epigenetic regulation on the gene ERK, and AML1 at both leukemic subtypes was due to not only the sign change of the transductivity sensitivity at leukemic subtype, but also the large basal sensitivities of ERK, and AML1 at both leukemic subtypes. The identified impact of other pathways on the gene c-Jun at AML+nok/abn cell was due to not only the sign change of the transductivity sensitivity at leukemic subtype, but also the large error sensitivity of c-Jun at AML+nok/abn cell.

**Figure 8:**
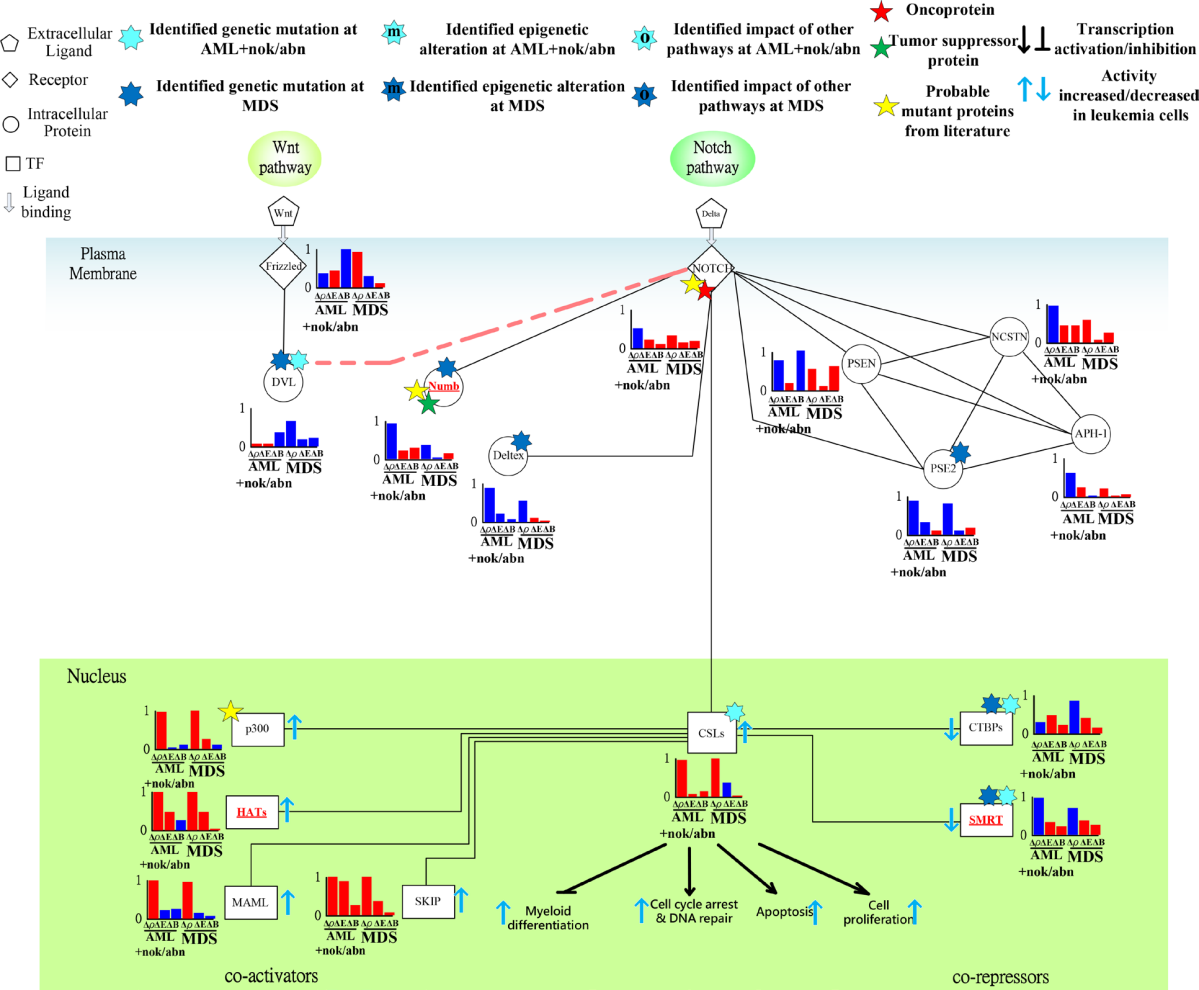
Transductivity sensitivity, error sensitivity, and basal sensitivity of the coupled pathways contributing to the gain of transductivity of CSLs at AML+nok/abn and MDS cells when comparing to the normal type The identified genetic mutations on the genes CSLs at AML+nok/abn cell, Numb, Deltex, and PSE2 at MDS cell, and DVL, CTBPs, and SMRT at both cells were by the sign change of the transductivity sensitivity at leukemic subtype when comparing to the upstream protein.

**Figure 9:**
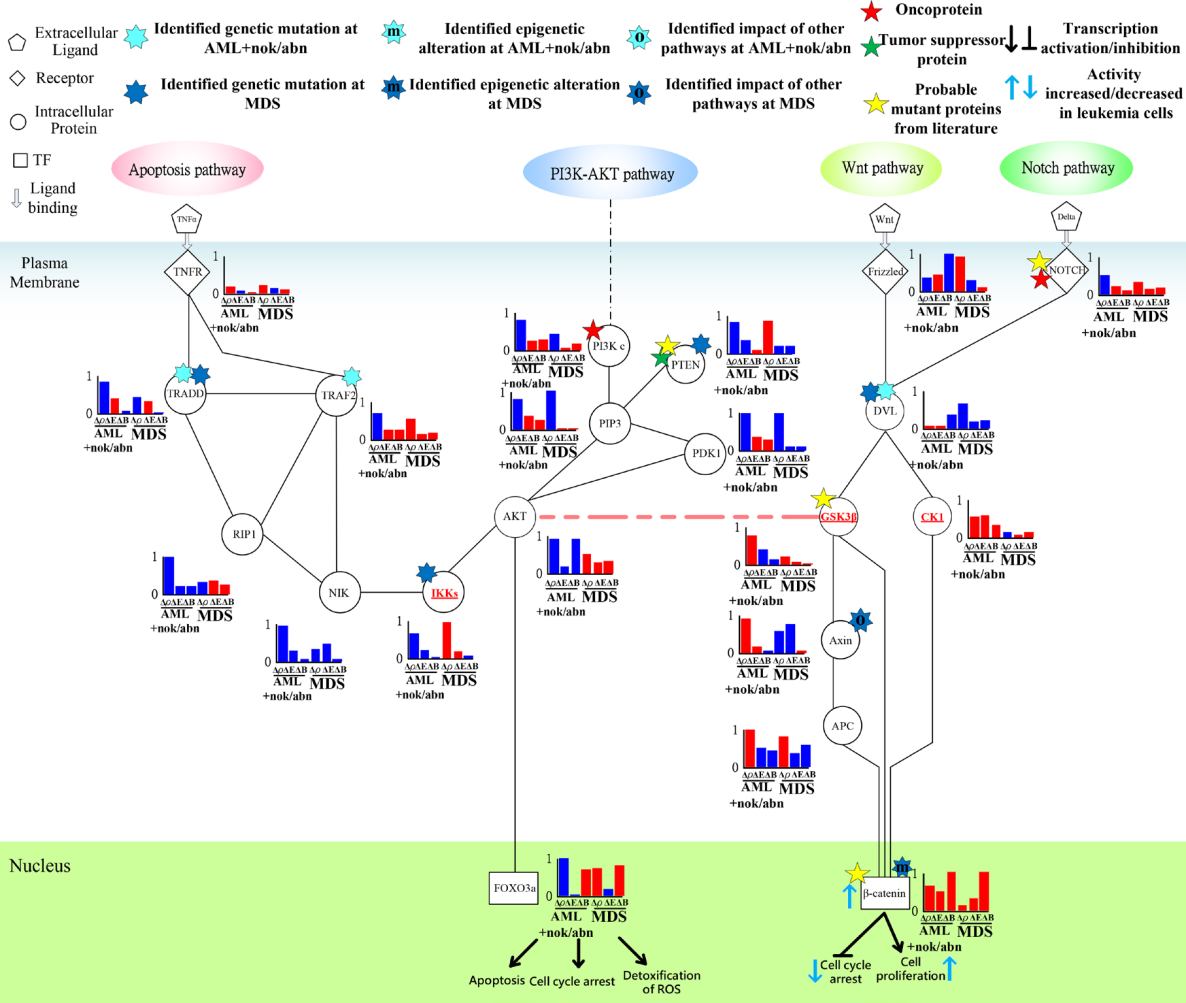
Transductivity sensitivity, error sensitivity, and basal sensitivity of the coupled pathways associated with β-catenin and FOXO3a at AML+nok/abn and MDS cells when comparing to the normal type The gain of function in β-catenin attributed to the genetic mutations in DVL and the interaction site between AKT and GSK3β in AML and the genetic mutations in DVA and the impact of other pathways on Axin in MDS. The loss of function in FOXO3a in AML was due to the genetic mutations in TRADD and TRAF2.

To identify the impact of genetic mutation, the epigenetic regulation, or the coupling of pathways other than those in Supplementary Figure 1 from the perspective of signal transduction, the following definitions are provided based on the transductivity sensitivity, basal sensitivity, and error sensitivity of each protein. The genetic mutation of a protein in a leukemic subtype was predicted by the sign change of the transductivity sensitivities between the protein and its upstream one in this cell, and its lower absolute values of the error and basal sensitivities (<0.5) (Figure 5A and 5D). The epigenetic regulation of a protein in a leukemic subtype was predicted by not only its higher absolute values of the basal sensitivity (>0.5) but also the sign change of the transductivity sensitivities between the protein and its upstream one in the cell (Figure 5B and 5E). The impact of other pathways except those in Supplementary Figure 1 was predicted by not only its higher absolute values of the error sensitivity (>0.5), but also the sign change of the transductivity sensitivities between the protein and its upstream one in this cell (Figure 5C and 5F). Figures 6–9 reveal the predicted impact of genetic mutations, epigenetic modifications, and the coupling of other pathways in genes encoding proteins in the leukemogenesis-related coupling STPs (Supplementary Figure 1) at AML and MDS (indicated 7-pointed stars).

### Epigenetic regulation of ERK and genetic mutation of p53 leading to the repressed apoptosis, cell cycle arrest and DNA repair

In Figure 6, the result shows sign change of transductivity sensitivities between TNFR and TRAF2 from positive to negative in AML subtype. We suggest that the identified genetic mutation in TRAF2 contributs to the down-regulated apoptosis, DNA repair and cell cycle arrest in AML subtype.

Additionally, the sign change of transductivity sensitivity between MEKK1 and MKK4 denotes the identified mutation in MKK4 in AML subtype (Figure 6). The sign changes of transductivity sensitivity between PTP and JNK, and between MKP and JNK indicated that genetic mutations could occur in these two interaction sites of JNK in AML cells (Figure 6). Furthermore, the sign changes of transductivity sensitivity between JNK and p53, and between RSK2 and p53 indicated that genetic mutations could occur in these two interaction sites of p53 in AML cells (Figure 6). The identified mutation in p53 could cause the repressed apoptosis in AML and MDS subtypes.

In Figures 6 and 7, the result in the classical MAPK pathway shows that RAS, RAF MEK and p53 have negative transductivity sensitivities. The sign change of transductivity sensitivity was observed between MEK and ERK, and the downstream protein ERK showed high basal sensitivity (Figure 6), which have been thought to be influenced by DNA methylation in AML and MDS cells [33]. Although epigenetic regulation of ERK could transduce enhanced signals to induce apoptosis, the mutated p53 in the downstream of the classical MAPK pathway repressed the apoptosis, cell cycle arrest and DNA repair.

In contrast, the transductivity sensitivities of proteins in the p38 pathways, except TNFR and TRAF2, were negative in MDS cells (Figure 6). Unlike AML cells, the genetic mutations of TRAF2, and MKK4 were not found in MDS cells. JNK in MDS cells still exhibits the same transductivity sensitivity as that in AML cells. The results showed that the sign changes of transductivity sensitivities occurred between TRAF2 and ASK1, and between MKP and JNK. The genetic mutations in these two interaction sites may result in attenuating the crosstalk between TNF and TGF signaling pathways, which may lead to the difference in regulation of p38 during erythroid differentiation between AML and MDS cells [34]. Similarly, the negative transductivity sensitivities of p53 in AML and MDS cells were due to the genetic mutations of the interaction sites between JNK and p53, and between RSK2 and p53, which lead to the dysregulation of apoptosis in leukemic subtypes. The positive transductivity sensitivity of AKT in the PI3K-AKT pathway potentially increased MDM2-mediated apoptosis. However, the attenuated apoptosis, cell cycle arrest, and DNA repair in MDS were partly due to the genetic mutation of MDM2, which lead to sign change of transductivity sensitivity between AKT and MDM2 in MDS cells.

### Genetic mutation of JAK, epigenetic regulation of ERK, and signal integration of C/EBPα contributing to the promotion of MDS cell differentiation

The positive transductivity sensitivities of STATs in AML, and MDS cells, 0.7037 and 0.8238 respectively, indicated that STATs significantly mediated signal transmission in inhibiting apoptosis and promoting cell cycle progression and cell proliferation at AML and MDS (Figure 7 and Supplementary Table 1). Moreover, the low transductivity of STATs in normal type, 0.1456, indicated that large amount of energy of the signals from all external signals were attenuated by STATs in normal type. STATs was more sensitive to all external stimuli in leukemic than in normal type. The different roles of STATs at AML, MDS, and normal type were due to the genetic mutation of JAK at AML, and the epigenetic regulations of ERK in AML and MDS cells, which can be supported by the results using next-generation sequencing data analysis [35] and quantitative DNA methylation analysis [33].

The positive transductivity sensitivities of AP-2α and c-Fos at AML and MDS indicated that compared to normal type, eventually leading to leukemogenesis. The positive transductivity sensitivities of c-Fos and AP-2α were due to the identified epigenetic regulation of ERK, which leads to the inhibited apoptosis and the promoted cell cycle progression and cell proliferation in leukemic subtypes. It has been proposed that the dephosphorylation of ERK is associated with its activity to inhibit apoptosis [36] and promote cell growth [37] in leukemic cells. Furthermore, the result suggested that RAS at AML and MDS mediated the signal transmission of the cell proliferation between the upstream proteins, PDGFR, FLT3, KIT, and Shc, and the downstream proteins, RAF and MEK. RAS signaling also influenced PI3Kc and PIP3 signaling. Figure 7 suggested the genetic mutation of PTEN at MDS, which dephosphorylated PIP3 at AML [38]. Therefore, we suggested that PTEN acts as a brake to prevent the signal transduction of PIP3 in AML subtype and normal type.

Additionally, we also suggested that the repressed cell differentiation in AML subtype and the activated cell differentiation in normal type were due to the epigenetic modification of AML1 and the signal integration of C/EBPα, respectively. It has also been proposed that AML1 can block myeloid differentiation through histone modification [39].

### Genetic mutations of the corepressors CTBPs and SMRT leading to cell proliferation and differentiation AML and MDS leukemogenesis

The tumor suppressor function of Numb, which interacted with NOTCH and Deltex was associated with cell differentiation [40]. In Figure 8, the predicted genetic mutations of DVL, Numb and Deltex contribute to the dysregulation of NOTCH nuclear translocation. Additionally, the proposed coactivators, including p300, HATs, MAML, and SKIP [41], and corepressors, including CTBPs and SMRT [42], for mammalian Notch pathway reveal positive and negative transductivity sensitivities at both AML and MDS, respectively (Figure 8). The predicted mutations of the corepressors CTBPs and SMRT in leukemic cells lead to AML and MDS leukemogenesis, which has been discussed in carcinogenesis [43].

### Epigenetic regulation of β-catenin and genetic mutation of DVL contributing to the promoted cell proliferation of leukemic cells

In hematopoietic stem cells, the induced FOXO3a is essential for escaping quiescence, to enhance self-renewal ability, and to repress apoptosis [44]. The positive transductivity sensitivity of FOXO3a at MDS and the negative transductivity sensitivity of FOXO3a at AML were attributed to the genetic mutations in TRAF2, TRADD and the interaction site between AKT and GSK3β at AML, and the genetic mutations in TRADD and IKKs at MDS (Figure 9), which result in the increased apoptosis of MDS cells and the decreased apoptosis of AML cells compared to normal type. The results can also be supported by clinical observation [30].

Additionally, in AML subtype, the positive transductivity sensitivity of β-catenin resulted from mutant DVL, while in MDS subtype, it was influenced by not only mutant DVL but also epigenetic regulation of β-catenin and the impact of other pathways on Axin (Figure 9). A recent study supports the result that cooperative repression of Wnt pathway by SOX7 and Axin is attenuated in MDS patients [45]. β-catenin is hypermethylated in one third of MDS samples [46]. The result in Figure 9 reveals that the cross-talk between apoptosis and Wnt pathways was interrupted by the genetic mutations in IKKs at MDS and in the interaction site between DVL and GSK3β at AML. The interruption leads to high positive transductivity sensitivities of the destructive complex at AML. It implicates GSK3β and CK1 acted as brakes to repress the degradation of the destructive complex in normal type. In AML subtype, the accumulated β-catenin promotes cell proliferation through the dysregulated GSK3β and CK1.

### Comparing transductivity sensitivity with correlation sensitivity

In this study, we compared the results in transductivity sensitivity with a general assumption that the induced protein expression in the downstream of the coupled signaling pathways is positively or negatively correlated with the ligand protein expression of the pathways [47–49]. The results in correlation sensitivity (Figure 4A) showed that the correlation sensitivities of c-Fos/p53 in AML/MDS, PLZF in MDS become close to 0, and those of c-Jun in MDS, and PLZF in AML become negative when compared with the results in transductivity sensitivity. It has been found that c-Fos and c-Jun were associated with cell proliferation and differentiation [50–53], and p53 was associated with apoptosis in both AML and MDS [54, 55]. It has been also reported that PLZF was one of the highly expressed genes during osteoblastic differentiation [56], and PLZF was associated with cell differentiation in AML [57]. Therefore, the results in transductivity sensitivity (Figure 4B) were more consistent with the previous reports rather than correlation sensitivity (Figure 4A). It is because transductivity was calculated from the network model in (13) based on systems theory, which was different from the calculation of the correlation between two genes.

### Comparing transductivity sensitivities of 28 TFs between 2 datasets at MDS/AML

In order to test the reliability of the proposed results, we used the microarray raw data with sample size 187 of AML+nok/abn without normalization from the GEO database (accession no. GSE6891) [58] and the microarray raw data with sample size 164 of MDS without normalization from the GEO database (accession no. GSE15061) [59] to calculate the transductivity sensitivities of 28 TFs (Figure 3C and 3D). Owing to the experimental manipulation and individual difference characteristics, comparison of the results in the transductivity sensitivities of 28 TFs between 2 different datasets (Figure 3C and 3D) shows consistent trends in 9 proliferation-related TFs CSLs, p300, CTBPs, AP-2α, STATs, c-Jun, β-catenin, LEF-1 and c-Fos, the anti-apoptosis-related TF Elk-1, the cell cycle-related TF pRb, 2 differentiation-related TFs ETO and PLZF, and 3 self-renewal-related TFs Smad2, Smad3 and Smad4. According to top 3 TFs with the largest differences between two datasets in AML+nok/abn (Figure 3C) and in MDS (Figure 3D) including the apoptosis-related TF FOXO3a, the anti-apoptosis-related TF NF-κBs, the angiogenesis-related TF HIF-1α, the cell cycle-related TF E2Fs, and the differentiation-related TF PU.1, the TFs also have large differences between AML+nok/abn and MDS in the same dataset (Figure 3B). Therefore, we inferred that the 5 TFs FOXO3a, NF-κBs, HIF-1α, E2Fs and PU.1 with the largest differences in transductivity sensitivity result from the effect of individual difference on the induction of redundant cellular functions such as anti-apoptosis, angiogenesis, cell cycle and cell differentiation to promote AML/MDS leukemogenesis. According to the comparison of the results in transductivity sensitivity (Figures 3C and 3D), we suggested that the proposed results to compare the transductivity sensitivities between AML+nok/abn and MDS in the same dataset (GSE13159) are reliable.

Moreover, the dysregulated TFs in STPs were attributed to the identified genetic and epigenetic alterations of the proteins with the sign change of transductivity sensitivities in STPs. The identified proteins with genetic mutations or epigenetic alterations through transductivity sensitivity analysis, which lead to the dysregulated TFs to respond to external stimuli in leukemogenesis, are used as multiple drug target for preventing MDS or AML leukemogenesis. Table 1 shows the potential targets for treating AML and MDS and preventing the progression of MDS to AML. The full table for 159 proteins is shown in Supplementary Table 2.

Table 1Drug target identification during leukemogenesis

**Table:** A. Drug targets for the progression of normal to MDS

Drug target	Transductivity		Suggested treatment	A ratio of mean expression in MDS to normal	A variance of expression in patients		Functions in leukemogenesis (Dysfunction of TF)
	normal	MDS			MDS	normal	
C/EBP-α	0.0002	0.0361		0.7726	848251.61	19217040.95	v
PSEN	0.0987	0.3008	inhibitor	**1.0179**	248411.94	167573.03	ii (CSLs)
NCSTN	0.0416	0.1530	inhibitor	**1.2328**	24603.49	18137.11	ii (CSLs)
IKKs	0.0011	0.0537	inhibitor	**1.1028**	12665.02	7749.33	ii,iii (FOXO3a, **β-catenin**)

**Table:** B. Drug targets for the progression of normal to AML+nok/abn

Drug target	Transductivity		Suggested treatment	A ratio of mean expression in MDS to normal	A variance of expression in patients		Functions in leukemogenesis (Dysfunction of TF)
	normal	AML+nok/abn		AML+nok/abn	AML+nok/abn	normal	
JAK	0.1532	0.3145	inhibitor	0.7109	4898376.06	2360573.35	ii, iii (STATs)
TRAF2	0.0111	0.0020	angonist	**1.2894**	619492.22	111782.08	i, iii (p53)
CK1	0.0106	0.0351	inhibitor	**1.2629**	59237.12	8404.98	ii (**β-catenin**)
AML1	0.0347	0.0134		0.9662	10077.42	4985.50	iv, iii (C/EBP-α)

**Table:** C. Drug targets for the progression of MDS to AML+nok/abn

Drug target	Transductivity		Suggested treatment	A ratio of mean expression in MDS to normal		A variance of expression in patients			Drug target with mutations accumulated at upstream proteins for progression of MDS to AML+nok/abn	Functions in leukemogenesis (Dysfunction of TF)
	MDS	AML+nok/abn		MDS	AML+nok/abn	normal	MDS	AML+nok/abn		
HATs	0.011	0.041	inhibitor	**1.053**	0.950	368974.9	790829.9	1209579.2	^*^	ii (CSLs)
p38	0.028	0.001	agonist	**1.001**	0.989	227582.0	215526.5	246799.2	^*^	i (p53)
DAXX	0.542	0.004	agonist	**1.005**	0.676	217649.0	282772.2	132876.4		i (p53)
Numb	0.049	0.004	agonist	**1.064**	**1.227**	6602.0	7642.0	16924.1		iii, iv
SMRT	0.007	0.001	agonist	**1.084**	**1.169**	5876.6	9131.0	10391.0	^*^	ii, iii (CSLs)
GSK3β	0.059	0.295	inhibitor	**1.031**	0.957	9139.6	13001.6	10144.9		ii (**β-catenin**)
TGFβR	0.331	0.050	agonist	0.810	0.663	27157.9	16646.1	8484.0		i (p53)
Grb2	0.181	0.541	inhibitor	0.873	0.996	2152.2	1702.7	3478.1		ii (STATs)

Functions in leukemogenesis: i. Proteins may transduce less signals to respond to either the stress or apoptotic signals to cause the deficiency of defensive mechanism like DNA repair. ii. Proteins may transduce more signals in some pathways to facilitate to proliferation and anti-apoptosis. iii. Protein may be affected by their genetic mutations to dysregulated the signal transduction of STPs. iv. Protein may transduce less signals in the pathways to facilitate to differentiation.

Furthermore, we calculated fold changes and variances of mRNA expressions in each drug target to support the effectiveness of these drug targets based on transductivity sensitivity analysis. If a drug target has large fold change and small variance in its mRNA expression at MDS or AML compared to normal type, it implicates that the activity of the drug target is activated in most of MDS or AML patients. The activated drug targets PSEN, NCSTN and IKKs in Table 1A and the activated drug targets TRAF2 and CK1 in Table 1B are considered as the most effective drug target for preventing MDS and AML leukemogenesis, respectively.

Additionally, we identified the increasingly accumulated genetic and epigenetic changes of the proteins from MDS to AML in Table 1C. According to the result in Table 1C, the activated drug targets HATs, p38 and SMRT are considered as the most effective drug target for preventing the potential progression of MDS to AML.

### Potential drug repurposing for AML/MDS based on transductivity sensitivity using drug response genome-wide microarray data

In order to design drugs for treating patients with AML and MDS based on transductivity sensitivities of 28 TFs (see Materials and Methods) using drug response genome-wide microarray data, we firstly calculated the transductivity sensitivities of 28 TFs (Supplementary Figure 3) for 1327 drugs in Connectivity Map (CMap) [60]. Because most drugs have less sample sizes (*N* < 100) in the corresponding microarray data, we calculate the effectiveness (*E_j_*) of the drugs, which have been identified to effectively treat patients with AML and MDS. According to the effectiveness (*E_j_*) of the well-identified drugs, we finally proposed two drugs, which could be effective therapies for treating patients with AML and MDS, respectively.

**Figure 10:**
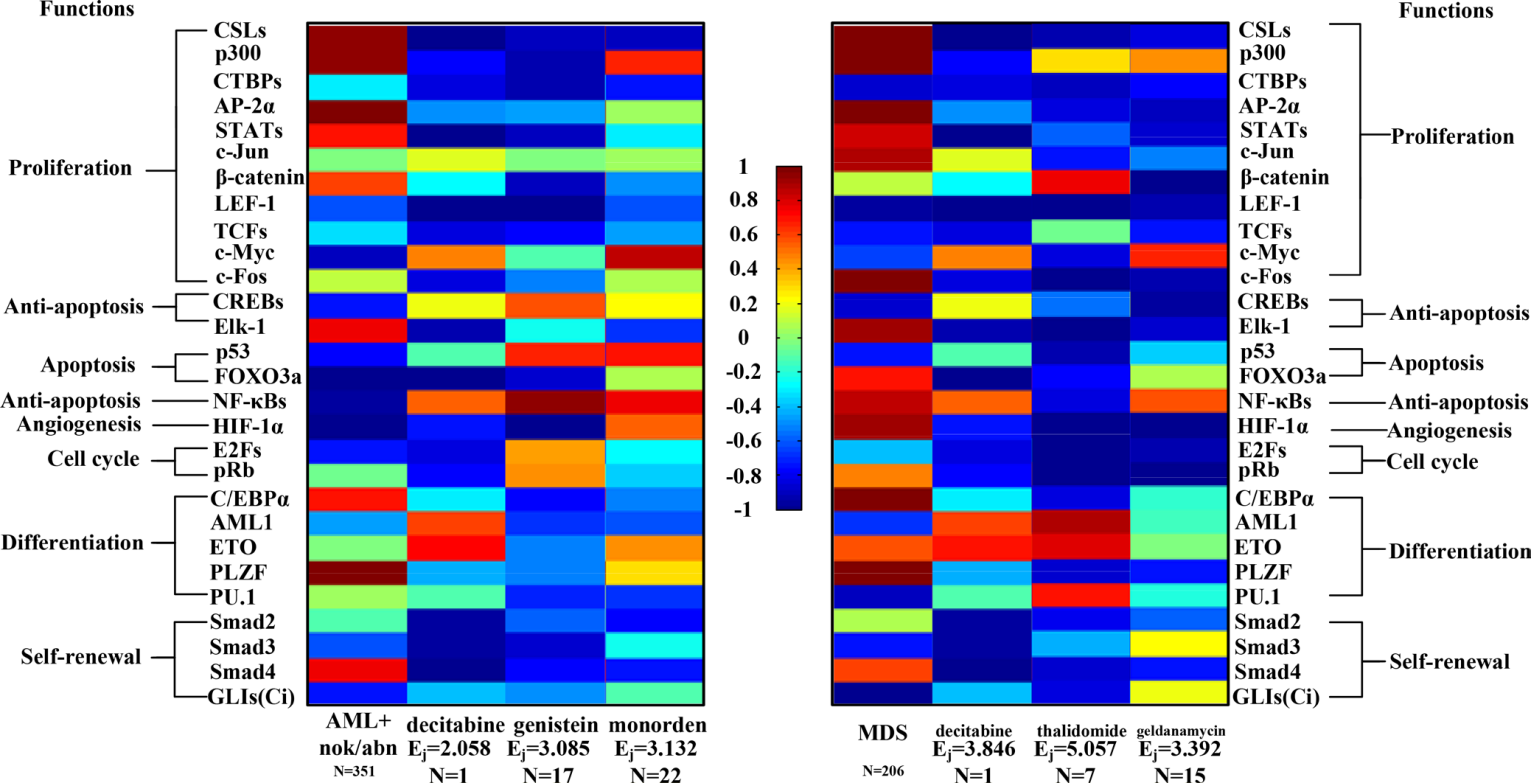
Transductivity sensitivities of 28 TFs in AML, MDS, and the proposed drugs We proposed three drugs, decitabine, genistein, and monorden, with effectiveness (*E_j_* = 2.058, 3.085, and 3.132, respectively) for treating patients with AML, while three drugs, decitabine, thalidomide, and geldanamycin, with effectiveness (*E_j_* = 3.846, 5.057, and 3.392, respectively) for treating patients with MDS. The transductivity sensitivities of 28 TFs in three drugs are inversely correlated with the transductivity sensitivities of 28 TFs in the corresponding leukemic subtype. The full table of effectiveness of drugs for treating patients with AML/MDS is shown in Supplementary Table 4.

Decitabine, a DNA-hypomethylating agent that induces differentiation and apoptosis of leukemic subtypes, is a well-tolerated alternative to aggressive chemotherapy for the treatment of cancer cells. The U.S. Food and Drug Administration (FDA) has approved decitabine to treat MDS. AML is a disease of the elderly, with a mean age of diagnosis of 70 years. Adverse cytogenetic abnormalities increase with age, and within each cytogenetic group, prognosis with standard treatment worsens with age. The use of decitabine in the treatment of patients with AML has been studied. Because the survival difference did not reach significance, decitabine did not achieve FDA approval for AML, but continues to be used off-label. It has been reported that Elk-1 and AML1 mediate decitabine-reduced anti-apoptosis [61] and decitabine-induced differentiation [62] in leukemic subtypes, respectively. The results that decitabine reduces and induces the transductivity sensitivities of Elk-1 and AML1, respectively, support the previous observation (Figure 10). A recent study has also proposed that genistein could be an effective alternate therapy for AML to induce cell death via apoptosis [63]. It has been observed that genistein enhances the phosphorylation and activation of p53, which result in cells undergoing apoptosis [64]. It could be supported by our result that genistein induces the transductivity sensitivity of p53 (Figure 10). Thalidomide, which has been approved by the FDA to treat MDS, has considerable therapeutic efficacy in multiple myeloma, possibly because of its anti-angiogenetic properties in the bone marrow [65]. It has been also observed that thalidomide inhibited angiogenesis via the down-regulation of HIF-1α expression [66]. The reduced transductivity sensitivity of HIF-1α by thalidomide supported the anti-angiogenetic properties of thalidomide in the bone marrow. We then applied drug's effectiveness based on transductivity sensitivity to 28 TFs of AML/MDS to propose the potential drug for treating patients (see Materials and Methods). Therefore, the results in transductivity sensitivities of TFs by applying different drugs (with *E_j_* ≥ 2.058) could be validated by the anti-cancer drugs for treating patients with AML and MDS. Furthermore, in order to find the potential anti-cancer drugs, we selected monorden and geldanamycin, which have large drug's effectiveness (*E_j_* = 3.132 and 3.392, respectively) and large sample sizes (*N* = 22 and 15, respectively), for treating AML and MDS, respectively. Internal tandem duplication mutations of the FMS-like tyrosine kinase 3 (FLT3) gene (FLT3-ITD) has been discovered in AML with frequencies of 20%, which carry a poor prognosis. It has been observed that the treatment with monorden (alias radicicol) induced apoptosis in FLT3-ITD-transformed myeloid progenitor 32D cells [67]. Our result showed that the transductivity sensitivity of p53 was reduced by monorden. It has been observed that radicicol-induced cell death was mediated by p53 activation [68]. It has been reported that a derivative of the geldanamycin, 17-N-allylamino-17-demethoxy geldanamycin (17-AAG), one of the original and most studied heat shock protein 90 (HSP90) inhibitors, can reduce the abnormal VEGF expression produced by the stromal cells from children with MDS by blocking the ability of the MDS stromal cells to stimulate the growth of leukemic subtypes [69]. In the results (Figure 10), we identified that the transductivity sensitivities of CSLs, AP-2α, STATs, c-Jun, c-Fos, Elk-1, HIF-1α, and PLZF were reduced by geldanamycin. Geldanamycin-inhibited expressions of AP-2α [70], HIF-1α [71, 72], c-Jun [73], STATs [71, 74], and Elk-1 [75] have been reported to reduce the cell survival and proliferation, while HSP90-inhibited expression of CSLs [76], c-Fos [77], and PLZF [3] has also been reported to reduce the cell proliferation and differentiation. Therefore, according to the analysis of the transductivity sensitivity using drug response genome-wide microarray data, we proposed the drugs, decitabine, genistein, and monorden, for treating patients with AML and the drugs, decitabine, thalidomide, and geldanamycin, for treating patients with MDS.

## MATERIALS AND METHODS

A flowchart for estimating network robustness of the GRNs and for predicting impact of genetic mutations, epigenetic alterations and the coupling of other pathways of the STPs in Supplementary Figure 1 is presented in Figure 1. These processes can be divided into six steps: 1) data selection and preprocessing of gene expression microarray sample data; 2) rewiring the leukemogenesis-related coupling STPs and its associated GRN (Supplementary Figure 1); 3) the construction of GRN and calculation of the network robustness of GRN; 4) the construction of coupling STPs and calculation of transductivity, transductivity sensitivity, basal sensitivity, and error sensitivity in STPs; 5) the prediction of the impact of genetic mutation, epigenetic alteration and the coupling of other pathways by Figure 5; and 6) the suggestion of potential drugs based on transductivity sensitivity, using drug response genome-wide microarray data. The involved materials and methods are described stepwise in the following.

### Data selection and preprocessing of gene expression microarray sample data

We used microarray raw data without normalization from the GEO database (accession no. GSE13159). To technically support clinical diagnosis of hematological malignancies, *Haferlach et al.* [59] obtained gene expression profiles of 2096 patients from 11 laboratories to identify biomarkers and validate their clinical accuracy. Of these 2096 patients, 2022 were classified into 17 leukemic subtypes and 74 were classified as being normal. To prevent the overfitting and inaccuracy in identifying model parameters, we selected 7 leukemic subtypes that had a greater number of samples than the number of samples for the normal type (Figure 3). In addition, while constructing the models of integrated cellular network of coupling STPs and GRNs, we overlaid the expression value of each gene on its corresponding protein to integrate gene expression and PPI information.

### Rewiring the leukemogenesis-related coupling STP and its associated GRN

Several studies [1, 3, 4, 7, 17–19] have constructed STPs that participate in leukemogenesis and have used the Kyoto Encyclopedia of Genes and Genomes (KEGG) database [20, 21] to partially identify the relationships among kinases in these STPs. Leukemogenic STPs include the PI3K-AKT, classical MAPK, JAK-STAT, TGFβ, apoptosis, Notch, hedgehog, and canonical Wnt pathways (Supplementary Figure 1). In leukemic subtypes, signal transduction mediated by binding of ligands to STP receptors results in their abnormal activation because of genetic mutations in genes encoding receptors, kinases, and phosphatases involved in these STPs [4]. Cross-talk, which is a convergence between various pathways, can also influence and dysregulate STPs because of the effects of upstream mutations [1, 4]. For example, GSK3β is crucial for both the Wnt and PI3K-AKT pathways. Aberrant or constitutive activation of these pathways can trigger dysfunctions by activating or repressing TFs. The resultant events facilitate cell proliferation, prevent apoptosis, and block hematopoietic cell differentiation. Aberrant activation of pathways such as the Wnt and Notch pathways favors self-renewal of LSCs, which is crucial for myeloid malignancy [1]. In addition, genetic mutations not only enhance tumor malignancy but also resist the effects of cancer therapies [1, 2, 4]. Collectively, dysfunctioning of proliferation, prevention of apoptosis, blockage of differentiation, and maintenance of LSCs are involved in the pathophysiology of leukemia. To investigate whether signal transductivity of pathophysiological STPs is distorted in leukemic subtypes and whether proteins in STPs receive exact information from the coupling pathways, we constructed a system model of coupling STPs containing 159 groups of proteins and 28 groups of TFs (Supplementary Figure 1 and Supplementary Table 3) [1, 3, 4, 7, 10, 17, 19, 78]. We focused on the transductivity of downstream TFs p53, C/EBPα, ETO, STATs (STAT1, STAT3, and STAT5), AP-2α, c-Jun, CSLs (RBP-JK and RBPJL), p300, CTBPs, β-catenin, and FOXO3a to discuss whether the signal transductivity of pathophysiological STPs is distorted or not (see Discussion). Transcriptional regulation of GRNs was determined using the transcription factor database (TRANSFAC; http://gene-regulation.com/) [22]. We selected genes encoding 18 TFs that regulated the transcription of 28 TFs to construct a GRN and to calculate its robustness in the 7 leukemic subtypes and 1 normal type (Supplementary Figure 2). The protein-encoding genes corresponding to 18 TFs included AP-2α, β-catenin, C/EBPα, p300, c-Myc, CREBs (ATF4, CREB1, Luman, OASIS, CREB3L2, CREB-H, AibZIP, and CRE-BPa), CSLs, E2Fs (E2F1, E2F2, and E2F3), Elk-1, c-Fos, HIF-1α, c-Jun, LEF-1, NF-κBs (NF-κB1, NF-κB1-p50, NF-κB2-p52, NF-κB2, NF-κB2-p52, c-Rel, RelA-p65, and RelB), Smad3, STATs, TCFs (TCF-1, TCF-3, and TCF-4), and p53.

### Construction of GRN and calculation of the network robustness of GRN

#### Construction of GRN by microarray data from a population of samples

For the purpose of the system identification of GRN from a population of leukemia samples, we use a simple regression model, GRN model, to depict transcription regulations in GRN (Supplementary Figure 2). It is assumed that the GRN consists of *M* genes of TFs (*M* = 18, in Supplementary Figure 2). We use microarray data from a population of leukemia samples with one time-point microarray data for each individual patient to construct GRN. The samples of microarray data are derived from *K* patients. Let the state variable of the *j*^th^ gene of TF in GRN be denoted as *y_j_*(*k*). In the steady state case, the expressions of GRN can be modeled as the following linear regression equation,

$$\begin{array}{l} y_{j}(k)=c_{j,1}y_{1}(k)+\cdots +c_{j,j-1}y_{j-1}(k)+c_{j,j+1}y_{j+1}(k)+\cdots \\ \;+c_{j,M}y_{M}(k)+h_{j}+w_{j}(k),\text{ for }j=1,\cdots ,M\text{ and }k=1,\cdots ,K \end{array}$$ (1)

where *y_j_*(*k*) indicates the expression level of the *j*^th^ gene of TF in the GRN at the *k*^th^ clinical sample; *c_j_*_,*j*-1_ is transcription regulatory ability from (*j*-1)^th^ TF to the *j*^th^ gene of TF. If *c_j_*_,*j*-1_ is positive, it means the TF *j*-1 performs an activating regulation on gene *j*. Otherwise it is an inhibitive regulation; *h_j_* denotes the basal level of the *j*^th^ gene expression; *w_j_*(*k*) represents model uncertainties of the *j*^th^ gene. Note that *c_j_*_,*j*_ representing the transcription regulatory ability from the *j*^th^ TF to its gene is set to be zero in this method. The model (Eqn. 1) biologically states that the gene expression *y_j_*(*k*) of the target gene *j* is associated with the expressions of its regulatory TFs.

#### Identification of parameters in GRN model by microarray sample data

To identify the regulatory parameters of (Eqn. 1) with the constraint *h_j_* ≥ 0, i.e. *θ_j_*=[*c_j_*_,1_, ^…^, *c_j_*_,*M*_, *h_j_*]^T^, we formulate the linear regression models in (Eqn. 1) as the following forms

$$\begin{array}{l} y_{j}(k)=\left[\begin{array}{ccccccc} y_{1}(k) & \cdots & y_{j-1}(k) & y_{j+1}(k) & \cdots & y_{M}(k) & 1 \end{array}\right]\cdot \left[\begin{array}{c} c_{j,1}\\ \vdots \\ c_{j,j-1}\\ c_{j,j+1}\\ \vdots \\ c_{j,M}\\ h_{j} \end{array}\right]+w_{j}(k)\\ \;=\phi_{j}^{T}(k)\theta_{j}+w_{j}(k),\text{ for }j=1,\cdots ,M\text{ and }k=1,\cdots ,K \end{array}$$ (2)

Because microarray data from a population of samples is used to identify the parameters of the gene regulatory models, we use recursive least-squares identification to estimate the parameter *θ_j_* one sample by one sample. The recursive identification algorithm for estimating ${\hat{\theta }}_{j}(k)$ of the regulatory parameters *θ_j_* of the *j*^th^ gene in (Eqn. 2) at the *k*^th^ step is given as follows [23]:

$$\begin{array}{l} {\overset{\wedge }{\theta }}_{j}(k)={\overset{\wedge }{\theta }}_{j}(k-1)+P_{j}(k)\phi_{j}(k)\varepsilon_{j}(k)\\ \varepsilon_{j}(k)=y_{j}(k)-\phi_{j}^{T}(k){\overset{\wedge }{\theta }}_{j}(k-1)\\ P_{j}(k)=P_{j}(k-1)-\frac{P_{j}(k-1)\phi_{j}(k)\phi_{j}^{T}(k)P_{j}(k-1)}{1+\phi_{j}^{T}(k)P_{j}(k-1)\phi_{j}(k)}\text{, }{\overset{\wedge }{\theta }}_{j}(0),P_{j}(0\text{) given}\\ \text{ , for }j=1,\cdots ,M\text{ and }k=1,\cdots ,K \end{array}$$ (3)

where ${\overset{\wedge }{\theta }}_{j}(k)$ is the parameter estimate of θ_*j*_ and *ε_j_*(*k*) is the prediction error at the *k*^th^ step. The matrix *P_j_*(*k*) constitutes an estimate of the parameter estimation error covariance at recursive step *k*. Initially, *P_j_*(0) is chosen as *diag*(10,…,10)_*M*×*M*_ for GRN model because of large estimation error at the initial estimate ${\overset{\wedge }{\theta }}_{j}(0)$. The dimension of *P_j_*(0) is determined by (*M+*1) regulatory parameters for each protein in GRN model needed to be estimated. We identify the regulatory parameters of linear regression model one gene by one gene (i.e. *j* = 1,…, *M* in (Eqn. 3)) to obtain the whole regulatory parameters of GRN.

#### Remark 1

For more precise parameter estimation in (Eqn. 3), the result of signal parameter estimate ${\overset{\wedge }{\theta }}_{j}(k)$ after *K* iterations could be considered as the initial parameter estimate ${\overset{\wedge }{\theta }}_{j}(0)$ in (Eqn. 3) for another round of recursive parameter estimation.

By using the recursive identification algorithm in (Eqn. 3), the parameter estimate ${\overset{\wedge }{\theta }}_{j}(k)$ of transcription regulatory abilities for each gene in GRN can be updated as the new samples of microarray data are measured. Therefore, the recursive identification algorithm in (Eqn. 3) is suitable for identifying the parameters of the regression model for GRN by microarray sample data.

### Estimation of the network robustness of GRN

The network robustness in GRN is a measurement of the system performance of biological networks to tolerate intrinsic variations like genetic mutation to maintain their phenotype. From an engineering perspective, network robustness and transductivity are two important and complementary system characteristics to discuss system performance, i.e. a more robust system will be with a less transductivity, and vice versa [15, 24]. To systematically measure the network robustness in GRN, the one gene regulatory model of GRN in (Eqn. 1) must be augmented for *M* genes as the following multivariate regulatory model.

$$y=\overset{\wedge }{C}y+\overset{\wedge }{H}+W$$ (4)

where

$$\begin{array}{l} y={\left[\begin{array}{ccc} y_{1}(k) & \cdots & y_{M}(k) \end{array}\right]}^{T};\;\overset{\wedge }{H}={\left[\begin{array}{ccc} {\overset{\wedge }{h}}_{1} & \cdots & {\overset{\wedge }{h}}_{M} \end{array}\right]}^{T};\;W={\left[\begin{array}{ccc} w_{1}(k) & \cdots & w_{M}(k) \end{array}\right]}^{T}\\ \; \end{array}$$

and

C∧=[0c∧1,2⋯c∧1,j−1c∧1,jc∧1,j+1⋯c∧1,Mc∧2,10⋱⋯⋯⋯⋯c∧2,M⋮⋱⋱⋱⋯⋯⋯⋮c∧j−1,1⋯⋱0c∧j−1,j⋯⋯c∧j−1,Mc∧j,1⋯⋯c∧j,j−10c∧j,j+1⋯c∧j,Mc∧j+1,1⋯⋯⋯c∧j+1,j0⋱c∧j+1,M⋮⋯⋯⋯⋯⋱⋱⋮c∧M,1⋯⋯⋯⋯⋯⋱0]

Let us denote *y* as the expression vector of *M* genes in GRN; $\overset{\wedge }{C}$ represents the system matrix of transcription regulatory ability in GRN; $\overset{\wedge }{H}$ represents the vector of basal levels for *M* genes; $\overset{\wedge }{C}$ and $\hat{H}$ consist of the parameters identified by the recursive identification algorithm in (Eqn. 3) through *K* samples of microarray data; *W* represents the vector of model uncertainties for *M* genes. (Eqn. 4) can be rearranged as follows:

$$(I_{M}-\overset{\wedge }{C})y=\overset{\wedge }{H}+W$$ (5)

And the gene expression vector *y* in GRN can be represented by

$$y={\left(I_{M}-\overset{\wedge }{C}\right)}^{-1}\left[\overset{\wedge }{H}+W\right]$$ (6)

where $\left(I_{M}-\overset{\wedge }{C}\right)$ represents the transcription regulatory matrix in GRN and $I_{M}$ denotes identity matrix with dimension *M*.

### Proposition 1

The network robustness ξ of GRN in a leukemia or normal type is estimated as follows:

$$\xi =\underset{j}{\min}\;\sigma_{j}(I_{M}-\overset{\wedge }{C})≜\sigma_{\min}(I_{M}-\overset{\wedge }{C})$$ (7)

where $\sigma_{j}$ denotes the $j^{th}$ singular value of $\left(I_{M}-\overset{\wedge }{C}\right)$ and $\sigma_{\min}$ denote the minimum singular value.

Proof: See Proof S1 in Supplementary Materials.

### Remark 2

Let us decompose $\left(I_{M}-\overset{\wedge }{C}\right)$ by the following singular value decomposition [23].

$$\left(I_{M}-\overset{\wedge }{C}\right)≜\tilde{C}=\sum_{j=1}^{M}\sigma_{j}u_{j}{}^{T}v_{j}=U^{T}\Sigma V=U^{T}\cdot diag\left(\begin{array}{ccc} \sigma_{\max}(\tilde{C}), & \cdots & ,\sigma_{\min}(\tilde{C}) \end{array}\right)\cdot V$$ (8)

where $\sigma_{j}$ denotes the *j^th^* singular value and is ordered decreasingly. $\text{i.e}.\;\sigma_{1}(\tilde{C})=\sigma_{\max}(\tilde{C})\geq \sigma_{2}(\tilde{C})\geq \cdots \geq \sigma_{M}(\tilde{C})=\sigma_{\min}(\tilde{C})$. If all singular values are free of zero, the inverse of $\tilde{C}$ exists. That is, the steady state of this GRN model in (6) exists. If at least one singular value of $\tilde{C}$ is perturbed to 0, the inverse of $\tilde{C}$ doesn't exist.

### Remark 3

Let us decompose the transcription regulatory matrix $I_{M}-\overset{\wedge }{C}-\Delta C$ of perturbative GRN under intrinsic perturbation by the singular value decomposition. If the intrinsic parameter variation Δ*C* is specified as $\Delta C=\sigma_{\min}(I_{M}-\overset{\wedge }{C})I_{M}$, which violates the robustness condition in (S7). Then from (Eqn. 8), the inverse of $I_{M}-\overset{\wedge }{C}-\Delta C$ will cease to exist, i.e.

$$\left(I_{M}-\overset{\wedge }{C}-\Delta C\right)=\tilde{C}-\Delta C=U^{T}\cdot diag\left(\begin{array}{ccc} \sigma_{\max}(\tilde{C}), & \cdots & ,0 \end{array}\right)\cdot V$$ (9)

It means that the model of GRN can't tolerate the intrinsic parameter variation and the phenotype $y={\left(I_{M}-\overset{\wedge }{C}-\Delta C\right)}^{-1}[\overset{\wedge }{H}+\;W\;]$ doesn't exist again. Hence, the robustness measurement ξ of the GRN is defined as

$$\xi =\sigma_{\min}(I_{M}-\overset{\wedge }{C})$$ (10)

### Construction of coupling STPs and calculation of transductivity, transductivity sensitivity, basal sensitivity, and error sensitivity in STPs

#### Construction of coupling STPs by microarray sample data

For the purpose of the system identification of the coupling STPs from a population of leukemia samples, we use the other simple regression model, STP model, to depict protein-protein interactions and the bindings of ligands to receptors in the coupling STPs (Supplementary Figure 1). It is assumed that the coupling STPs consist of *N* proteins (*N* = 159, in Supplementary Figure 1). We use microarray sample data to construct the STPs. The samples of microarray data are derived from *K* patients. Let the state variable of the *j^th^* protein and its ligand in the STPs be denoted as *x_i_* (*k*) and $u_{i}(k)$, respectively. In the steady state case, the PPI model for the STPs in Supplementary Figure 1 can be modified as the following linear regression model.

$$\begin{array}{l} x_{i}(k)=a_{i,1}x_{i}(k)+\cdots +a_{i,i-1}x_{i-1}(k)+a_{i,i+1}x_{i+1}(k)+\cdots \\ \;+a_{i,N}x_{N}(k)+b_{i}u_{i}(k)+d_{i}+e_{i}(k),\text{ for }i=1,\cdots ,N\text{ and }k=1,\cdots ,K \end{array}$$ (11)

where *x_i_* (*k*) and $u_{i}(k)$ indicate respectively the expression levels of the *i^th^* protein and its ligand in the STPs at the *k^th^* clinical sample; *a_i,j_*_–1_ and *b_i_* respectively represent the interaction abilities between the *i^th^* and (*i*–1)*^th^* proteins and the binding ability between the *i^th^* protein and its ligand; *d_i_* denotes basal levels of the *i^th^* expression levels; *e_i_*(*k*) represents stochastic noises associated with other factors of the *i^th^* protein in other pathways; If the *i^th^* protein doesn't serve as a receptor, *b_i_* is set to be zero. Note that the interaction ability *a_i,i_* of the *i^th^* proteins itself is set to be zero in this method. The model in (Eqn. 11) states that, biologically, the expression level *x_i_* (*k*) of the target protein *i* is associated with the expression levels of its ligand and interactive proteins; It is worthy to note that the interaction parameters *a_i,j_*, *d_i_* and *b_i_* of regression model (Eqn. 11) in the STPs are identified systematically according to microarray data in the following. Note that *i* ≠ *j,* for *j* = 1,^...^,*N*.

#### Identification of parameters in STP model by microarray sample data

To identify the protein interaction parameters of (Eqn. 11), i.e.$\theta_{i}$, we formulate the models in (Eqn. 11) as the following forms

$$\begin{array}{l} x_{i}(k)=\left[\begin{array}{cccccccc} x_{1}(k) & \cdots & x_{i-1}(k) & x_{i+1}(k) & \cdots & x_{N}(k) & u_{i}(k) & 1 \end{array}\right]\cdot \left[\begin{array}{c} a_{i,1}\\ \vdots \\ a_{i,i-1}\\ a_{i,i+1}\\ \vdots \\ a_{i,N}\\ d_{i}\\ b_{i} \end{array}\right]+v_{i}(k)\\ =\phi_{i}^{T}(k)\theta_{i}+v_{i}(k),\text{ for }i=1,\cdots ,N\text{ and }k=1,\cdots ,K\; \end{array}$$ (12)

Because microarray data from a population of samples is used to identify the parameters of the protein interaction models of the STPs in Supplementary Figure 1, we use recursive least-squares identification algorithm in (Eqn. 3) to estimate the interactive parameter $\theta_{i}$ of linear interaction model (Eqn. 11) of the *i^th^* protein in the STPs. We identify the interaction parameters of each protein of STPs with other proteins and ligands in Supplementary Figure 1 by the recursive least square algorithm in (Eqn. 3) one protein by one protein. Then we could estimate the whole interactive parameters of the STPs in Supplementary Figure 1. After whole protein-protein interaction abilities of leukemogenesis-related STPs in Supplementary Figure 1 are constructed by microarray data, we will investigate the transductivity, transductivity sensitivity, basal sensitivity, and error sensitivity of leukemogenesis-related coupling STPs in the following subsection.

#### Calculation of the transductivity, transductivity sensitivity, basal sensitivity, and error sensitivity of the proteins in the leukemogenesis-related coupling STPs

The transduction ability of a protein in the coupling STPs, termed as transductivity, measures the system response to environmental changes like environmental stress or the ability to transduce from the external molecular signals to other interactive proteins. To systematically measure the transductivity in the STPs in Supplementary Figure 1, we augment the PPI model of STPs in (Eqn. 11) for *N* proteins as the following multivariate regression model.

$$x=\overset{\wedge }{A}x+\overset{\wedge }{D}u+\overset{\wedge }{B}+E$$ (13)

Where $\begin{array}{l} x={\left[\begin{array}{ccc} x_{1} & \cdots & x_{N} \end{array}\right]}^{T}\;\;\;\;;\;\;\;\;\overset{\wedge }{D}=diag(\begin{array}{ccc} {\overset{\wedge }{d}}_{1}, & \cdots , & {\overset{\wedge }{d}}_{N} \end{array})\;\;\;;\;\;\;u={\left[\begin{array}{ccc} u_{1} & \cdots & u_{N} \end{array}\right]}^{T};\\ \overset{\wedge }{B}={\left[\begin{array}{ccc} {\overset{\wedge }{b}}_{1}\; & \cdots & \;{\overset{\wedge }{b}}_{N} \end{array}\right]}^{T}\;\;;\;\;\;E={\left[\begin{array}{ccc} e_{1} & \cdots & e_{N} \end{array}\right]}^{T} \end{array}$

and $\overset{\wedge }{A}=\left[\begin{array}{cccccccc} 0 & {\overset{\wedge }{a}}_{{}_{1,2}} & \cdots & {\overset{\wedge }{a}}_{{}_{1,i-1}} & {\overset{\wedge }{a}}_{{}_{1,i}} & {\overset{\wedge }{a}}_{{}_{1,i+1}} & \cdots & {\overset{\wedge }{a}}_{{}_{1,N}}\\ {\overset{\wedge }{a}}_{{}_{2,1}} & 0 & \ddots & \cdots & \cdots & \cdots & \cdots & {\overset{\wedge }{a}}_{{}_{2,N}}\\ \vdots & \ddots & \ddots & \ddots & \cdots & \cdots & \cdots & \vdots \\ {\overset{\wedge }{a}}_{{}_{i-1,1}} & \cdots & \ddots & 0 & {\overset{\wedge }{a}}_{{}_{i-1,i}} & \cdots & \cdots & {\overset{\wedge }{a}}_{{}_{i-1,N}}\\ {\overset{\wedge }{a}}_{{}_{i,1}} & \cdots & \cdots & {\overset{\wedge }{a}}_{{}_{i,i-1}} & 0 & {\overset{\wedge }{a}}_{{}_{i,i+1}} & \cdots & {\overset{\wedge }{a}}_{{}_{i,N}}\\ {\overset{\wedge }{a}}_{{}_{i+1,1}} & \cdots & \cdots & \cdots & {\overset{\wedge }{a}}_{{}_{i+1,i}} & 0 & \ddots & {\overset{\wedge }{a}}_{{}_{i+1,N}}\\ \vdots & \cdots & \cdots & \cdots & \cdots & \ddots & \ddots & \vdots \\ {\overset{\wedge }{a}}_{{}_{N,1}} & \cdots & \cdots & \cdots & \cdots & \cdots & \ddots & 0 \end{array}\right]$ represents the system matrix of PPIs in the STPs; $\overset{\wedge }{A}$

where *x* indicates the expression vector for *N* proteins in the STPs; $\overset{\wedge }{D}$ is a diagonal matrix and represents the system matrix of binding abilities between the receptor proteins and their ligands in the STPs. *μ* denotes expression vector of ligands in the STPs as input signals; $\overset{\wedge }{B}$ represents the vector of basal levels for *N* proteins; and $\overset{\wedge }{A}$, $\overset{\wedge }{B}$ and $\overset{\wedge }{D}$ consist of the interaction parameters identified by the recursive identification algorithm; *E* represents the vector of stochastic noises (or estimation errors) associated with other interaction factors for *N* proteins in other pathways. The model in (13) can be rearranged as follows:

$$(I_{N}-\overset{\wedge }{A})x=\overset{\wedge }{D}u+\overset{\wedge }{B}+E$$ (14)

Since we want to measure the transductivity of protein of interest in the STPs, we denote the expression level of the protein of interest as the output signal. Therefore, the expression vector of all proteins in STPs and the output signal of a chosen protein *Z* of interest in the STPs can be represented by

$$\left\{ \begin{array}{l} x={\left(I_{N}-\overset{\wedge }{A}\right)}^{-1}\overset{\wedge }{D}u+{\left(I_{N}-\overset{\wedge }{A}\right)}^{-1}\left[\overset{\wedge }{B}+E\right]\\ z=Gx\; \end{array}\right.$$ (15)

where *G* is used to choose the protein of interest and to measure its transductivity. If we are interested in the *i* target protein in the STPs and would like to measure its transductivity, all elements of the vector *G* are zero except for a single one at the *i^th^* component. For example, if we want to measure the transductivity of the first protein, *G* is set to be $\left[\begin{array}{cccc} 1 & 0 & \cdots & 0 \end{array}\right]$. Further, the output signal *Z* of a chosen protein of interest can be represented by

$$z=Gx=G{\left(I_{N}-\overset{\wedge }{A}\right)}^{-1}\overset{\wedge }{D}u+G{\left(I_{N}-\overset{\wedge }{A}\right)}^{-1}\left[\overset{\wedge }{B}+E\right]$$ (16)

where $G{\left(I_{N}-\overset{\wedge }{A}\right)}^{-1}\overset{\wedge }{D}$ denotes the transductive function from external signals *μ* to the chosen protein *Z*.

We first defined transductivity *ρ* from input signals *μ* to each protein at a leukemic subtype in the STP model as follows [13, 25]:

$$\begin{array}{l} \rho (\text{protein},\text{ subtype})=\underset{u\in l_{2}}{\text{sup}}\frac{{\left\|z\right\|}_{2}}{{\left\|u\right\|}_{2}}={\left\|G{\left(I_{N}-\overset{\wedge }{A}\right)}^{-1}\overset{\wedge }{D}\right\|}_{2}\\ \;=\sigma_{\max}\left(G{\left(I_{N}-\overset{\wedge }{A}\right)}^{-1}\overset{\wedge }{D}\right) \end{array}$$ (17)

where *u* and *z* denotes the input signals and the output signal of the STPs. For an input signal vector $u={\left[\begin{array}{ccc} u_{1} & \cdots & u_{N} \end{array}\right]}^{T}$, the *l*_2_ norm for *μ* is defined by ${\left\|u\right\|}_{2}=\sqrt{u_{1}{}^{2}+\cdots +u_{N}{}^{2}}$. *l*_2_ denotes the set of all bounded signals with ${\left\|u\right\|}_{2}<\infty$. The *l*_2_ induced matrix norm in (17) denotes the system gain from input signal *μ* to the output signal *Z* and is defined as $\rho =\sup_{u\in l_{2}}{\left\|z\right\|}_{2}/{\left\|u\right\|}_{2}$. That means the transductivity *ρ* in (Eqn. 17) is derived from the worst-case output/input ratio ${\left\|z\right\|}_{2}/{\left\|u\right\|}_{2}$ for all possible bounded inputs from the system gain perspective. $\sigma_{\max}\left(G{\left(I_{N}-\hat{A}\right)}^{-1}\hat{D}\right)$ denotes the largest singular value of $\left(G{\left(I_{N}-\hat{A}\right)}^{-1}\hat{D}\right)$. The physical meaning of transductivity is the maximal energy ratio of the output signal to all possible bounded input signals. The reason for using the maximal energy ratio of the output signals to all ligand input signals is that the ligand signals may vary with environmental conditions, causing the energy ratio to change. Thus, transductivity should be evaluated according to the maximal effect of all possible external ligand signals on the output levels of expression. It exhibits a greater dependency on the systematic characteristics of the steady system in accordance with system gain [25].

In this study, the transductivity of a protein in the STPs implies its abilities to transduce signals from all possible external signals to the downstream interactive proteins. We use the transductivity to discuss how much a protein transduces signals in the STPs in response to all ligand input signals and further orchestrate the program to associate with the cellular functions about apoptosis, cell survival, cell cycle progression, differentiation, cell proliferation and detoxification of ROS. To compare the transductivities of a protein in leukemic subtypes with normal type in the STPs, the transductivity sensitivities of different leukemic subtypes are also measured to investigate the transductivity changes of these proteins in the STPs during the leukemogenic process. If the transductivity of the protein p53 in the STPs of the MDS subtype is denoted by *ρ*(p53,MDS), the transductivity sensitivity Δ*ρ* of a protein between each leukemic subtype and normal type was denoted as follows:

$$\Delta \rho (\text{protein,subtype})=\frac{\rho (\text{protein,subtype})-\rho (\text{protein,non-leukemia})}{\rho (\text{protein,subtype})+\rho (\text{protein,non-leukemia})}$$ (18)

Let us illustrate how to use the measurements of original transductivity *ρ* and transductivity sensitivity Δ*ρ*. The transductivity *ρ* of a specific TF in the STPs at each subtype implies its ability to transduce signals from all external signals to the downstream genes at each subtype cells. If *ρ* of the specific TF is larger than 1, then all external ligand signals will be amplified through the TF. That means with a small amount of ligands, the TF can easily transduce the information to the downstream genes for transcription regulation. If the *ρ* of the specific TF is less than 1, then all external signals will be attenuated or buffered through the TF. That means the TF will require a more amount of ligands to transduce the signal to the downstream gene. Also, the TF with larger *ρ* can transduce more signals to respond to all external signals. Based on Δ*ρ*, we can realize whether TF is more sensitive to all external signals than normal type. If the Δ*ρ* of specific TF is sufficient positive/negative around ±1, then the TF is more sensitive in transductivity with different tendencies such as gain/loss of functions to leukemogenesis. Further, the resultant signal transductive programs of cellular functions about apoptosis, cell cycle progression and differentiation help us get insight into the pathophysiological phenotypes in leukemic subtypes. Consequently, investigating Δ*ρ* of specific TF between leukemic subtype and normal type reveals how much the TF functions in the leukemic subtypes change in response to all external signals through the crosstalk in coupling STPs.

Similarly, we also compared the basal level and estimation error of a protein in leukemic subtypes with normal type in the STPs. Because epigenetic regulation, such as DNA methylation and microRNA regulation, of genes are associated with its transcription basal level [18, 20, 21, 79, 80], the change of a basal level from normal type to leukemic subtypes implicates the occurrence of epigenetic regulation. Also, the change of an estimation error from normal type to leukemic subtypes implicates the impact of pathways other than those in Supplementary Figure 1. Therefore, we respectively defined the error sensitivity Δ*E*, and basal sensitivity Δ*B* of a protein between each leukemic subtype and normal type as follows:

$$\Delta E(\text{protein,subtype})=\frac{E(\text{protein,subtype})-E(\text{protein,non-leukemia})}{E(\text{protein,subtype})+E(\text{protein,non-leukemia})}$$ (19)

$$\Delta B(\text{protein,subtype})=\frac{\hat{B}(\text{protein,subtype})-\hat{B}(\text{protein,non-leukemia})}{\hat{B}(\text{protein,subtype})+\hat{B}(\text{protein,non-leukemia})}$$ (20)

where *E*(protein,subtype), and $\overset{\wedge }{B}$ (protein,subtype) respectively denote the estimation error, and the basal level of a protein in a leukemic subtype in (Eqn. 14).

Furthermore, we proposed a measure of correlation sensitivity to compare the results of the transductivity sensitivities in this study with a general assumption that the induced expression level of a TF in the downstream of the coupled signaling pathways is positively or negatively correlated with the ligand protein expression level of the pathways [30–32]. We defined the correlation, *C_r_*, between a protein and all input ligand signals *u* at a leukemic subtype in the STP model based on Pearson's correlation as follows [13, 25].

$$\begin{array}{l} Cr(\text{protein, leukemic subtype})=\\ \|[\begin{array}{cc} \frac{\sum_{k=1}^{K}\left\{x_{i}(k)-\sum_{k=1}^{K}\left[x_{i}(k)\right]/K\right\}\left\{u_{1}(k)-\sum_{k=1}^{K}\left[u_{1}(k)\right]/K\right\}}{\sqrt{\sum_{k=1}^{K}{\left\{x_{i}(k)-\sum_{k=1}^{K}\left[x_{i}(k)\right]/K\right\}}^{2}}\sqrt{\sum_{k=1}^{K}{\left\{u_{1}(k)-\sum_{k=1}^{K}\left[u_{1}(k)\right]/K\right\}}^{2}}} & \cdots \end{array}\\ {\frac{\sum_{k=1}^{K}\left\{x_{i}(k)-\sum_{k=1}^{K}\left[x_{i}(k)\right]/K\right\}\left\{u_{N}(k)-\sum_{k=1}^{K}\left[u_{N}(k)\right]/K\right\}}{\sqrt{\sum_{k=1}^{K}{\left\{x_{i}(k)-\sum_{k=1}^{K}\left[x_{i}(k)\right]/K\right\}}^{2}}\sqrt{\sum_{k=1}^{K}{\left\{u_{N}(k)-\sum_{k=1}^{K}\left[u_{N}(k)\right]/K\right\}}^{2}}}]\|}_{2} \end{array}$$ (21)

The correlation sensitivity Δ*Cr* of a protein between each leukemic subtype and normal type was denoted as follows:

$$\Delta \rho (\text{protein,subtype})=\frac{\rho (\text{protein,subtype})-\rho (\text{protein,non-leukemia})}{\rho (\text{protein,subtype})+\rho (\text{protein,non-leukemia})}$$ (22)

#### Drug design based on transductivity sensitivity, using drug response genome-wide microarray data

Because drug therapy induces a genome-wide response, we applied transductivity sensitivity to drug design for treating patients instead of gene expressions. In order to design drugs for treating patients with a leukemic subtype based on transductivity sensitivity, we considered the Connectivity Map (or CMap) database, which contains the genome-wide microarray data in response to 1327 drugs in five cell lines, MCF7, HL60, ssMCF7, PC3, and SKMEL5. Because larger sample sizes generally lead to increased precision, we used all data in five cell lines. We used microarray data with drugs (≤10^-8^ (M)) as the non-treated conditions (*N* = 69). Using drug response genome-wide microarray data, we calculated the transductivity sensitivities of 28 TFs (Supplementary Figure 3) for each drug, i.e. Δ*ρ*(*TF_i_*, *drug_j_*) for the *i*^th^ TF and *j*^th^ drug. We then defined a criteria to identify the best drug for treating patients with a leukemic subtype based on the transductivity sensitivities of 28 TFs from microarray data (Figure 4B) and from drug response microarray data in Connectivity Map (CMap) as follows.

$$E_{j}(subtype)=\sum_{i=1}^{28}-\Delta \rho (TF_{i},drug_{j})\Delta \rho (TF_{i},subtype)$$

$$Drug(subtype)=\underset{drug_{j}}{\arg}\underset{j}{\max}E_{j}(subtype)$$ (23)

where *E_j_*(*subtype*) denotes the effectiveness of the *j*^th^ drug for the patients with a leukemic subtype and *Drug*(*subtype*) represents the most effective drug for treating the patients with a leukemic subtype. We could identify the most effective drug, which has the transductivity sensitivities of 28 TFs inversely correlated with the transductivity sensitivities of 28 TFs a leukemic subtype.

## CONCLUSIONS

In this study, we applied system modeling and identification methods to estimate the interactive parameters of the STPs in normal type and MDS and AML subtypes. In order to identify the potential impact of genetic mutations, epigenetic alterations and the coupling of other pathways in MDS and AML subtypes, we defined the transductivity sensitivity, basal sensitivity, and error sensitivity of each protein in STPs, based on the identified interactive parameters, to suggest the dysregulated proteins in STPs leading to AML/MDS leukemogenesis and the potential leukemogenesis from MDS to AML. According to the results, we identified the effects of genetic mutations in TRAF2, MKK4, MDM2, JAK, PTEN, IKKs, ETO, JAK, CSLs, PSE2, Numb, CTBPs, and SMRT, the epigenetic regulations in ERK, AML1, and ERK, and the coupling of other pathways on c-Jun, and Axin during leukemogenesis. In order to test the reliability of the proposed results, we also applied the proposed methods to a validation dataset in MDS and AML subtypes. We suggested that the proposed results are reliable especially in 9 proliferation-related TFs CSLs, p300, CTBPs, AP-2α, STATs, c-Jun, β-catenin, LEF-1 and c-Fos, the anti-apoptosis-related TF Elk-1, the cell cycle-related TF pRb, 2 differentiation-related TFs ETO and PLZF, and 3 self-renewal-related TFs Smad2, Smad3 and Smad4. Finally, we applied the proposed methods to drug response genome-wide microarray data for potential drug repurposing. We proposed the drugs, decitabine, genistein, and monorden, for preventing the progression of AML and the drugs, decitabine, thalidomide, and geldanamycin, for preventing the progression of MDS.

Although the number of sample data was important for the proposed method, recent advances in high-throughput technologies will increase genome-wide expression sample data from different patients. The proposed transductivity of STPs and network robustness of GRNs from patient microarray sample data based on systems theory are the efficient tool for analyzing carcinogenesis and treating patients with leukemia.

## SUPPLEMENTARY MATERIALS FIGURES AND TABLES











## Abbreviations

17-AAG: 17-N-allylamino-17-demethoxy geldanamycin
AML: acute myeloid leukemia
AML+nok/abn: AML with normal karyotype plus other abnormalities
CMap: Connectivity Map
FDA: The U.S. Food and Drug Administration
FLT3: FMS-like tyrosine kinase 3
GRNs: gene regulatory networks
Hes1: Hairy-Enhancer of Split 1
HMGA1: High mobility group A1
HSCs: hematopoietic stem cells
HSP90: heat shock protein 90
ICN: intracellular Notch
KEGG: Kyoto Encyclopedia of Genes and Genomes
LICs: leukemia-initiating cells
LSCs: leukemic stem cells
MDS: myelodysplastic syndrome
PPIs: protein–protein interactions
STAT: signal transducer and activator of transcriptions
T-LGL: T cell large granular lymphocytes
TF: transcription factor
TGFβ: transforming growth factor beta
TNF: tumor necrosis factor, transductivity, transduction ability
STPs: signal transduction pathways
