# Supplementary material for: Changes of signal transductivity and robustness of gene regulatory network in the carcinogenesis of leukemic subtypes via microarray sample data
Source: Oncotarget. 2018 May 4;9(34):23636–60. doi: 10.18632/oncotarget.25318 (PMC5955113; doi:10.18632/oncotarget.25318)
Supplement: Supplementary file 1 [file oncotarget-09-23636-s001.pdf]

# Changes of signal transductivity and robustness of gene regulatory network in the carcinogenesis of leukemic subtypes via microarray sample data

## SUPPLEMENTARY MATERIALS

Suppose the regulatory matrix $\tilde{C}$ suffers from the following intrinsic parameter variation $\Delta C$ due to genetic variation, splicing and other uncertainties as follows:

In this situation, (5) could be changed as

$$y=(\hat{C}+\Delta C)y+\hat{H}+W \qquad (S1)$$

$$(I_M-\hat{C}-\Delta C)y=\hat{H}+W \qquad (S2)$$

If the GRN can tolerate $\Delta C$, then the inverse of perturbative regulatory matrix $(I_M-\hat{C}-\Delta C)$ exists and the gene expression vector $y$ of perturbative GRN can be represented by

$$y=(I_M-\hat{C}-\Delta C)^{-1}[\hat{H}+W] \qquad (S3)$$

Let (S2) be modified below

$$(I_M-\hat{C})(I_M-(I_M-C)^{-1}\Delta C)y=\hat{H}+W \qquad (S4)$$

If the following robustness condition holds [1]

$$\left\|(I_M-\hat{C})^{-1}\right\|_2\|\Delta C\|_2<1 \quad \text{or} \quad \left\|\tilde{C}^{-1}\right\|_2\|\Delta C\|_2<1 \qquad (S5)$$

then $I_M-(I_M-\hat{C})^{-1}\Delta C$ is always nonsingular, i.e. the inverse of $(I_M-\hat{C}-\Delta C)$ always exists. The physical meaning is that the perturbative GRN can tolerate the variation and the steady state of model (S3) exists. According to [2], it can be shown that

$$\left\|(I_M-\hat{C})^{-1}\right\|_2=\frac{1}{\sigma_{\min}(I_M-\hat{C})} \quad \text{or} \quad \left\|\tilde{C}^{-1}\right\|_2=\frac{1}{\sigma_{\min}(\tilde{C})} \qquad (S6)$$

where $\sigma_{\min}(\tilde{C})=\min_j \sigma_j(\tilde{C})$, i.e. the minimum singular value of the transduction regulatory matrix $\tilde{C}$. Therefore, from (S5) and (S6), the robustness condition can be rewritten as

$$\|\Delta C\|_2<\sigma_{\min}(I_M-\hat{C}) \quad \text{or} \quad \|\Delta C\|_2<\sigma_{\min}(\tilde{C})$$

i.e. The network robustness $\xi$ is equal to the largest tolerance of perturbation $\sigma_{\min}(I_M-\hat{C})$ or $\sigma_{\min}(\tilde{C})$ Q.E.D.

## REFERENCES


1. Noble B, Daniel JW. Applied linear algebra. (Englewood Cliffs, N.J.: Prentice Hall). 1977.
2. Press WH and Numerical Recipes Software (Firm). Numerical recipes in C the art of scientific computing. (Cambridge, England ; New York, NY USA: Cambridge University Press). 1992.
3. Etet PFS, Vecchio L and Kamdje AHN. Signaling pathways in chronic myeloid leukemia and leukemic stem cell maintenance: Key role of stromal microenvironment. Cellular Signalling. 2012; 24:883–1888.
4. Martelli AM, Evangelisti C, Chappell W, Abrams SL, Basecke J, Stivala F, Donia M, Fagone P, Nicoletti F, Libra M, Ruvolo V, Ruvolo P, Kempf CR, Steelman LS and McCubrey JA. Targeting the translational apparatus to improve leukemia therapy: roles of the PI3K/PTEN/Akt/mTOR pathway. Leukemia. 2011; 25:1064–1079.
5. Rizo A, Vellenga E, de Haan G and Schuringa JJ. Signaling pathways in self-renewing hematopoietic and leukemic stem cells: do all stem cells need a niche? Human Molecular Genetics. 2006; 15:R210–R219.
6. Sands WA, Copland M and Wheadon H. Targeting self-renewal pathways in myeloid malignancies. Cell Communication and Signaling. 2013; 11.
7. Steelman LS, Abrams SL, Whelan J, Bertrand FE, Ludwig DE, Basecke J, Libra M, Stivala F, Milella M, Tafuri A, Lunghi P, Bonati A, Martelli AM and McCubrey JA. Contributions of the Raf/MEK/ERK, PI3K/PTEN/Akt/mTOR and Jak/STAT pathways to leukemia. Leukemia. 2008; 22:686–707.
8. Steelman LS, Franklin RA, Abrams SL, Chappell W, Kempf CR, Basecke J, Stivala F, Donia M, Fagone P, Nicoletti F, Libra M, Ruvolo P, Ruvolo V, Evangelisti C, Martelli AM and McCubrey JA. Roles of the Ras/Raf/MEK/ERK pathway in leukemia therapy. Leukemia. 2011; 25:1080–1094.
9. Takahashi S. Downstream molecular pathways of FLT3 in the pathogenesis of acute myeloid leukemia: biology and therapeutic implications. Journal of Hematology & Oncology. 2011; 4.
10. Kanehisa M and Goto S. KEGG: Kyoto Encyclopedia of Genes and Genomes. Nucleic Acids Research. 2000; 28:27–30.
11. Kanehisa M, Goto S, Sato Y, Kawashima M, Furumichi M and Tanabe M. Data, information, knowledge and principle: back to metabolism in KEGG. Nucleic Acids Research. 2014; 42:D199–D205.

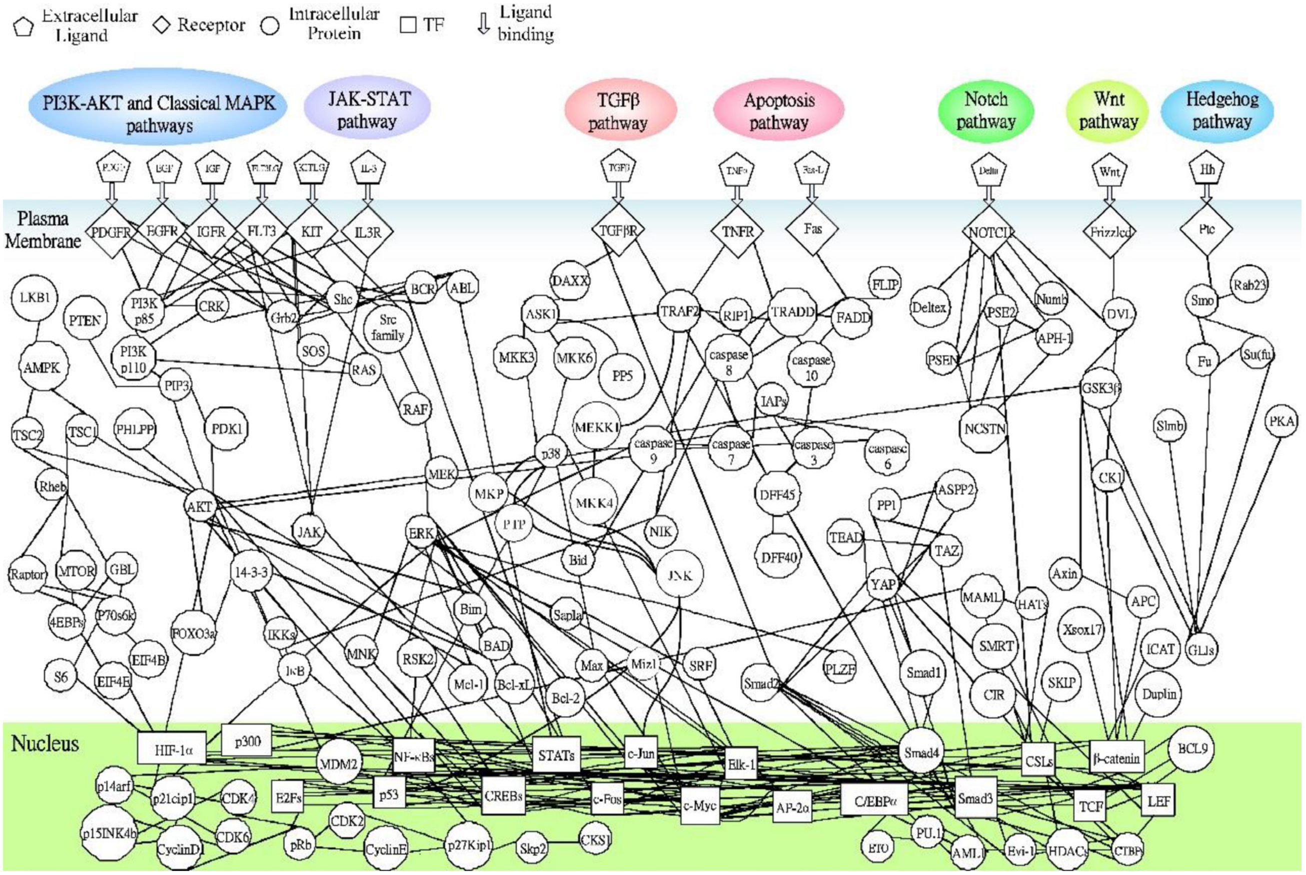


**Supplementary Figure 1: Integrated cellular interactions network of 8 leukemogenesis-related coupling STPs.** The network comprises 159 proteins and 28 TFs. 18 TFs at the downstream of 8 STPs are determined by gene regulations from TRANSFAC database and are viewed as 18 genes of TFs of GRN in Supplementary Figure 2. The solid lines between TFs in nucleus denote GRN as shown in Supplementary Figure 2 while the others in cytoplasm denote PPIs. Other graphical symbols are defined and shown on top of this figure. The leukemogenesis-related STPs comprising of PI3K-AKT, classical MAPK, JAK-STAT, TGFβ, Apoptosis, Notch, Hedgehog and the canonical Wnt pathways are constructed by the literatures [3–9] and KEGG database [10, 11]. Different STPs are coupled by the crosstalk between pathways. As the genetic mutations or dysregulations occur at TFs or upstream proteins in the STPs, the accumulated influence from mutations may lead to dysfunction of TFs through the propagation and crosstalk in coupling STPs.

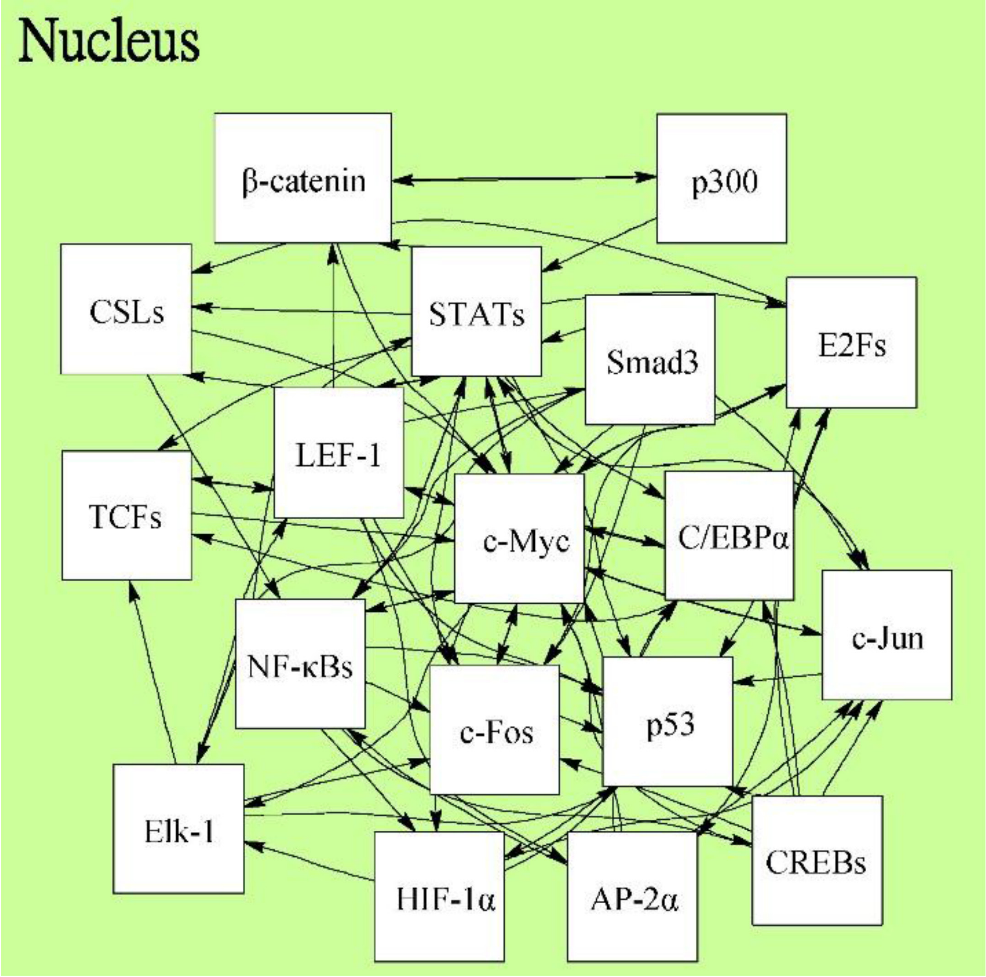


**Supplementary Figure 2: Multiple loops of GRN, extracted from the GRN with mutual regulations in nucleus of Supplementary Figure 1, associated with leukemogenesis-related pathophysiological phenotypes.** This is the downstream part of Supplementary Figure 1. Each TF regulates other genes and acts as a target gene regulated by other TFs.

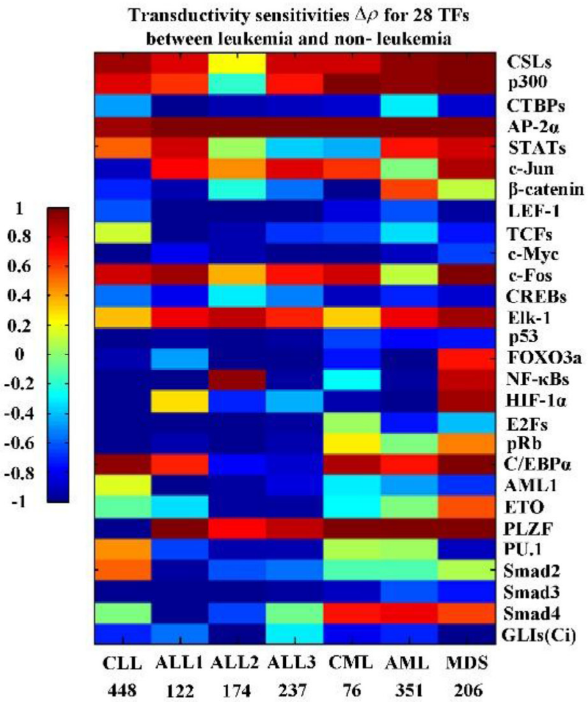


The transductivities $\rho$ for 28 TFs in leukemia and non-leukemia

| CLL | ALL1 | ALL2 | ALL3 | CML | AML | MDS | Non-Leukemia |
|---|---|---|---|---|---|---|---|
| 0.1302 | 0.0486 | 0.0096 | 0.0606 | 0.0587 | 0.2267 | 0.4392 | 0.0059 |
| 0.0017 | 0.0008 | 0.0001 | 0.0011 | 0.1965 | 0.0065 | 0.0173 | 0.0002 |
| 0.0882 | 0.0006 | 0.0101 | 0.0118 | 0.0158 | 0.1233 | 0.0184 | 0.2262 |
| 0.0000 | 0.0028 | 0.0003 | 0.0002 | 0.0017 | 0.0016 | 0.0150 | 0.0000 |
| 0.5083 | 1.5867 | 0.1564 | 0.0667 | 0.0596 | 0.8370 | 1.5071 | 0.1456 |
| 0.0000 | 0.0006 | 0.0003 | 0.0009 | 0.0004 | 0.0001 | 0.0016 | 0.0001 |
| 0.0029 | 0.0006 | 0.0105 | 0.0044 | 0.0001 | 0.0659 | 0.0197 | 0.0158 |
| 0.0543 | 0.0000 | 0.0029 | 0.0005 | 0.0194 | 0.0537 | 0.0037 | 0.2228 |
| 0.0250 | 0.0002 | 0.0007 | 0.0037 | 0.0040 | 0.0096 | 0.0029 | 0.0192 |
| 0.0002 | 0.0020 | 0.0007 | 0.0000 | 0.0001 | 0.0009 | 0.0035 | 0.0169 |
| 0.0000 | 0.0001 | 0.0000 | 0.0000 | 0.0000 | 0.0000 | 0.0005 | 0.0000 |
| 0.0059 | 0.0024 | 0.0106 | 0.0061 | 0.0012 | 0.0033 | 0.0015 | 0.0198 |
| 0.0009 | 0.0034 | 0.0056 | 0.0021 | 0.0009 | 0.0034 | 0.0124 | 0.0004 |
| 0.0001 | 0.0003 | 0.0002 | 0.0003 | 0.0041 | 0.0026 | 0.0028 | 0.0187 |
| 0.0002 | 0.0016 | 0.0000 | 0.0000 | 0.0006 | 0.0000 | 0.0254 | 0.0042 |
| 0.0000 | 0.0000 | 0.0807 | 0.0001 | 0.0014 | 0.0000 | 0.0360 | 0.0025 |
| 0.0000 | 0.0000 | 0.0000 | 0.0000 | 0.0000 | 0.0000 | 0.0000 | 0.0000 |
| 0.0000 | 0.0000 | 0.0000 | 0.0000 | 0.0001 | 0.0000 | 0.0000 | 0.0001 |
| 0.0000 | 0.0000 | 0.0000 | 0.0000 | 0.0007 | 0.0004 | 0.0012 | 0.0004 |
| 0.0079 | 0.0012 | 0.0000 | 0.0000 | 0.0036 | 0.0014 | 0.0361 | 0.0002 |
| 0.0503 | 0.0001 | 0.0011 | 0.0030 | 0.0190 | 0.0134 | 0.0068 | 0.0347 |
| 0.0054 | 0.0031 | 0.0001 | 0.0001 | 0.0036 | 0.0059 | 0.0235 | 0.0062 |
| 0.0000 | 0.0022 | 0.0001 | 0.0002 | 0.0035 | 0.0012 | 0.0009 | 0.0000 |
| 0.0110 | 0.0009 | 0.0002 | 0.0002 | 0.0045 | 0.0045 | 0.0003 | 0.0040 |
| 0.1293 | 0.0006 | 0.0085 | 0.0105 | 0.0297 | 0.0297 | 0.0428 | 0.0362 |
| 0.0063 | 0.0000 | 0.0022 | 0.0079 | 0.0287 | 0.1089 | 0.0696 | 0.4576 |
| 0.0143 | 0.0000 | 0.0033 | 0.0130 | 0.0843 | 0.1090 | 0.0608 | 0.0146 |
| 0.0142 | 0.0228 | 0.0006 | 0.0428 | 0.0084 | 0.0132 | 0.0001 | 0.0804 |

**Supplementary Figure 3: Transductivity sensitivities (left panel) and transductivities (right panel) of 28 TFs at seven leukemic cells and one normal subtype.**

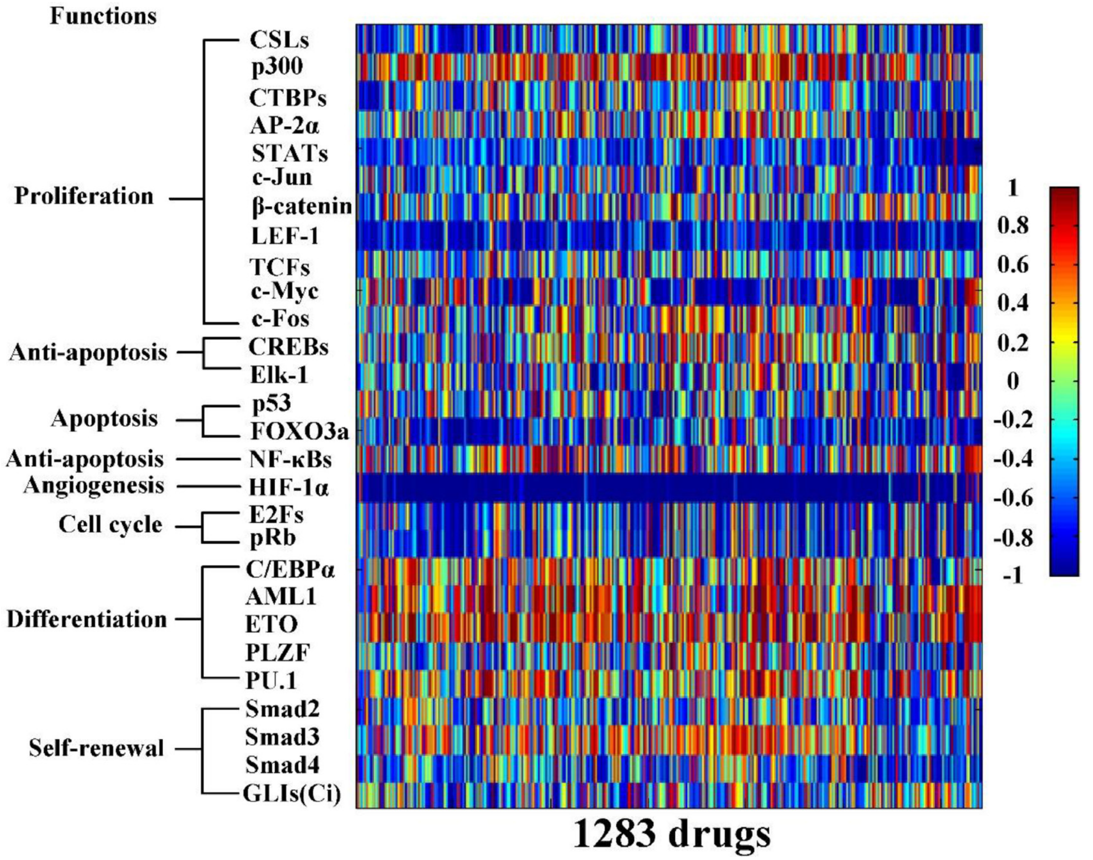


**Supplementary Figure 4: The transductivity sensitivities of 28 TFs for 1327 drugs.** Because 44 drugs have no sample data with drugs (>$10^{-8}$ (M)), which can not be used to calculate transductivity sensitivity, the result shows the transductivity sensitivities of 28 TFs for 1283 drugs.

**Supplementary Table 1: Transductivities of proteins with 159 proteins at leukemic and normal subtypes based on (17).** See Supplementary_Table_1

**Supplementary Table 2: The fold changes and variances of 159 proteins in the coupling STPs (Supplementary Figure 1).** See Supplementary_Table_2

**Supplementary Table 3: The proteins belong to the 159 groups of proteins in the model of coupling STPs.** See Supplementary_Table_3

**Supplementary Table 4: The full table of effectiveness of drugs for treating patients with AML/MDS.** See Supplementary_ Table_4
