# Supplementary material for: Changes of signal transductivity and robustness of gene regulatory network in the carcinogenesis of leukemic subtypes via microarray sample data
Source: Oncotarget. 2018 May 4;9(34):23636–60. doi: 10.18632/oncotarget.25318 (PMC5955113; doi:10.18632/oncotarget.25318)
Supplement: Supplementary file 2 [file oncotarget-09-23636-s002.docx]

**Supplementary Table S1: Transductivities of proteins with 159 proteins at leukemic and normal subtypes based on (17)**

| Groups  of proteins | CLL | ALL1 | ALL2 | ALL3 | CML | AML | MDS | Normal |
| --- | --- | --- | --- | --- | --- | --- | --- | --- |
| 14-3-3 | 0.000102 | 0.000671 | 0.000006 | 0.000002 | 0.000071 | 0.000042 | 0.001669 | 0.008523 |
| 4EBPs | 0.000000 | 0.000001 | 0.000019 | 0.000030 | 0.000000 | 0.000001 | 0.000575 | 0.000004 |
| ABL | 0.037431 | 0.233491 | 0.040847 | 0.010092 | 0.018018 | 0.167588 | 0.502623 | 0.022522 |
| AKT | 0.000491 | 0.008969 | 0.000095 | 0.003453 | 0.004010 | 0.000789 | 0.042160 | 0.014418 |
| AML1 | 0.050260 | 0.000087 | 0.001116 | 0.003007 | 0.018994 | 0.013406 | 0.006752 | 0.034703 |
| AMPK | 0.000148 | 0.000145 | 0.000027 | 0.002265 | 0.000015 | 0.001080 | 0.002582 | 0.004177 |
| AP-2α | 0.000014 | 0.002812 | 0.000257 | 0.000202 | 0.001665 | 0.001619 | 0.014974 | 0.000001 |
| APC | 0.001028 | 0.001223 | 0.000882 | 0.001907 | 0.003794 | 0.073042 | 0.005881 | 0.000719 |
| APH-1 | 0.001530 | 0.003634 | 0.003075 | 0.010817 | 0.060200 | 0.003274 | 0.020087 | 0.013689 |
| ASK1 | 0.000432 | 0.006808 | 0.008814 | 0.000660 | 0.037741 | 0.002681 | 0.138593 | 0.322005 |
| ASPP2 | 0.006523 | 0.000002 | 0.000259 | 0.003632 | 0.001610 | 0.069634 | 0.000009 | 0.300126 |
| Axin | 0.005217 | 0.014890 | 0.047895 | 0.030981 | 0.027329 | 0.174275 | 0.003679 | 0.012873 |
| β-catenin | 0.002854 | 0.000572 | 0.010549 | 0.004442 | 0.000066 | 0.065852 | 0.019664 | 0.015767 |
| BAD | 0.000691 | 0.003899 | 0.000331 | 0.000177 | 0.000584 | 0.000838 | 0.005371 | 0.001786 |
| BCL9-2 | 0.067103 | 0.000131 | 0.000217 | 0.000016 | 0.001699 | 0.013390 | 0.000020 | 0.000233 |
| BCR | 0.879035 | 4.930696 | 0.238128 | 0.179572 | 0.051558 | 0.758114 | 1.569014 | 0.148186 |
| Bcl-2 | 0.000002 | 0.000723 | 0.000061 | 0.000008 | 0.000027 | 0.000018 | 0.000351 | 0.000339 |
| Bcl-XL | 0.000867 | 0.009349 | 0.000000 | 0.000537 | 0.000024 | 0.000121 | 0.000021 | 0.000051 |
| Bid | 0.000003 | 0.000428 | 0.002395 | 0.000453 | 0.001590 | 0.000030 | 0.000034 | 0.000082 |
| Bim | 0.000033 | 0.002334 | 0.000122 | 0.001965 | 0.005176 | 0.001662 | 0.024815 | 0.012987 |
| C/EBP-α | 0.007917 | 0.001212 | 0.000032 | 0.000016 | 0.003647 | 0.001410 | 0.036069 | 0.000243 |
| p300 | 0.001653 | 0.000831 | 0.000135 | 0.001101 | 0.196479 | 0.006481 | 0.017297 | 0.000188 |
| CDK2 | 0.000001 | 0.000033 | 0.000000 | 0.000118 | 0.000129 | 0.000040 | 0.003965 | 0.000048 |
| CDK4 | 0.000044 | 0.000034 | 0.000000 | 0.000005 | 0.000112 | 0.003470 | 0.000062 | 0.002186 |
| CDK6 | 0.000000 | 0.000010 | 0.000000 | 0.000044 | 0.000009 | 0.000678 | 0.000064 | 0.001541 |
| CIR | 0.024439 | 0.002919 | 0.000785 | 0.008695 | 0.000265 | 0.042544 | 0.052269 | 0.001723 |
| CK1 | 1.265391 | 0.063612 | 0.011513 | 0.079748 | 0.026565 | 0.035109 | 0.008319 | 0.010642 |
| CKS1 | 0.000000 | 0.000106 | 0.000000 | 0.000028 | 0.000006 | 0.000013 | 0.000063 | 0.000080 |
| CREBs | 0.005935 | 0.002366 | 0.010610 | 0.006150 | 0.001200 | 0.003265 | 0.001514 | 0.019839 |
| CRK | 0.723973 | 2.169442 | 1.488356 | 0.298289 | 0.475125 | 0.505419 | 0.256752 | 0.499044 |
| CSLs | 0.130165 | 0.048591 | 0.009642 | 0.060622 | 0.058723 | 0.226695 | 0.439170 | 0.005936 |
| Ci | 0.014198 | 0.022790 | 0.000609 | 0.042790 | 0.008371 | 0.013218 | 0.000104 | 0.080351 |
| CtBP | 0.088177 | 0.000574 | 0.010110 | 0.011824 | 0.015843 | 0.123317 | 0.018404 | 0.226211 |
| CyclinD1 | 0.000075 | 0.000054 | 0.000002 | 0.000052 | 0.009601 | 0.000072 | 0.000028 | 0.000081 |
| CyclinE | 0.000004 | 0.000001 | 0.000000 | 0.000050 | 0.000295 | 0.000003 | 0.002379 | 0.000089 |
| DAXX | 0.000336 | 0.008811 | 0.050999 | 0.002878 | 0.079428 | 0.004153 | 0.542258 | 0.906262 |
| DFF40 | 0.000000 | 0.000001 | 0.000049 | 0.000689 | 0.000000 | 0.000011 | 0.000007 | 0.027585 |
| DFF45 | 0.000000 | 0.000024 | 0.001070 | 0.017331 | 0.000064 | 0.001357 | 0.000212 | 0.125470 |
| DVL | 0.841484 | 0.247666 | 0.357476 | 0.400937 | 0.535476 | 0.367331 | 0.073907 | 0.338777 |
| Deltex | 0.004073 | 0.000015 | 0.000311 | 0.001133 | 0.016084 | 0.010305 | 0.050137 | 0.159650 |
| Duplin | 0.000026 | 0.000111 | 0.000796 | 0.000751 | 0.000003 | 0.000717 | 0.000417 | 0.000189 |
| E2Fs | 0.000000 | 0.000001 | 0.000000 | 0.000002 | 0.000123 | 0.000016 | 0.000048 | 0.000111 |
| EIF4B | 0.000000 | 0.000000 | 0.000000 | 0.000000 | 0.000000 | 0.000000 | 0.000000 | 0.000001 |
| EIF4E | 0.000000 | 0.000001 | 0.000003 | 0.000007 | 0.000000 | 0.000000 | 0.000086 | 0.000000 |
| ERK | 0.000053 | 0.005050 | 0.000647 | 0.000688 | 0.004261 | 0.005943 | 0.017666 | 0.000027 |
| ETO | 0.005413 | 0.003117 | 0.000121 | 0.000110 | 0.003579 | 0.005942 | 0.023505 | 0.006240 |
| EVI1 | 0.029607 | 0.000257 | 0.000985 | 0.000622 | 0.005072 | 0.018376 | 0.005869 | 0.156440 |
| Elk-1 | 0.000928 | 0.003400 | 0.005615 | 0.002085 | 0.000853 | 0.003398 | 0.012427 | 0.000424 |
| FADD | 0.010778 | 0.116530 | 0.200242 | 0.035269 | 0.004253 | 0.005379 | 0.003916 | 0.090097 |
| FLIP | 0.000066 | 0.127042 | 0.076129 | 0.019101 | 0.000063 | 0.000103 | 0.000013 | 0.000003 |
| FOXO3a | 0.000155 | 0.001591 | 0.000003 | 0.000017 | 0.000633 | 0.000018 | 0.025403 | 0.004194 |
| c-Fos | 0.000032 | 0.000080 | 0.000007 | 0.000018 | 0.000035 | 0.000004 | 0.000512 | 0.000003 |
| Fu | 0.001207 | 0.001547 | 0.000086 | 0.090955 | 0.023308 | 0.005309 | 0.000263 | 0.050055 |
| GBL | 0.000001 | 0.000011 | 0.000018 | 0.000008 | 0.000000 | 0.000001 | 0.000548 | 0.000073 |
| GSK3β | 0.121337 | 0.065846 | 0.121616 | 0.146802 | 0.326051 | 0.294827 | 0.059375 | 0.038197 |
| Grb2 | 0.565843 | 6.108620 | 0.156013 | 0.252738 | 0.071417 | 0.540819 | 0.181267 | 0.013417 |
| HATs | 0.000960 | 0.002203 | 0.000392 | 0.000071 | 0.002125 | 0.041400 | 0.011125 | 0.000091 |
| HDAC | 0.010011 | 0.000986 | 0.001111 | 0.049377 | 0.011533 | 0.131872 | 0.038134 | 0.387493 |
| HIF-1α | 0.000000 | 0.000000 | 0.000000 | 0.000000 | 0.000000 | 0.000000 | 0.000003 | 0.000000 |
| IAPs | 0.000403 | 0.000693 | 0.015233 | 0.022561 | 0.043356 | 0.001101 | 0.001386 | 0.024265 |
| ICAT | 0.000001 | 0.001571 | 0.000152 | 0.007359 | 0.000586 | 0.000344 | 0.005230 | 0.000004 |
| IκB | 0.000017 | 0.000037 | 0.075281 | 0.000011 | 0.002316 | 0.000080 | 0.055336 | 0.001957 |
| IKKs | 0.000113 | 0.007020 | 0.035973 | 0.001885 | 0.002657 | 0.000228 | 0.053726 | 0.001056 |
| JAK | 1.548188 | 0.922697 | 0.121715 | 0.399628 | 0.066031 | 0.314527 | 0.050690 | 0.153229 |
| JNK | 0.000005 | 0.000574 | 0.000193 | 0.000437 | 0.000735 | 0.001437 | 0.000674 | 0.000018 |
| c-Jun | 0.000005 | 0.000632 | 0.000268 | 0.000869 | 0.000445 | 0.000098 | 0.001598 | 0.000101 |
| LEF-1 | 0.054348 | 0.000033 | 0.002926 | 0.000541 | 0.019368 | 0.053655 | 0.003653 | 0.222793 |
| LKB1 | 0.000001 | 0.000067 | 0.000000 | 0.000001 | 0.000000 | 0.000017 | 0.000084 | 0.000068 |
| MAML | 0.000185 | 0.009461 | 0.000414 | 0.002452 | 0.007074 | 0.040191 | 0.036724 | 0.000733 |
| MDM2 | 0.000014 | 0.002791 | 0.000076 | 0.000263 | 0.000767 | 0.002669 | 0.002987 | 0.008193 |
| MEK | 0.000542 | 0.006941 | 0.000698 | 0.000390 | 0.016278 | 0.000676 | 0.003906 | 0.012956 |
| MEKK1 | 0.001931 | 0.000271 | 0.004994 | 0.003175 | 0.000171 | 0.005119 | 0.043057 | 0.011719 |
| MKK3 | 0.000873 | 0.002160 | 0.000556 | 0.000193 | 0.003503 | 0.000017 | 0.000824 | 0.034615 |
| MKK4 | 0.000204 | 0.001230 | 0.000191 | 0.002055 | 0.000403 | 0.001279 | 0.007770 | 0.000997 |
| MKK6 | 0.001383 | 0.012051 | 0.003304 | 0.001289 | 0.001672 | 0.000809 | 0.022358 | 0.156667 |
| MKP | 0.000062 | 0.000484 | 0.000233 | 0.000090 | 0.000304 | 0.000349 | 0.001719 | 0.003462 |
| MNK | 0.000578 | 0.001046 | 0.010426 | 0.000327 | 0.001576 | 0.001209 | 0.006685 | 0.000649 |
| MTOR | 0.000004 | 0.000022 | 0.000003 | 0.000041 | 0.000000 | 0.000000 | 0.001231 | 0.000002 |
| Max | 0.001583 | 0.011131 | 0.001146 | 0.000914 | 0.004895 | 0.000193 | 0.037128 | 0.062693 |
| Mcl-1 | 0.000147 | 0.005258 | 0.000027 | 0.000340 | 0.004259 | 0.004894 | 0.019012 | 0.005258 |
| Miz1 | 0.000146 | 0.007191 | 0.000259 | 0.000150 | 0.001070 | 0.000025 | 0.001420 | 0.003228 |
| NCSTN | 0.002188 | 0.003652 | 0.044177 | 0.053890 | 0.091850 | 0.001168 | 0.152973 | 0.041603 |
| NF-κBs | 0.000000 | 0.000012 | 0.080668 | 0.000053 | 0.001419 | 0.000046 | 0.035957 | 0.002463 |
| NIK | 0.000119 | 0.026515 | 0.744194 | 0.015472 | 0.004301 | 0.000387 | 0.009811 | 0.018156 |
| Numb | 0.019708 | 0.020068 | 0.001258 | 0.002723 | 0.054836 | 0.003938 | 0.048760 | 0.098972 |
| P14arf | 0.000002 | 0.000048 | 0.000032 | 0.000074 | 0.000665 | 0.002465 | 0.001062 | 0.001761 |
| P70s6k | 0.000000 | 0.000026 | 0.000000 | 0.000007 | 0.000005 | 0.000000 | 0.000017 | 0.000011 |
| PDK1 | 0.000949 | 0.071151 | 0.010112 | 0.002724 | 0.846835 | 0.000203 | 0.000777 | 0.165719 |
| PHLPP | 0.000035 | 0.001853 | 0.000002 | 0.000187 | 0.000273 | 0.000074 | 0.004975 | 0.001579 |
| PI3Kc | 0.070932 | 0.104804 | 0.337220 | 0.003023 | 0.214040 | 0.017392 | 0.055889 | 0.132189 |
| PI3Kr | 1.416056 | 2.143882 | 1.728269 | 0.295222 | 0.305826 | 0.416272 | 0.202732 | 0.340374 |
| PIP3 | 0.046311 | 0.067021 | 0.284952 | 0.002800 | 0.798366 | 0.014669 | 0.001054 | 0.110268 |
| PKA | 0.008357 | 0.000372 | 0.000022 | 0.000177 | 0.004968 | 0.000530 | 0.000002 | 0.033193 |
| PLZF | 0.000000 | 0.002198 | 0.000078 | 0.000152 | 0.003498 | 0.001171 | 0.000862 | 0.000012 |
| PP1 | 0.000047 | 0.000006 | 0.001277 | 0.001655 | 0.000110 | 0.000112 | 0.000174 | 0.007159 |
| PP5 | 0.000134 | 0.001332 | 0.004592 | 0.000417 | 0.015516 | 0.001438 | 0.000927 | 0.010860 |
| PSE2 | 0.002491 | 0.000249 | 0.009501 | 0.020102 | 0.022204 | 0.001110 | 0.001603 | 0.012123 |
| PSEN | 0.014382 | 0.023918 | 0.038928 | 0.084063 | 0.025392 | 0.015173 | 0.300842 | 0.098669 |
| PTEN | 0.000002 | 0.000252 | 0.000829 | 0.000017 | 0.003230 | 0.000051 | 0.006898 | 0.000523 |
| PTP | 0.000530 | 0.001976 | 0.000200 | 0.000284 | 0.002221 | 0.000032 | 0.006962 | 0.007089 |
| PU.1 | 0.011019 | 0.000894 | 0.000157 | 0.000169 | 0.004535 | 0.004500 | 0.000250 | 0.003991 |
| Ptc | 0.002311 | 0.032519 | 0.196853 | 0.082068 | 0.011488 | 0.049062 | 0.041651 | 0.027108 |
| RAF | 0.007171 | 0.112480 | 0.074660 | 0.000223 | 0.187500 | 0.008852 | 0.054104 | 0.105288 |
| RAS | 0.050711 | 0.028560 | 0.403819 | 0.001142 | 0.264277 | 0.006297 | 0.111474 | 0.114430 |
| RIP1 | 0.000120 | 0.147924 | 0.183540 | 0.073168 | 0.013480 | 0.000021 | 0.002000 | 0.003918 |
| RSK2 | 0.007321 | 0.000051 | 0.002349 | 0.000467 | 0.007542 | 0.003518 | 0.008444 | 0.001831 |
| Rab23 | 0.000024 | 0.000011 | 0.000235 | 0.000014 | 0.001134 | 0.000020 | 0.038848 | 0.139884 |
| Raptor | 0.000000 | 0.000031 | 0.000000 | 0.000030 | 0.000028 | 0.000003 | 0.000123 | 0.000055 |
| Rb | 0.000001 | 0.000019 | 0.000000 | 0.000019 | 0.000749 | 0.000406 | 0.001235 | 0.000432 |
| Receptor-EGFR | 0.811536 | 1.610608 | 0.713856 | 0.028846 | 0.297388 | 0.353564 | 0.419742 | 0.144966 |
| Receptor-FLT3 | 0.897509 | 0.281751 | 1.783275 | 0.117995 | 1.055243 | 0.063110 | 0.272218 | 0.250974 |
| Receptor-Fas | 0.138635 | 0.045513 | 0.258172 | 0.036721 | 0.133485 | 0.136634 | 0.139805 | 0.128690 |
| Receptor-Frizzled | 0.928338 | 0.028545 | 0.055790 | 0.075829 | 0.102018 | 0.036230 | 1.624265 | 0.075017 |
| Receptor-IGFR | 0.268860 | 0.920833 | 0.064082 | 0.116372 | 0.077573 | 0.342586 | 0.343310 | 0.109904 |
| Receptor-IL3 | 0.805131 | 0.926366 | 0.217571 | 0.190977 | 0.251916 | 0.047667 | 0.062797 | 0.095064 |
| Receptor-KIT | 0.355932 | 3.796741 | 0.414417 | 0.201936 | 0.073312 | 0.083514 | 0.346425 | 0.716033 |
| Receptor-NOTCH | 0.073521 | 0.047120 | 0.026324 | 0.231030 | 0.352875 | 0.049787 | 0.309160 | 0.155600 |
| Receptor-PDGFR | 0.052495 | 0.216356 | 0.106691 | 0.201417 | 0.150895 | 0.082129 | 0.272482 | 0.441020 |
| Receptor-TGF-β | 0.021886 | 0.019920 | 0.080906 | 0.016204 | 0.207770 | 0.050038 | 0.331433 | 1.294768 |
| Receptor-TNF | 0.041882 | 0.713529 | 1.550441 | 0.036423 | 0.351007 | 0.024267 | 0.027904 | 0.017763 |
| Rheb | 0.000002 | 0.000079 | 0.000003 | 0.000150 | 0.000105 | 0.000022 | 0.001776 | 0.000151 |
| S6 | 0.000000 | 0.000001 | 0.000000 | 0.000000 | 0.000000 | 0.000000 | 0.000002 | 0.000003 |
| SKIP | 0.000701 | 0.005798 | 0.030809 | 0.180047 | 0.018299 | 0.003920 | 0.003701 | 0.000018 |
| SMRT | 0.000494 | 0.106774 | 0.000060 | 0.000369 | 0.000326 | 0.001258 | 0.006901 | 0.036675 |
| SOS | 0.024391 | 0.217935 | 0.029132 | 0.000421 | 0.043421 | 0.092787 | 0.049613 | 0.016719 |
| SRF | 0.001026 | 0.030595 | 0.048119 | 0.003221 | 0.000282 | 0.009646 | 0.004672 | 0.000316 |
| STATs | 0.508285 | 1.586684 | 0.156371 | 0.066684 | 0.059587 | 0.837029 | 1.507073 | 0.145600 |
| Sapla | 0.000026 | 0.031036 | 0.048856 | 0.000639 | 0.017031 | 0.000321 | 0.011553 | 0.000007 |
| Shc | 0.172125 | 0.094569 | 0.041528 | 0.058056 | 0.065005 | 0.418797 | 0.326294 | 0.639626 |
| Skp2 | 0.000000 | 0.000254 | 0.000000 | 0.000063 | 0.000010 | 0.000013 | 0.000084 | 0.000121 |
| Slmb | 0.000086 | 0.000240 | 0.000019 | 0.000228 | 0.000719 | 0.000323 | 0.000005 | 0.005890 |
| Smad1 | 0.000500 | 0.000005 | 0.000014 | 0.000373 | 0.071143 | 0.007926 | 0.002243 | 0.028395 |
| Smad2 | 0.129269 | 0.000617 | 0.008465 | 0.010533 | 0.029658 | 0.029741 | 0.042849 | 0.036215 |
| Smad3 | 0.006337 | 0.000013 | 0.002225 | 0.007948 | 0.028725 | 0.108902 | 0.069573 | 0.457591 |
| Smad4 | 0.014292 | 0.000031 | 0.003314 | 0.012994 | 0.084336 | 0.109012 | 0.060794 | 0.014573 |
| Smo | 0.017972 | 0.001165 | 0.002412 | 0.002076 | 0.000868 | 0.001591 | 0.039861 | 0.126241 |
| Src family kinase | 0.062031 | 0.350065 | 0.273064 | 0.004947 | 0.209928 | 0.002008 | 0.191401 | 0.024279 |
| Su (fu) | 0.005098 | 0.027700 | 0.002811 | 0.008397 | 0.014429 | 0.091446 | 0.000553 | 0.021556 |
| TAZ | 0.012952 | 0.000036 | 0.000905 | 0.000461 | 0.000799 | 0.003389 | 0.002583 | 0.078880 |
| TCFs | 0.025046 | 0.000231 | 0.000717 | 0.003711 | 0.004044 | 0.009630 | 0.002868 | 0.019232 |
| TEAD | 0.000597 | 0.000008 | 0.000029 | 0.000018 | 0.000104 | 0.001521 | 0.001316 | 0.061301 |
| p53 | 0.000122 | 0.000322 | 0.000187 | 0.000330 | 0.004066 | 0.002630 | 0.002827 | 0.018745 |
| TRADD | 0.000723 | 0.175453 | 0.438778 | 0.075879 | 0.038676 | 0.001426 | 0.006743 | 0.017041 |
| TRAF2 | 0.001592 | 0.242685 | 1.687458 | 0.000726 | 0.001859 | 0.001982 | 0.035266 | 0.011089 |
| TSC1 | 0.000102 | 0.004819 | 0.000013 | 0.001628 | 0.001002 | 0.000996 | 0.005967 | 0.003573 |
| TSC2 | 0.000004 | 0.000111 | 0.000002 | 0.000030 | 0.000094 | 0.000013 | 0.000295 | 0.000208 |
| Xsox17 | 0.000794 | 0.000031 | 0.000573 | 0.000034 | 0.000012 | 0.000347 | 0.000028 | 0.002279 |
| YAP | 0.001940 | 0.000094 | 0.001026 | 0.001178 | 0.001215 | 0.039319 | 0.005065 | 0.348074 |
| c-Myc | 0.000197 | 0.001986 | 0.000743 | 0.000045 | 0.000081 | 0.000941 | 0.003537 | 0.016925 |
| caspase 10 | 0.005123 | 0.086170 | 0.017511 | 0.008153 | 0.010807 | 0.000618 | 0.004262 | 0.064508 |
| caspase 3 | 0.000043 | 0.002817 | 0.002910 | 0.015468 | 0.005141 | 0.003649 | 0.025215 | 0.093111 |
| caspase 6 | 0.000003 | 0.000050 | 0.000302 | 0.000273 | 0.002355 | 0.000016 | 0.012673 | 0.001790 |
| caspase 7 | 0.000566 | 0.000548 | 0.021149 | 0.021815 | 0.093249 | 0.000864 | 0.000647 | 0.047280 |
| caspase 8 | 0.000115 | 0.010619 | 0.040327 | 0.009879 | 0.045855 | 0.002168 | 0.002558 | 0.015448 |
| caspase 9 | 0.000243 | 0.003168 | 0.004260 | 0.010740 | 0.067187 | 0.003288 | 0.008846 | 0.011363 |
| p15INK4b | 0.000045 | 0.000037 | 0.000000 | 0.000075 | 0.000162 | 0.001063 | 0.000006 | 0.000358 |
| p21cip1 | 0.000414 | 0.000188 | 0.000099 | 0.000024 | 0.060806 | 0.000060 | 0.001179 | 0.000085 |
| p27Kip1 | 0.000002 | 0.000100 | 0.000002 | 0.000234 | 0.000003 | 0.000000 | 0.008206 | 0.000033 |
| p38 | 0.002167 | 0.010366 | 0.002250 | 0.000966 | 0.005906 | 0.000539 | 0.028404 | 0.039367 |
