# Supplementary material for: Changes of signal transductivity and robustness of gene regulatory network in the carcinogenesis of leukemic subtypes via microarray sample data
Source: Oncotarget. 2018 May 4;9(34):23636–60. doi: 10.18632/oncotarget.25318 (PMC5955113; doi:10.18632/oncotarget.25318)
Supplement: Supplementary file 3 [file oncotarget-09-23636-s003.docx]

**Supplementary Table 2: The fold changes and variances of 159 proteins in the coupling STPs (Supplementary Figure 1)**

| Groups of proteins | Transductivity at MDS and AML  subtypes | | Fold change  calculated as the ratio of the mean of MDS or AML to normal subtype | | Variance for patients | | |
| --- | --- | --- | --- | --- | --- | --- | --- |
|  | MDS | AML | MDS | AML | Normal | MDS | AML |
| BCL9-2 | 0.000020 | 0.013390 | **1.0005** | **1.1564** | 38507601.5905 | 45789153.8561 | 101814673.7269 |
| PTEN | 0.006898 | 0.000051 | 0.9486 | **1.0688** | 55287724.2398 | 36261517.7741 | 40664869.5258 |
| ICAT | 0.005230 | 0.000344 | 0.9705 | **1.3125** | 7192713.3355 | 13387462.3428 | 36580789.7932 |
| ASPP2 | 0.000009 | 0.069634 | **1.0999** | 0.9819 | 9719002.0251 | 9492988.2473 | 19665393.0342 |
| p21cip1 | 0.001179 | 0.000060 | **1.0049** | **1.2519** | 2146358.3678 | 3177537.9050 | 6278091.8046 |
| C/EBP-α | 0.036069 | 0.001410 | 0.7726 | **1.1776** | 19217040.9522 | 848251.6070 | 5103862.4215 |
| JAK | 0.050690 | 0.314527 | **1.0141** | 0.7109 | 2360573.3461 | 3121966.5584 | 4898376.0598 |
| CSLs | 0.439170 | 0.226695 | 0.9552 | **1.3388** | 2557089.8625 | 1613980.7309 | 4393259.9013 |
| Smad4 | 0.060794 | 0.109012 | **1.0126** | **1.1638** | 1511200.1715 | 1819198.3072 | 4241388.3176 |
| p53 | 0.002827 | 0.002630 | 0.9656 | 0.7407 | 3223599.4665 | 3394733.6004 | 4040684.1349 |
| Ci | 0.000104 | 0.013218 | 0.9065 | **1.4001** | 1283884.6758 | 1335715.2407 | 3838243.7380 |
| Smo | 0.039861 | 0.001591 | **1.1480** | **1.1616** | 1474916.4806 | 2251091.3044 | 3320217.8408 |
| CIR | 0.052269 | 0.042544 | 0.9774 | **1.1067** | 1149132.7061 | 1040727.8384 | 3272102.4224 |
| c-Jun | 0.001598 | 0.000098 | 0.8378 | **1.1652** | 478433.4129 | 241517.1562 | 1318983.6369 |
| HATs | 0.011125 | 0.041400 | **1.0533** | 0.9497 | 368974.9417 | 790829.8725 | 1209579.1872 |
| CREBs | 0.001514 | 0.003265 | **1.0229** | **1.0434** | 620857.4193 | 963964.2092 | 1149413.9149 |
| TSC2 | 0.000295 | 0.000013 | **1.0545** | **1.2768** | 360010.5959 | 390539.2008 | 1141243.4729 |
| PP5 | 0.000927 | 0.001438 | **1.0302** | **1.2759** | 185250.7584 | 306115.4041 | 974109.2741 |
| MKK3 | 0.000824 | 0.000017 | 0.9146 | 0.9229 | 793205.1465 | 919871.0418 | 920484.0555 |
| caspase 8 | 0.002558 | 0.002168 | **1.0385** | 0.8533 | 730904.4010 | 677403.0215 | 854864.6320 |
| CRK | 0.256752 | 0.505419 | **1.0362** | 0.4875 | 789670.6585 | 698886.4613 | 811992.8753 |
| Receptor  -Frizzled | 1.624265 | 0.036230 | 0.9980 | **1.1031** | 257475.5144 | 379828.9347 | 800007.9430 |
| Bcl-2 | 0.000351 | 0.000018 | **1.0143** | **1.1189** | 406786.5069 | 322532.9061 | 750456.6220 |
| MTOR | 0.001231 | 0.000000 | 0.9476 | 0.7730 | 866352.7469 | 867038.1531 | 713250.2001 |
| TRAF2 | 0.035266 | 0.001982 | **1.0790** | **1.2894** | 111782.0780 | 208864.6434 | 619492.2211 |
| APC | 0.005881 | 0.073042 | 0.8642 | 0.9541 | 663779.0704 | 258703.1444 | 546163.8309 |
| CyclinD1 | 0.000028 | 0.000072 | 0.9763 | **1.0660** | 610721.5534 | 562089.5318 | 536531.4570 |
| PSE2 | 0.001603 | 0.001110 | 0.7813 | 0.7533 | 463276.5997 | 476386.8947 | 526652.8978 |
| FADD | 0.003916 | 0.005379 | **1.0654** | 0.9895 | 238328.9328 | 368709.1828 | 509083.2870 |
| PTP | 0.006962 | 0.000032 | 0.9335 | 0.9624 | 387531.2887 | 371562.0682 | 438966.2406 |
| p27Kip1 | 0.008206 | 0.000000 | 0.9696 | **1.2475** | 157396.6832 | 131935.9232 | 422894.1345 |
| Smad1 | 0.002243 | 0.007926 | **1.0345** | 0.9090 | 228235.8254 | 350680.0822 | 419768.6844 |
| SRF | 0.004672 | 0.009646 | **1.0853** | **1.1453** | 87607.2160 | 116156.2531 | 418918.3448 |
| PSEN | 0.300842 | 0.015173 | **1.0179** | **1.2985** | 167573.0327 | 248411.9352 | 411683.1995 |
| Duplin | 0.000417 | 0.000717 | **1.0061** | **1.3842** | 137054.9718 | 94531.6439 | 361139.8844 |
| IAPs | 0.001386 | 0.001101 | 0.9974 | **1.4904** | 62765.5653 | 68146.5817 | 342293.4065 |
| caspase 3 | 0.025215 | 0.003649 | 0.9125 | 0.8369 | 312405.1611 | 240386.9246 | 324960.9322 |
| PI3Kr | 0.202732 | 0.416272 | **1.0492** | 0.8091 | 242656.2821 | 255214.7783 | 320304.5239 |
| TRADD | 0.006743 | 0.001426 | **1.0093** | 0.9400 | 86438.4236 | 134580.2428 | 279418.6575 |
| c-Myc | 0.003537 | 0.000941 | **1.1123** | **1.9084** | 32126.9404 | 67252.5153 | 268162.0674 |
| Shc | 0.326294 | 0.418797 | **1.4077** | **1.9138** | 32417.2241 | 55332.7457 | 264974.3309 |
| CDK6 | 0.000064 | 0.000678 | 0.8799 | 0.6812 | 119824.3314 | 122413.2490 | 255554.2523 |
| AMPK | 0.002582 | 0.001080 | 0.9608 | **1.0158** | 152665.7464 | 131625.2203 | 249834.7196 |
| p38 | 0.028404 | 0.000539 | **1.0008** | 0.9885 | 227582.0072 | 215526.5314 | 246799.2386 |
| Smad3 | 0.069573 | 0.108902 | **1.1142** | **1.6567** | 25947.2297 | 72012.8525 | 241952.6546 |
| MKK4 | 0.007770 | 0.001279 | 0.8324 | 0.7306 | 264813.9132 | 188814.3807 | 214626.8603 |
| ASK1 | 0.138593 | 0.002681 | 0.9323 | **1.1848** | 99982.6133 | 65970.0174 | 208939.1426 |
| MAML | 0.036724 | 0.040191 | **1.0072** | 0.8299 | 215193.2757 | 199846.6428 | 194785.4421 |
| CDK4 | 0.000062 | 0.003470 | **1.0147** | 0.7588 | 82731.7912 | 129173.4129 | 193758.2780 |
| APH-1 | 0.020087 | 0.003274 | 0.9885 | 0.9572 | 79265.2894 | 105594.2834 | 191738.8928 |
| Receptor  -EGFR | 0.419742 | 0.353564 | **1.1736** | 0.9581 | 71538.3098 | 133640.0392 | 184891.8338 |
| EIF4E | 0.000086 | 0.000000 | **1.0671** | **1.3061** | 85122.2239 | 62402.8870 | 175429.8424 |
| CDK2 | 0.003965 | 0.000040 | 0.9437 | **1.1753** | 35043.3155 | 63063.0124 | 169296.0086 |
| ERK | 0.017666 | 0.005943 | 0.9618 | 0.9425 | 226486.4659 | 111054.1219 | 152683.9600 |
| Receptor  -PDGFR | 0.272482 | 0.082129 | **1.0672** | **2.0617** | 18784.7199 | 27977.9168 | 145484.5081 |
| TAZ | 0.002583 | 0.003389 | 0.8531 | 0.8760 | 135902.0724 | 69042.3369 | 134912.1628 |
| MNK | 0.006685 | 0.001209 | 0.7541 | 0.6746 | 114513.6530 | 83794.1356 | 133293.5703 |
| DAXX | 0.542258 | 0.004153 | **1.0050** | 0.6764 | 217648.9796 | 282772.2124 | 132876.4377 |
| Bim | 0.024815 | 0.001662 | **1.0732** | 0.9278 | 65208.3481 | 68068.3408 | 102909.7820 |
| MKK6 | 0.022358 | 0.000809 | **1.0363** | 0.8970 | 115089.7400 | 113879.6963 | 97546.1517 |
| PLZF | 0.000862 | 0.001171 | **1.0270** | **1.1213** | 41037.3138 | 37200.8083 | 95071.9718 |
| NCSTN | 0.152973 | 0.001168 | **1.2328** | **1.6708** | 18137.1123 | 24603.4926 | 82963.0450 |
| TEAD | 0.001316 | 0.001521 | **1.0846** | **1.0860** | 17357.1066 | 39462.8400 | 82306.4555 |
| Axin | 0.003679 | 0.174275 | 0.9253 | **1.0266** | 44939.7180 | 8468.6651 | 81366.5447 |
| PKA | 0.000002 | 0.000530 | **1.0017** | **1.0737** | 22456.8698 | 29913.5383 | 78334.7227 |
| p15INK4b | 0.000006 | 0.001063 | **1.0370** | 0.6335 | 90668.2723 | 64364.9375 | 77151.5734 |
| EVI1 | 0.005869 | 0.018376 | 0.9463 | 0.7505 | 47233.5447 | 85930.4461 | 73175.4982 |
| Slmb | 0.000005 | 0.000323 | **1.0289** | 0.8752 | 139059.3542 | 244645.5629 | 70122.9216 |
| EIF4B | 0.000000 | 0.000000 | **1.1863** | **1.5114** | 16031.4644 | 38613.1432 | 69512.1858 |
| Rab23 | 0.038848 | 0.000020 | **1.1634** | **1.2501** | 1680.5110 | 10330.3765 | 65713.7468 |
| LEF-1 | 0.003653 | 0.053655 | 0.9842 | **1.4734** | 7927.9473 | 7669.2214 | 65480.8753 |
| SKIP | 0.003701 | 0.003920 | **1.0337** | **1.3392** | 5316.3364 | 7644.2596 | 64906.7322 |
| YAP | 0.005065 | 0.039319 | **1.0733** | 0.9856 | 28466.2046 | 40825.4721 | 64184.5544 |
| BAD | 0.005371 | 0.000838 | 0.7264 | 0.8072 | 133157.0140 | 24034.8332 | 63456.0367 |
| Rb | 0.001235 | 0.000406 | 0.9879 | **1.4153** | 26651.6819 | 18487.2242 | 62600.5822 |
| CK1 | 0.008319 | 0.035109 | **1.0724** | **1.2629** | 8404.9762 | 14582.6995 | 59237.1209 |
| Receptor  -NOTCH | 0.309160 | 0.049787 | **1.0034** | **1.1636** | 23437.2918 | 28059.1038 | 57122.5904 |
| Smad2 | 0.042849 | 0.029741 | **1.0055** | **1.1573** | 29316.0220 | 25094.6520 | 54235.5565 |
| Max | 0.037128 | 0.000193 | 0.9919 | **1.2055** | 25534.1561 | 25162.9765 | 53823.0647 |
| NF-κBs | 0.035957 | 0.000046 | 0.8328 | **1.3924** | 13192.3375 | 8212.7899 | 53745.2424 |
| PHLPP | 0.004975 | 0.000074 | **1.0571** | **1.4902** | 10328.7489 | 13100.6307 | 52308.1681 |
| ETO | 0.023505 | 0.005942 | 0.9672 | **1.0012** | 32988.2655 | 16022.2023 | 51901.8151 |
| RAS | 0.111474 | 0.006297 | **1.0715** | **1.0955** | 21564.2263 | 35102.3158 | 49017.5769 |
| c-Fos | 0.000512 | 0.000004 | 0.9267 | 0.8325 | 45910.6915 | 32103.8481 | 38757.1743 |
| BCR | 1.569014 | 0.758114 | 0.9732 | **1.1734** | 23048.5420 | 16485.0574 | 37964.4074 |
| 4EBPs | 0.000575 | 0.000001 | 0.8980 | **1.0963** | 23464.5245 | 14868.8060 | 36363.0066 |
| HDAC | 0.038134 | 0.131872 | 0.7948 | 0.7039 | 123280.4349 | 43090.0228 | 35462.3967 |
| Receptor  -IGFR | 0.343310 | 0.342586 | **1.0169** | **1.2198** | 42056.1392 | 24831.3785 | 33850.7903 |
| MEK | 0.003906 | 0.000676 | **1.0484** | 0.9477 | 17410.0705 | 29329.9256 | 33037.8037 |
| MKP | 0.001719 | 0.000349 | **1.0426** | **1.3552** | 7121.6522 | 7109.5160 | 32460.7392 |
| AP-2α | 0.014974 | 0.001619 | **1.1677** | **1.5046** | 8213.1475 | 17765.2957 | 31527.2045 |
| MDM2 | 0.002987 | 0.002669 | 0.9018 | **1.0634** | 19812.6032 | 21383.3038 | 29024.5554 |
| ABL | 0.502623 | 0.167588 | **1.0780** | 0.9873 | 18697.9025 | 22348.8428 | 28123.5614 |
| PIP3 | 0.001054 | 0.014669 | 0.9921 | **1.0403** | 15014.6783 | 14630.4942 | 25968.1855 |
| caspase 7 | 0.000647 | 0.000864 | 0.9472 | **1.6280** | 9133.4133 | 5874.9921 | 25537.8795 |
| p300 | 0.017297 | 0.006481 | **1.1050** | 0.9802 | 24984.2643 | 31541.7849 | 25322.8278 |
| P70s6k | 0.000017 | 0.000000 | **1.0255** | 0.9696 | 11974.2163 | 14935.6572 | 24410.2187 |
| S6 | 0.000002 | 0.000000 | **1.0203** | **1.2237** | 8593.1059 | 7930.3853 | 23778.8759 |
| FLIP | 0.000013 | 0.000103 | 0.7934 | 0.8884 | 17592.7306 | 15197.4995 | 23053.3603 |
| Src family kinase | 0.191401 | 0.002008 | **1.0769** | **1.1558** | 15322.0058 | 23408.1219 | 22915.9372 |
| E2Fs | 0.000048 | 0.000016 | 0.9243 | 0.9730 | 13174.9520 | 10468.1474 | 22841.8180 |
| AKT | 0.042160 | 0.000789 | **1.2075** | 0.9043 | 15601.7769 | 34490.1039 | 22688.4443 |
| DFF45 | 0.000212 | 0.001357 | **1.0731** | **1.1328** | 7919.4621 | 10560.0627 | 21148.7395 |
| Receptor  -IL3 | 0.062797 | 0.047667 | **1.0018** | **1.3896** | 5407.9467 | 7432.6860 | 19356.8772 |
| Rheb | 0.001776 | 0.000022 | **1.0190** | **1.2942** | 4582.3106 | 5114.7748 | 19259.1362 |
| Receptor  -Fas | 0.139805 | 0.136634 | 0.9692 | 0.8379 | 15985.6642 | 21947.7104 | 18766.5798 |
| 14_3_3 | 0.001669 | 0.000042 | **1.0482** | 0.8298 | 31089.5956 | 26797.4385 | 18622.1799 |
| β-catenin | 0.019664 | 0.065852 | 0.9784 | **1.1905** | 3524.4405 | 7215.5579 | 18456.9922 |
| Ptc | 0.041651 | 0.049062 | **1.0248** | **1.0252** | 8463.7615 | 16941.0088 | 17040.9575 |
| Numb | 0.048760 | 0.003938 | **1.0639** | **1.2268** | 6602.0361 | 7641.9603 | 16924.1209 |
| SOS | 0.049613 | 0.092787 | 0.9927 | **1.0780** | 5658.8766 | 6597.1381 | 13206.8600 |
| CtBP | 0.018404 | 0.123317 | **1.1070** | **1.0713** | 3477.5690 | 5757.5064 | 12951.8785 |
| Miz1 | 0.001420 | 0.000025 | 0.9257 | **1.1463** | 3956.4619 | 5524.6472 | 12481.1914 |
| RIP1 | 0.002000 | 0.000021 | **1.2109** | 0.7380 | 8088.6012 | 21670.2798 | 12320.8685 |
| Receptor  -TNF | 0.027904 | 0.024267 | **1.0942** | **1.2079** | 4961.6129 | 5312.1151 | 12092.6249 |
| RSK2 | 0.008444 | 0.003518 | 0.9774 | **1.2786** | 1175.8102 | 2573.7216 | 11547.4493 |
| SMRT | 0.006901 | 0.001258 | **1.0837** | **1.1693** | 5876.5915 | 9131.0039 | 10391.0313 |
| GSK3β | 0.059375 | 0.294827 | **1.0311** | 0.9572 | 9139.5729 | 13001.6223 | 10144.8461 |
| AML1 | 0.006752 | 0.013406 | **1.1525** | 0.9662 | 4985.4890 | 8544.0875 | 10077.4155 |
| TCFs | 0.002868 | 0.009630 | **1.0686** | **1.0280** | 4558.3393 | 5863.8250 | 9307.2338 |
| STATs | 1.507073 | 0.837029 | 0.9715 | **1.0766** | 5256.4119 | 3805.5403 | 8796.1435 |
| caspase 10 | 0.004262 | 0.000618 | **1.0587** | **1.6329** | 1838.7874 | 1912.5619 | 8752.0146 |
| Receptor  -TGF-β | 0.331433 | 0.050038 | 0.8097 | 0.6632 | 27157.8938 | 16646.1030 | 8484.0304 |
| IKKs | 0.053726 | 0.000228 | **1.1028** | **1.0094** | 7749.3348 | 12665.0200 | 8356.0760 |
| Receptor  -FLT3 | 0.272218 | 0.063110 | **1.0933** | **1.1933** | 8914.9124 | 5238.3683 | 7967.5321 |
| JNK | 0.000674 | 0.001437 | 0.9182 | **1.2485** | 3816.6458 | 2903.0713 | 7745.3321 |
| Raptor | 0.000123 | 0.000003 | **1.0248** | **1.0700** | 3717.3392 | 3733.8323 | 7585.3273 |
| caspase 9 | 0.008846 | 0.003288 | **1.0902** | **1.4865** | 1751.3658 | 2970.6617 | 7525.2071 |
| Sapla | 0.011553 | 0.000321 | 0.9313 | **1.2342** | 1580.6375 | 1621.6543 | 5611.1513 |
| TSC1 | 0.005967 | 0.000996 | **1.1560** | 0.7626 | 4234.1615 | 6331.0950 | 5546.9962 |
| Mcl-1 | 0.019012 | 0.004894 | **1.0594** | 0.9754 | 3490.7345 | 4572.3880 | 5519.5739 |
| NIK | 0.009811 | 0.000387 | 0.7600 | 0.9366 | 5890.1450 | 3923.6437 | 5278.1377 |
| CyclinE | 0.002379 | 0.000003 | **1.0042** | **1.1161** | 1310.3772 | 1398.0982 | 5229.5463 |
| Bid | 0.000034 | 0.000030 | **1.0726** | **1.1564** | 1555.9619 | 3450.6413 | 5087.6166 |
| CKS1 | 0.000063 | 0.000013 | 0.9888 | **1.3398** | 1437.3713 | 1789.8595 | 4355.5215 |
| RAF | 0.054104 | 0.008852 | 0.9934 | 0.9196 | 2322.0881 | 3295.1184 | 3928.6765 |
| Receptor  -KIT | 0.346425 | 0.083514 | 0.8266 | **1.3256** | 1263.9942 | 823.9604 | 3906.4601 |
| LKB1 | 0.000084 | 0.000017 | **1.2547** | **1.3222** | 1753.6020 | 2780.3405 | 3621.7998 |
| Grb2 | 0.181267 | 0.540819 | 0.8729 | 0.9963 | 2152.1825 | 1702.7385 | 3478.1294 |
| PI3Kc | 0.055889 | 0.017392 | 0.9437 | **1.0724** | 1999.5602 | 2005.2815 | 3263.9980 |
| PP1 | 0.000174 | 0.000112 | 0.8852 | 0.9971 | 4058.8040 | 1494.5044 | 3135.2365 |
| Fu | 0.000263 | 0.005309 | 0.9833 | **1.3779** | 824.5036 | 1028.9173 | 2866.9300 |
| MEKK1 | 0.043057 | 0.005119 | **1.0220** | 0.8075 | 2000.2841 | 3070.6988 | 2526.0279 |
| P14arf | 0.001062 | 0.002465 | **1.0591** | 0.9157 | 1643.2204 | 2317.6440 | 2506.0674 |
| Xsox17 | 0.000028 | 0.000347 | 0.8660 | **1.2112** | 1768.7352 | 1073.2002 | 2481.5819 |
| PDK1 | 0.000777 | 0.000203 | **1.0043** | **1.1695** | 819.3342 | 788.3088 | 2444.5206 |
| Su (fu) | 0.000553 | 0.091446 | 0.8375 | **1.0104** | 1289.1508 | 960.6732 | 2319.9111 |
| PU.1 | 0.000250 | 0.004500 | 0.9240 | **1.0331** | 2641.2642 | 1758.9692 | 2265.7866 |
| DVL | 0.073907 | 0.367331 | 0.9605 | **1.4567** | 1195.2891 | 901.2907 | 2252.6093 |
| DFF40 | 0.000007 | 0.000011 | **1.0784** | 0.9694 | 1138.8485 | 3375.2224 | 1518.7221 |
| Deltex | 0.050137 | 0.010305 | **1.0748** | **1.0647** | 1112.6798 | 1564.7967 | 1335.6729 |
| Elk-1 | 0.012427 | 0.003398 | **1.0735** | **1.0908** | 826.9629 | 1061.6221 | 1299.8058 |
| caspase 6 | 0.012673 | 0.000016 | 0.9892 | **1.1184** | 1142.1256 | 903.4917 | 1142.1393 |
| Skp2 | 0.000084 | 0.000013 | 0.9228 | **1.1267** | 495.7405 | 424.7393 | 1012.5800 |
| GBL | 0.000548 | 0.000001 | 0.9943 | **1.2303** | 882.0242 | 769.4498 | 1004.7784 |
| HIF-1α | 0.000003 | 0.000000 | 0.9254 | 0.9064 | 631.3330 | 917.4969 | 694.3550 |
| FOXO3a | 0.025403 | 0.000018 | 0.9921 | **1.1175** | 560.4269 | 449.3475 | 676.4644 |
| IκB | 0.055336 | 0.000080 | 0.9503 | 0.9379 | 862.0694 | 292.9012 | 662.7837 |
| Bcl-XL | 0.000021 | 0.000121 | 0.9337 | **1.0402** | 543.9618 | 363.0673 | 375.6692 |
