# Supplementary material for: Changes of signal transductivity and robustness of gene regulatory network in the carcinogenesis of leukemic subtypes via microarray sample data
Source: Oncotarget. 2018 May 4;9(34):23636–60. doi: 10.18632/oncotarget.25318 (PMC5955113; doi:10.18632/oncotarget.25318)
Supplement: Supplementary file 4 [file oncotarget-09-23636-s004.docx]

**Supplementary Table 3: The proteins belong to the 159 groups of proteins in the model of coupling STPs**

| Groups  of proteins | Proteins |
| --- | --- |
| 14-3-3 | YWHAE/YWHAQ |
| 4EBPs | EIF4EBP1 |
| ABL | ABL1 |
| AKT | AKT1/AKT2/AKT3 |
| AML1 | RUNX1 |
| AMPK | PRKAA1/PRKAA2 |
| AP-2α | TFAP2A |
| APC | APC |
| APH-1 | APH1B/APH1A |
| ASK1 | MAP3K5 |
| ASPP2 | TP53BP2 |
| Axin | AXIN1/AXIN2 |
| BAD | BAD |
| β-catenin | CTNNB1 |
| Bcl-2 | BCL2 |
| BCL9-2 | BCL9 |
| Bcl-XL | BCL2L1 |
| BCR | BCR |
| Bid | BID |
| Bim | BCL2L11 |
| C/EBP-α | CEBPA |
| caspase 10 | CASP10 |
| caspase 3 | CASP3 |
| caspase 6 | CASP6 |
| caspase 7 | CASP7 |
| caspase 8 | CASP8 |
| caspase 9 | CASP9 |
| p300 | CREBBP/EP300 |
| CDK2 | CDK2 |
| CDK4 | CDK4 |
| CDK6 | CDK6 |
| Ci | GLI1/GLI2/GLI3 |
| CIR | CIR1 |
| CK1 | CSNK1A1/CSNK1E/CSNK1D/CSNK1G1/CSNK1G2/CSNK1G3 |
| CKS1 | CKS1B/CKS2 |
| c-Myc | MYC |
| CREBs | ATF4/CREB1/CREB3/CREB3L1/CREB3L2/CREB3L3/CREB3L4/CREB5 |
| CRK | CBL/CBLB/CBLC/CRK/CRKL |
| CSLs | RBPJ/RBPJL |
| CtBP | CTBP1/CTBP2 |
| CyclinD1 | CCND1 |
| CyclinE | CCNE1/CCNE2 |
| DAXX | DAXX |
| Deltex | DTX1/DTX2/DTX3/DTX3L/DTX4 |
| DFF40 | DFFB |
| DFF45 | DFFA |
| Duplin | CHD8 |
| DVL | DVL1/DVL2/DVL3 |
| E2Fs | E2F1/E2F2/E2F3 |
| EIF4B | EIF4B |
| EIF4E | EIF4E/EIF4E2 |
| Elk-1 | ELK1 |
| ERK | MAPK1/MAPK3 |
| ETO | RUNX1T1 |
| EVI1 | MECOM |
| FADD | FADD |
| FLIP | CFLAR |
| c-Fos | FOS |
| FOXO3a | FOXO3 |
| Fu | STK36 |
| GBL | MLST8 |
| Grb2 | GRB2 |
| GSK3β | GSK3B |
| HATs | KAT2A/KAT2B |
| HDAC | HDAC1/HDAC2 |
| HIF-1α | HIF1A |
| IAPs | BIRC2/BIRC3/BIRC7/XIAP |
| ICAT | CTNNBIP1 |
| IκB | NFKBIA |
| IKKs | CHUK/IKBKB/IKBKG |
| JAK | JAK1/JAK2/JAK3 |
| c-Jun | JUN |
| JNK | MAPK8/MAPK9/MAPK10 |
| LEF-1 | LEF1 |
| LKB1 | STK11 |
| MAML | MAML1/MAML2/MAML3 |
| Max | MAX |
| Mcl-1 | MCL1 |
| MDM2 | MDM2 |
| MEK | MAP2K1/MAP2K2 |
| MEKK1 | MAP3K1 |
| Miz1 | PIAS2 |
| MKK3 | MAP2K3 |
| MKK4 | MAP2K4 |
| MKK6 | MAP2K6 |
| MKP | DUSP1/DUSP2/DUSP4/DUSP5/DUSP6/DUSP7/DUSP8/DUSP9/  DUSP10/DUSP16 |
| MNK | MKNK1/MKNK2 |
| MTOR | MTOR |
| NCSTN | NCSTN |
| NF-κBs | NFKB1/NFKB2/REL/RELA/RELB |
| NIK | MAP3K14 |
| Numb | NUMB/NUMBL |
| P14arf | CDKN2A |
| p15INK4b | CDKN2B |
| p21cip1 | CDKN1A |
| p27Kip1 | CDKN1B |
| p38 | MAPK11/MAPK12/MAPK13/MAPK14 |
| P70s6k | RPS6KB1/RPS6KB2 |
| PDK1 | PDPK1 |
| PHLPP | PHLPP1/PHLPP2 |
| PI3Kc | PIK3CA/PIK3CB/PIK3CD/PIK3CG |
| PI3Kr | PIK3R1/PIK3R2/PIK3R3/PIK3R4/PIK3R5/PIK3R6 |
| PIP3 | PIP5K1A/PIP5K1B/PIP5K1C |
| PKA | PRKACA/PRKACB/PRKACG/PRKX |
| PLZF | ZBTB16 |
| PP1 | PPP1CA/PPP1CB/PPP1CC |
| PP5 | PPP5C |
| PSE2 | PSENEN |
| PSEN | PSEN1/PSEN2 |
| Ptc | PTCH1 |
| PTEN | PTEN |
| PTP | DUSP3/PTPN5/PTPN7/PTPRR |
| PU.1 | SPI1 |
| Rab23 | RAB23 |
| RAF | ARAF/BRAF/RAF1 |
| Raptor | RPTOR |
| RAS | HRAS/KRAS/NRAS |
| Rb | RB1 |
| Receptor-EGFR | EGFR |
| Receptor-Fas | FAS |
| Receptor-FLT3 | FLT3 |
| Receptor-Frizzled | FZD1/FZD2/FZD3/FZD4/FZD5/FZD6/FZD7/FZD8/FZD9/FZD10 |
| Receptor-IGFR | IGF1R |
| Receptor-IL3 | CSF2RB/IL3RA |
| Receptor-KIT | KIT |
| Receptor-NOTCH | NOTCH1/NOTCH2/NOTCH3/NOTCH4 |
| Receptor-PDGFR | PDGFRA/PDGFRB |
| Receptor-TGF-β | TGFBR1/TGFBR2 |
| Receptor-TNF | TNFRSF1A |
| Rheb | RHEB |
| RIP1 | RIPK1 |
| RSK2 | RPS6KA1/RPS6KA2/RPS6KA3/RPS6KA6 |
| S6 | RPS6 |
| Sapla | ELK4 |
| Shc | SHC1/SHC3/SHC4 |
| SKIP | SNW1 |
| Skp2 | SKP2 |
| Slmb | BTRC/FBXW11 |
| Smad1 | SMAD1 |
| Smad2 | SMAD2 |
| Smad3 | SMAD3 |
| Smad4 | SMAD4 |
| Smo | SMO |
| SMRT | NCOR2 |
| SOS | SOS1/SOS2 |
| Src  family kinase | BLK/FGR/FRK/FYN/HCK/LCK/SRC/YES1 |
| SRF | SRF |
| STATs | STAT5A/STAT5B/STAT1/STAT3 |
| Su(fu) | SUFU |
| TAZ | WWTR1 |
| TCFs | TCF7/TCF7L1/TCF7L2 |
| TEAD | TEAD1/TEAD2/TEAD3/TEAD4 |
| p53 | TP53 |
| TRADD | TRADD |
| TRAF2 | TRAF2 |
| TSC1 | TSC1 |
| TSC2 | TSC2 |
| Xsox17 | SOX17 |
| YAP | YAP1 |
