# Supplementary material for: Changes of signal transductivity and robustness of gene regulatory network in the carcinogenesis of leukemic subtypes via microarray sample data
Source: Oncotarget. 2018 May 4;9(34):23636–60. doi: 10.18632/oncotarget.25318 (PMC5955113; doi:10.18632/oncotarget.25318)
Supplement: Supplementary file 5 [file oncotarget-09-23636-s005.docx]

**Supplementary Table 4: The full table of effectiveness of drugs for treating patients with AML/MDS**

| Drugs | Effectiveness for treating AML | N | Drugs | Effectiveness for treating MDS | N |
| --- | --- | --- | --- | --- | --- |
| terbutaline | 6.469 | 4 | STOCK1N-35696 | 9.071 | 2 |
| arachidonic acid | 6.016 | 3 | nomegestrol | 8.488 | 3 |
| PHA-00846566E | 5.431 | 3 | proadifen | 8.473 | 4 |
| dimethyloxalylglycine | 5.326 | 1 | atracurium besilate | 8.421 | 3 |
| alprostadil | 5.214 | 7 | methocarbamol | 8.275 | 3 |
| Prestwick-1100 | 4.990 | 4 | dienestrol | 8.185 | 3 |
| clofibrate | 4.954 | 2 | CP-863187 | 8.107 | 4 |
| PNU-0230031 | 4.782 | 8 | azacitidine | 8.019 | 3 |
| PF-00539758-00 | 4.666 | 3 | arachidonyltrifluoromethane | 8.018 | 2 |
| ketoconazole | 4.554 | 4 | imidurea | 7.993 | 3 |
| prednicarbate | 4.482 | 3 | MS-275 | 7.931 | 2 |
| ketanserin | 4.323 | 4 | methylergometrine | 7.877 | 4 |
| ifosfamide | 4.318 | 3 | PF-00875133-00 | 7.765 | 3 |
| azacitidine | 4.287 | 3 | PHA-00816795 | 7.624 | 2 |
| CP-320650-01 | 4.124 | 8 | cefamandole | 7.583 | 4 |
| SC-560 | 3.886 | 3 | paroxetine | 7.474 | 4 |
| rosiglitazone | 3.876 | 14 | irinotecan | 7.406 | 3 |
| NS-398 | 3.869 | 3 | oxolamine | 7.390 | 4 |
| oligomycin | 3.803 | 1 | rifabutin | 7.380 | 3 |
| mometasone | 3.741 | 4 | tonzonium bromide | 7.268 | 4 |
| 6-bromoindirubin-3-oxime | 3.722 | 7 | vanoxerine | 7.149 | 4 |
| tyrphostin AG-825 | 3.646 | 1 | trapidil | 7.062 | 3 |
| 3-aminobenzamide | 3.633 | 1 | metaraminol | 7.005 | 4 |
| U0125 | 3.618 | 1 | labetalol | 6.923 | 4 |
| TTNPB | 3.611 | 2 | clofazimine | 6.872 | 5 |
| topiramate | 3.556 | 1 | amoxicillin | 6.839 | 4 |
| levomepromazine | 3.529 | 4 | lisuride | 6.822 | 5 |
| nomegestrol | 3.523 | 3 | 16,16-dimethylprostaglandin E2 | 6.783 | 3 |
| 1,5-isoquinolinediol | 3.510 | 1 | mepenzolate bromide | 6.758 | 5 |
| Y-27632 | 3.412 | 2 | 0297417-0002B | 6.711 | 3 |
| cefamandole | 3.412 | 4 | PF-01378883-00 | 6.685 | 4 |
| 12,13-EODE | 3.400 | 1 | thapsigargin | 6.684 | 3 |
| MS-275 | 3.390 | 2 | H-89 | 6.661 | 3 |
| CP-319743 | 3.344 | 4 | stachydrine | 6.652 | 4 |
| HNMPA-(AM)3 | 3.286 | 1 | etoposide | 6.648 | 4 |
| semustine | 3.283 | 4 | PHA-00767505E | 6.601 | 4 |
| atracurium besilate | 3.256 | 3 | aminohippuric acid | 6.568 | 4 |
| DL-PPMP | 3.246 | 1 | pyrimethamine | 6.528 | 5 |
| pimozide | 3.176 | 4 | AH-23848 | 6.528 | 3 |
| monorden | 3.132 | 22 | MG-262 | 6.510 | 3 |
| hydrocotarnine | 3.127 | 4 | raubasine | 6.494 | 4 |
| mitoxantrone | 3.113 | 3 | pioglitazone | 6.489 | 11 |
| nimesulide | 3.104 | 4 | nimesulide | 6.487 | 4 |
| vincamine | 3.088 | 6 | 15(S)-15-methylprostaglandin E2 | 6.482 | 4 |
| genistein | 3.085 | 17 | minaprine | 6.440 | 5 |
| aminohippuric acid | 3.062 | 4 | sanguinarine | 6.337 | 2 |
| vanoxerine | 2.995 | 4 | nifedipine | 6.336 | 7 |
| sulfinpyrazone | 2.984 | 4 | calycanthine | 6.245 | 4 |
| IC-86621 | 2.904 | 4 | butein | 6.244 | 2 |
| nialamide | 2.892 | 4 | clobetasol | 6.223 | 3 |
| acetylsalicylic acid | 2.860 | 13 | beclometasone | 6.193 | 3 |
| fasudil | 2.842 | 2 | nicergoline | 6.168 | 5 |
| methylergometrine | 2.798 | 4 | chlorambucil | 6.161 | 4 |
| scriptaid | 2.791 | 3 | cyanocobalamin | 6.129 | 4 |
| C-75 | 2.689 | 4 | flunixin | 6.119 | 5 |
| fluocinonide | 2.683 | 5 | clofibrate | 6.118 | 2 |
| butein | 2.672 | 2 | methoxsalen | 6.040 | 3 |
| STOCK1N-35696 | 2.663 | 2 | budesonide | 5.984 | 4 |
| ketorolac | 2.625 | 4 | hydrastinine | 5.964 | 5 |
| folic acid | 2.609 | 4 | depudecin | 5.912 | 2 |
| budesonide | 2.601 | 4 | propafenone | 5.882 | 4 |
| rotenone | 2.595 | 4 | alcuronium chloride | 5.881 | 2 |
| PHA-00745360 | 2.588 | 8 | corynanthine | 5.852 | 3 |
| suramin sodium | 2.567 | 4 | prednicarbate | 5.827 | 3 |
| cromoglicic acid | 2.498 | 2 | Prestwick-1103 | 5.824 | 4 |
| imidurea | 2.475 | 3 | Prestwick-981 | 5.807 | 3 |
| (-)-catechin | 2.468 | 1 | 8-azaguanine | 5.805 | 4 |
| tioguanine | 2.450 | 1 | digoxigenin | 5.800 | 5 |
| niclosamide | 2.448 | 5 | clofilium tosylate | 5.792 | 3 |
| clofilium tosylate | 2.424 | 3 | dihydroergotamine | 5.785 | 5 |
| homosalate | 2.397 | 4 | risperidone | 5.776 | 3 |
| benserazide | 2.378 | 5 | fluvoxamine | 5.772 | 4 |
| vorinostat | 2.353 | 12 | amantadine | 5.752 | 4 |
| spaglumic acid | 2.350 | 2 | 2,6-dimethylpiperidine | 5.741 | 5 |
| oxaprozin | 2.324 | 6 | Prestwick-1082 | 5.724 | 3 |
| geldanamycin | 2.320 | 15 | semustine | 5.699 | 4 |
| oxamic acid | 2.298 | 1 | azathioprine | 5.691 | 7 |
| sulindac sulfide | 2.298 | 1 | gentamicin | 5.667 | 4 |
| 2,6-dimethylpiperidine | 2.291 | 5 | rosiglitazone | 5.606 | 14 |
| nifedipine | 2.289 | 7 | exisulind | 5.596 | 2 |
| hydrocortisone | 2.278 | 3 | Prestwick-1080 | 5.562 | 4 |
| 0175029-0000 | 2.259 | 6 | oxetacaine | 5.533 | 5 |
| Prestwick-1082 | 2.254 | 3 | luteolin | 5.528 | 4 |
| Gly-His-Lys | 2.222 | 3 | STOCK1N-35874 | 5.486 | 2 |
| alfaxalone | 2.216 | 3 | NS-398 | 5.484 | 3 |
| 10-methoxyharmalan | 2.206 | 4 | lidoflazine | 5.478 | 3 |
| Prestwick-675 | 2.198 | 4 | acetylsalicylsalicylic acid | 5.474 | 4 |
| pirinixic acid | 2.183 | 5 | lidocaine | 5.473 | 5 |
| gentamicin | 2.166 | 4 | resveratrol | 5.471 | 9 |
| 11-deoxy-16,16-dimethylprostaglandin E2 | 2.156 | 4 | levomepromazine | 5.470 | 4 |
| N-phenylanthranilic acid | 2.154 | 1 | benserazide | 5.444 | 5 |
| chlortalidone | 2.103 | 4 | molsidomine | 5.441 | 4 |
| MG-132 | 2.102 | 1 | lumicolchicine | 5.383 | 3 |
| lincomycin | 2.065 | 3 | ampicillin | 5.376 | 4 |
| decitabine | 2.058 | 1 | diclofenac | 5.361 | 5 |
| cicloheximide | 2.046 | 4 | 10-methoxyharmalan | 5.342 | 4 |
| fenbendazole | 2.022 | 4 | PNU-0230031 | 5.338 | 8 |
| PF-00539745-00 | 1.978 | 3 | PF-00539758-00 | 5.330 | 3 |
| tolazoline | 1.977 | 5 | homochlorcyclizine | 5.319 | 4 |
| trimethylcolchicinic acid | 1.971 | 4 | blebbistatin | 5.314 | 2 |
| azathioprine | 1.969 | 7 | wortmannin | 5.309 | 2 |
| pararosaniline | 1.956 | 1 | anabasine | 5.304 | 3 |
| troglitazone | 1.951 | 16 | Prestwick-984 | 5.293 | 4 |
| fulvestrant | 1.939 | 28 | metitepine | 5.286 | 4 |
| chlorambucil | 1.926 | 4 | picotamide | 5.283 | 5 |
| calycanthine | 1.904 | 4 | fulvestrant | 5.227 | 28 |
| PHA-00665752 | 1.903 | 1 | salsolidin | 5.225 | 4 |
| acetylsalicylsalicylic acid | 1.895 | 4 | hydroxyzine | 5.206 | 5 |
| demecolcine | 1.874 | 1 | MK-886 | 5.200 | 2 |
| splitomicin | 1.873 | 1 | PNU-0293363 | 5.186 | 3 |
| chlormezanone | 1.872 | 4 | loracarbef | 5.167 | 4 |
| paroxetine | 1.862 | 4 | racecadotril | 5.157 | 4 |
| levcycloserine | 1.844 | 4 | scoulerine | 5.146 | 4 |
| betazole | 1.799 | 5 | oxaprozin | 5.145 | 6 |
| methocarbamol | 1.789 | 3 | 4,5-dianilinophthalimide | 5.138 | 2 |
| thiocolchicoside | 1.784 | 4 | dizocilpine | 5.128 | 5 |
| ampyrone | 1.781 | 5 | lincomycin | 5.123 | 3 |
| rifabutin | 1.743 | 3 | aminocaproic acid | 5.119 | 3 |
| tyrphostin AG-1478 | 1.717 | 1 | bupropion | 5.101 | 4 |
| PHA-00851261E | 1.712 | 8 | ciprofloxacin | 5.098 | 5 |
| cytochalasin B | 1.706 | 1 | isradipine | 5.084 | 4 |
| triflusal | 1.676 | 3 | dextromethorphan | 5.073 | 4 |
| monastrol | 1.667 | 8 | tolfenamic acid | 5.068 | 4 |
| celastrol | 1.664 | 1 | thalidomide | 5.057 | 7 |
| metampicillin | 1.620 | 5 | talampicillin | 5.053 | 4 |
| BW-B70C | 1.616 | 1 | captopril | 5.050 | 5 |
| camptothecin | 1.605 | 3 | dihydroergocristine | 5.038 | 4 |
| arachidonyltrifluoromethane | 1.603 | 2 | calmidazolium | 5.036 | 2 |
| 0173570-0000 | 1.594 | 6 | nimodipine | 5.036 | 4 |
| daunorubicin | 1.591 | 4 | metampicillin | 5.032 | 5 |
| fusaric acid | 1.590 | 4 | lysergol | 5.031 | 4 |
| cephaeline | 1.586 | 5 | homosalate | 5.027 | 4 |
| netilmicin | 1.584 | 4 | ascorbic acid | 5.024 | 4 |
| CP-863187 | 1.581 | 4 | cytochalasin B | 5.016 | 1 |
| cimetidine | 1.552 | 5 | esculetin | 5.004 | 3 |
| mefloquine | 1.535 | 5 | mercaptopurine | 5.000 | 2 |
| ciprofloxacin | 1.522 | 5 | AH-6809 | 4.999 | 2 |
| isocorydine | 1.502 | 4 | daunorubicin | 4.995 | 4 |
| corynanthine | 1.492 | 3 | corbadrine | 4.979 | 4 |
| 4,5-dianilinophthalimide | 1.485 | 2 | AG-012559 | 4.960 | 3 |
| erastin | 1.480 | 4 | corticosterone | 4.959 | 4 |
| 2-deoxy-D-glucose | 1.479 | 1 | levcycloserine | 4.956 | 4 |
| tolfenamic acid | 1.477 | 4 | sulfachlorpyridazine | 4.954 | 5 |
| vinblastine | 1.463 | 3 | PHA-00846566E | 4.937 | 3 |
| methacholine chloride | 1.458 | 3 | butoconazole | 4.935 | 4 |
| butirosin | 1.451 | 4 | clonidine | 4.933 | 4 |
| ipratropium bromide | 1.432 | 3 | demeclocycline | 4.930 | 6 |
| meteneprost | 1.403 | 4 | docosahexaenoic acid ethyl ester | 4.926 | 2 |
| phenanthridinone | 1.382 | 1 | PF-00539745-00 | 4.925 | 3 |
| noretynodrel | 1.372 | 4 | progesterone | 4.925 | 4 |
| dirithromycin | 1.353 | 3 | chlorphenamine | 4.918 | 4 |
| tubocurarine chloride | 1.348 | 4 | butirosin | 4.916 | 4 |
| resveratrol | 1.315 | 9 | fasudil | 4.893 | 2 |
| fisetin | 1.311 | 1 | 3-nitropropionic acid | 4.886 | 4 |
| cinnarizine | 1.278 | 4 | canrenoic acid | 4.885 | 4 |
| mycophenolic acid | 1.267 | 3 | proscillaridin | 4.876 | 3 |
| nicergoline | 1.253 | 5 | 0225151-0000 | 4.874 | 3 |
| santonin | 1.240 | 4 | camptothecin | 4.873 | 3 |
| androsterone | 1.240 | 4 | etomidate | 4.860 | 3 |
| aceclofenac | 1.232 | 4 | levopropoxyphene | 4.853 | 4 |
| flufenamic acid | 1.222 | 6 | apigenin | 4.844 | 4 |
| lidocaine | 1.216 | 5 | tamoxifen | 4.840 | 7 |
| melatonin | 1.212 | 4 | ketoconazole | 4.828 | 4 |
| tonzonium bromide | 1.184 | 4 | verapamil | 4.824 | 6 |
| phenyl biguanide | 1.172 | 1 | bergenin | 4.787 | 4 |
| lycorine | 1.170 | 5 | tetraethylenepentamine | 4.776 | 6 |
| pioglitazone | 1.169 | 11 | SB-203580 | 4.758 | 5 |
| piperidolate | 1.169 | 3 | trimetazidine | 4.756 | 4 |
| LM-1685 | 1.169 | 3 | AG-013608 | 4.747 | 8 |
| verteporfin | 1.142 | 3 | cytisine | 4.743 | 4 |
| oxolinic acid | 1.136 | 5 | ketanserin | 4.736 | 4 |
| exisulind | 1.119 | 2 | methylbenzethonium chloride | 4.727 | 6 |
| spiperone | 1.102 | 2 | HNMPA-(AM)3 | 4.718 | 1 |
| tomelukast | 1.090 | 1 | cyclizine | 4.711 | 4 |
| chlorhexidine | 1.088 | 5 | CP-944629 | 4.701 | 4 |
| lansoprazole | 1.084 | 4 | mitoxantrone | 4.700 | 3 |
| deferoxamine | 1.083 | 8 | aceclofenac | 4.696 | 4 |
| cefoxitin | 1.081 | 4 | (+)-chelidonine | 4.695 | 4 |
| methoxsalen | 1.062 | 3 | folic acid | 4.692 | 4 |
| ikarugamycin | 1.060 | 3 | triflusal | 4.689 | 3 |
| alvespimycin | 1.054 | 12 | mecamylamine | 4.683 | 3 |
| pyrimethamine | 1.051 | 5 | spiradoline | 4.676 | 4 |
| piperacillin | 1.040 | 5 | CP-319743 | 4.670 | 4 |
| metaraminol | 1.019 | 4 | lobelanidine | 4.667 | 4 |
| clomipramine | 1.017 | 4 | lobeline | 4.662 | 4 |
| PHA-00816795 | 1.013 | 2 | chloroquine | 4.661 | 4 |
| ganciclovir | 1.001 | 4 | rottlerin | 4.660 | 3 |
| beclometasone | 0.987 | 3 | hexamethonium bromide | 4.641 | 5 |
| 15-delta prostaglandin J2 | 0.985 | 15 | zalcitabine | 4.633 | 4 |
| metitepine | 0.961 | 4 | lansoprazole | 4.622 | 4 |
| cycloserine | 0.950 | 4 | Prestwick-1083 | 4.621 | 3 |
| copper sulfate | 0.948 | 4 | dexamethasone | 4.617 | 8 |
| carmustine | 0.920 | 3 | isoconazole | 4.604 | 5 |
| valdecoxib | 0.902 | 3 | hesperetin | 4.596 | 5 |
| flunixin | 0.894 | 5 | alpha-yohimbine | 4.590 | 3 |
| stachydrine | 0.887 | 4 | ouabain | 4.581 | 4 |
| yohimbine | 0.875 | 5 | scriptaid | 4.552 | 3 |
| flutamide | 0.869 | 5 | methylprednisolone | 4.548 | 4 |
| clozapine | 0.866 | 17 | quipazine | 4.544 | 4 |
| 16,16-dimethylprostaglandin E2 | 0.862 | 3 | phenazone | 4.529 | 3 |
| tracazolate | 0.860 | 4 | ciclosporin | 4.500 | 6 |
| quinpirole | 0.857 | 4 | lanatoside C | 4.499 | 6 |
| labetalol | 0.850 | 4 | dantrolene | 4.494 | 6 |
| corbadrine | 0.839 | 4 | pentetrazol | 4.490 | 4 |
| citalopram | 0.837 | 4 | sodium phenylbutyrate | 4.474 | 7 |
| gefitinib | 0.832 | 1 | lomustine | 4.470 | 4 |
| naftifine | 0.821 | 4 | chlortalidone | 4.468 | 4 |
| clobetasol | 0.811 | 3 | guanabenz | 4.466 | 5 |
| HC toxin | 0.796 | 1 | niclosamide | 4.458 | 5 |
| indometacin | 0.764 | 8 | DL-thiorphan | 4.458 | 2 |
| halofantrine | 0.737 | 3 | valdecoxib | 4.454 | 3 |
| estradiol | 0.733 | 15 | amylocaine | 4.444 | 5 |
| chrysin | 0.733 | 3 | gliclazide | 4.443 | 4 |
| CAY-10397 | 0.711 | 3 | ranitidine | 4.428 | 5 |
| clorgiline | 0.696 | 4 | mimosine | 4.422 | 3 |
| gossypol | 0.683 | 6 | alfaxalone | 4.422 | 3 |
| dipyridamole | 0.676 | 6 | naringenin | 4.406 | 4 |
| aciclovir | 0.671 | 6 | vinblastine | 4.404 | 3 |
| mecamylamine | 0.666 | 3 | NU-1025 | 4.402 | 2 |
| SR-95639A | 0.664 | 4 | tetrahydroalstonine | 4.399 | 4 |
| diclofenac | 0.646 | 5 | ketotifen | 4.396 | 4 |
| phenformin | 0.628 | 7 | tremorine | 4.396 | 4 |
| sulfafurazole | 0.628 | 5 | timolol | 4.389 | 4 |
| dexverapamil | 0.621 | 1 | troleandomycin | 4.387 | 4 |
| lomustine | 0.610 | 4 | Prestwick-1100 | 4.381 | 4 |
| PHA-00767505E | 0.598 | 4 | betamethasone | 4.378 | 3 |
| hycanthone | 0.596 | 4 | colchicine | 4.371 | 6 |
| H-89 | 0.589 | 3 | flucloxacillin | 4.369 | 4 |
| danazol | 0.578 | 4 | PHA-00745360 | 4.362 | 8 |
| lobelanidine | 0.568 | 4 | harmalol | 4.359 | 3 |
| vinburnine | 0.555 | 4 | meprylcaine | 4.358 | 4 |
| spiradoline | 0.550 | 4 | alfadolone | 4.347 | 3 |
| josamycin | 0.549 | 5 | tracazolate | 4.344 | 4 |
| dioxybenzone | 0.548 | 4 | BW-B70C | 4.340 | 1 |
| seneciphylline | 0.547 | 4 | melatonin | 4.336 | 4 |
| naphazoline | 0.527 | 5 | sulfaquinoxaline | 4.335 | 3 |
| propidium iodide | 0.511 | 4 | bacampicillin | 4.330 | 4 |
| mianserin | 0.507 | 5 | acacetin | 4.314 | 6 |
| parbendazole | 0.504 | 4 | pirenzepine | 4.309 | 5 |
| desipramine | 0.487 | 4 | diflunisal | 4.304 | 5 |
| cefoperazone | 0.479 | 3 | MG-132 | 4.295 | 1 |
| blebbistatin | 0.474 | 2 | colforsin | 4.289 | 5 |
| ampicillin | 0.473 | 4 | chlorpropamide | 4.287 | 6 |
| PF-00875133-00 | 0.472 | 3 | Prestwick-691 | 4.287 | 3 |
| cytisine | 0.471 | 4 | carisoprodol | 4.285 | 4 |
| pentetrazol | 0.453 | 4 | pivampicillin | 4.281 | 4 |
| orlistat | 0.452 | 5 | terbutaline | 4.278 | 4 |
| rottlerin | 0.450 | 3 | vidarabine | 4.270 | 4 |
| khellin | 0.419 | 5 | protriptyline | 4.263 | 4 |
| calmidazolium | 0.413 | 2 | dirithromycin | 4.248 | 3 |
| cyclopenthiazide | 0.412 | 4 | betaxolol | 4.242 | 4 |
| clemastine | 0.411 | 3 | phenanthridinone | 4.235 | 1 |
| triflupromazine | 0.410 | 4 | Prestwick-559 | 4.231 | 3 |
| clofazimine | 0.408 | 5 | econazole | 4.220 | 4 |
| tropicamide | 0.399 | 6 | diprophylline | 4.220 | 5 |
| BCB000039 | 0.396 | 3 | bisoprolol | 4.217 | 4 |
| AR-A014418 | 0.386 | 3 | tolazamide | 4.197 | 3 |
| harmine | 0.385 | 4 | oxolinic acid | 4.193 | 5 |
| MG-262 | 0.378 | 3 | W-13 | 4.192 | 2 |
| atropine methonitrate | 0.335 | 3 | piperacillin | 4.191 | 5 |
| pirlindole | 0.328 | 3 | ethosuximide | 4.191 | 4 |
| oxamniquine | 0.323 | 4 | parthenolide | 4.190 | 4 |
| oxytetracycline | 0.319 | 3 | DL-PPMP | 4.183 | 1 |
| scoulerine | 0.314 | 4 | simvastatin | 4.179 | 4 |
| adenosine phosphate | 0.310 | 4 | etofylline | 4.179 | 5 |
| 0317956-0000 | 0.308 | 8 | pimozide | 4.160 | 4 |
| haloperidol | 0.305 | 32 | penbutolol | 4.156 | 3 |
| maprotiline | 0.291 | 4 | rotenone | 4.148 | 4 |
| salsolidin | 0.275 | 4 | lasalocid | 4.147 | 4 |
| ciprofibrate | 0.245 | 4 | tropicamide | 4.145 | 6 |
| colforsin | 0.241 | 5 | suloctidil | 4.145 | 4 |
| harmalol | 0.238 | 3 | oxybenzone | 4.128 | 4 |
| proscillaridin | 0.238 | 3 | fludroxycortide | 4.113 | 5 |
| karakoline | 0.235 | 6 | piperidolate | 4.093 | 3 |
| edrophonium chloride | 0.228 | 5 | physostigmine | 4.093 | 4 |
| piromidic acid | 0.224 | 4 | (-)-MK-801 | 4.082 | 4 |
| AG-028671 | 0.218 | 3 | noretynodrel | 4.066 | 4 |
| (+)-chelidonine | 0.203 | 4 | phenformin | 4.050 | 7 |
| lanatoside C | 0.171 | 6 | citiolone | 4.046 | 6 |
| bromocriptine | 0.166 | 5 | pirinixic acid | 4.040 | 5 |
| AH-23848 | 0.164 | 3 | CP-320650-01 | 4.035 | 8 |
| alcuronium chloride | 0.158 | 2 | spiperone | 4.032 | 2 |
| cyproheptadine | 0.148 | 5 | furaltadone | 4.019 | 6 |
| gliclazide | 0.131 | 4 | carbimazole | 4.009 | 3 |
| isradipine | 0.123 | 4 | brinzolamide | 4.009 | 4 |
| hydroxyzine | 0.109 | 5 | sulfametoxydiazine | 3.994 | 4 |
| alpha-yohimbine | 0.098 | 3 | amphotericin B | 3.984 | 4 |
| kanamycin | 0.096 | 4 | ionomycin | 3.975 | 3 |
| rimexolone | 0.093 | 4 | ticlopidine | 3.974 | 5 |
| dienestrol | 0.089 | 3 | TTNPB | 3.971 | 2 |
| methylprednisolone | 0.075 | 4 | bacitracin | 3.969 | 3 |
| 3-nitropropionic acid | 0.073 | 4 | clemastine | 3.962 | 3 |
| latamoxef | 0.068 | 3 | ticarcillin | 3.951 | 3 |
| lysergol | 0.055 | 4 | cinnarizine | 3.947 | 4 |
| 0198306-0000 | 0.053 | 4 | PHA-00851261E | 3.928 | 8 |
| STOCK1N-35215 | 0.048 | 3 | AG-028671 | 3.920 | 3 |
| 4-hydroxyphenazone | 0.040 | 5 | antimycin A | 3.919 | 5 |
| domperidone | 0.030 | 4 | thiocolchicoside | 3.917 | 4 |
| PNU-0293363 | 0.018 | 3 | alprostadil | 3.911 | 7 |
| atropine oxide | 0.001 | 5 | cefazolin | 3.906 | 5 |
| pseudopelletierine | -0.002 | 4 | C-75 | 3.903 | 4 |
| hemicholinium | -0.009 | 4 | ornidazole | 3.895 | 5 |
| oxybutynin | -0.010 | 4 | streptomycin | 3.882 | 4 |
| Prestwick-689 | -0.021 | 4 | lynestrenol | 3.880 | 5 |
| esculetin | -0.023 | 3 | verteporfin | 3.870 | 3 |
| depudecin | -0.026 | 2 | haloperidol | 3.867 | 32 |
| trazodone | -0.030 | 3 | vinburnine | 3.858 | 4 |
| sulfasalazine | -0.038 | 5 | IC-86621 | 3.855 | 4 |
| lorglumide | -0.042 | 5 | 16-phenyltetranorprostaglandin E2 | 3.850 | 4 |
| metronidazole | -0.051 | 5 | decitabine | 3.846 | 1 |
| cloxacillin | -0.085 | 4 | iopromide | 3.836 | 4 |
| CP-944629 | -0.093 | 4 | nitrendipine | 3.826 | 5 |
| cyclizine | -0.115 | 4 | tyrphostin AG-1478 | 3.815 | 1 |
| ethaverine | -0.126 | 4 | mafenide | 3.809 | 5 |
| ranitidine | -0.131 | 5 | chlorphenesin | 3.797 | 4 |
| trapidil | -0.142 | 3 | sulindac | 3.796 | 7 |
| glipizide | -0.154 | 5 | chlorhexidine | 3.792 | 5 |
| Prestwick-559 | -0.158 | 3 | Prestwick-920 | 3.773 | 4 |
| 0297417-0002B | -0.175 | 3 | denatonium benzoate | 3.770 | 4 |
| phenazone | -0.177 | 3 | lycorine | 3.768 | 5 |
| sulfachlorpyridazine | -0.186 | 5 | trimipramine | 3.743 | 4 |
| 0316684-0000 | -0.188 | 4 | isoflupredone | 3.740 | 3 |
| Trolox C | -0.189 | 4 | hecogenin | 3.737 | 4 |
| carbamazepine | -0.189 | 8 | quinostatin | 3.731 | 2 |
| minoxidil | -0.194 | 5 | metronidazole | 3.725 | 5 |
| lynestrenol | -0.203 | 5 | spaglumic acid | 3.719 | 2 |
| econazole | -0.206 | 4 | acepromazine | 3.711 | 4 |
| moroxydine | -0.222 | 5 | bepridil | 3.695 | 4 |
| sulfapyridine | -0.233 | 4 | pridinol | 3.692 | 4 |
| pyrvinium | -0.236 | 6 | fosfosal | 3.691 | 4 |
| etoposide | -0.237 | 4 | cyclopenthiazide | 3.688 | 4 |
| pentolonium | -0.237 | 5 | demecolcine | 3.686 | 1 |
| kaempferol | -0.257 | 4 | estrone | 3.682 | 4 |
| risperidone | -0.258 | 3 | adenosine phosphate | 3.678 | 4 |
| alexidine | -0.281 | 4 | SR-95531 | 3.677 | 4 |
| amodiaquine | -0.293 | 4 | albendazole | 3.676 | 3 |
| astemizole | -0.295 | 5 | ivermectin | 3.670 | 5 |
| cyanocobalamin | -0.296 | 4 | thiamine | 3.659 | 3 |
| proglumide | -0.296 | 5 | equilin | 3.656 | 5 |
| digoxin | -0.312 | 4 | clomipramine | 3.652 | 4 |
| phenoxybenzamine | -0.327 | 4 | ursolic acid | 3.646 | 4 |
| bisoprolol | -0.330 | 4 | sulfasalazine | 3.643 | 5 |
| harmol | -0.330 | 4 | mometasone | 3.639 | 4 |
| cefadroxil | -0.332 | 4 | oxymetazoline | 3.632 | 4 |
| methotrexate | -0.338 | 8 | propylthiouracil | 3.623 | 4 |
| nimodipine | -0.353 | 4 | phenoxybenzamine | 3.617 | 4 |
| brinzolamide | -0.354 | 4 | dimethyloxalylglycine | 3.613 | 1 |
| sanguinarine | -0.355 | 2 | SB-202190 | 3.601 | 5 |
| bacitracin | -0.361 | 3 | laudanosine | 3.600 | 4 |
| benzydamine | -0.376 | 4 | deferoxamine | 3.594 | 8 |
| amikacin | -0.378 | 4 | dioxybenzone | 3.577 | 4 |
| zalcitabine | -0.396 | 4 | berberine | 3.573 | 4 |
| bergenin | -0.400 | 4 | phentolamine | 3.570 | 7 |
| CP-690334-01 | -0.403 | 8 | loperamide | 3.567 | 6 |
| betonicine | -0.405 | 6 | glipizide | 3.566 | 5 |
| imipenem | -0.409 | 4 | monocrotaline | 3.563 | 4 |
| tiratricol | -0.412 | 4 | thiamphenicol | 3.557 | 5 |
| BCB000040 | -0.415 | 4 | tocainide | 3.551 | 4 |
| nystatin | -0.420 | 3 | fenspiride | 3.550 | 5 |
| nisoxetine | -0.430 | 4 | iobenguane | 3.538 | 4 |
| amantadine | -0.438 | 4 | Gly-His-Lys | 3.518 | 3 |
| PNU-0251126 | -0.441 | 6 | (-)-catechin | 3.513 | 1 |
| midecamycin | -0.443 | 5 | cefoperazone | 3.512 | 3 |
| zoxazolamine | -0.444 | 4 | quinidine | 3.505 | 3 |
| bucladesine | -0.444 | 6 | staurosporine | 3.504 | 2 |
| dihydroergocristine | -0.447 | 4 | U0125 | 3.489 | 1 |
| piroxicam | -0.451 | 4 | fludrocortisone | 3.481 | 8 |
| sulfadoxine | -0.454 | 3 | cimetidine | 3.466 | 5 |
| menadione | -0.454 | 2 | atropine | 3.457 | 4 |
| naringenin | -0.457 | 4 | sertaconazole | 3.454 | 4 |
| STOCK1N-35874 | -0.461 | 2 | harpagoside | 3.452 | 4 |
| CP-645525-01 | -0.462 | 3 | quinethazone | 3.450 | 4 |
| diprophylline | -0.467 | 5 | kanamycin | 3.440 | 4 |
| sulfamethoxazole | -0.476 | 5 | podophyllotoxin | 3.425 | 4 |
| talampicillin | -0.481 | 4 | benzbromarone | 3.424 | 3 |
| corticosterone | -0.485 | 4 | cinchocaine | 3.415 | 5 |
| dexamethasone | -0.486 | 8 | pyrantel | 3.408 | 5 |
| dopamine | -0.497 | 1 | dexpropranolol | 3.406 | 3 |
| fluvoxamine | -0.497 | 4 | dacarbazine | 3.402 | 4 |
| flucloxacillin | -0.506 | 4 | fendiline | 3.400 | 3 |
| ornidazole | -0.508 | 5 | geldanamycin | 3.392 | 15 |
| hexestrol | -0.513 | 4 | methyldopa | 3.374 | 5 |
| diphenylpyraline | -0.518 | 6 | harmol | 3.368 | 4 |
| alprenolol | -0.531 | 4 | N6-methyladenosine | 3.365 | 4 |
| hesperetin | -0.546 | 5 | perphenazine | 3.353 | 5 |
| mafenide | -0.546 | 5 | imatinib | 3.353 | 2 |
| 0225151-0000 | -0.553 | 3 | ioxaglic acid | 3.345 | 3 |
| diltiazem | -0.555 | 5 | iodixanol | 3.329 | 3 |
| ketoprofen | -0.573 | 6 | pimethixene | 3.318 | 3 |
| tamoxifen | -0.574 | 7 | furazolidone | 3.315 | 4 |
| oxolamine | -0.582 | 4 | oxytetracycline | 3.311 | 3 |
| triamcinolone | -0.589 | 5 | citalopram | 3.301 | 4 |
| PF-00562151-00 | -0.591 | 8 | fenoterol | 3.300 | 3 |
| streptomycin | -0.592 | 4 | scopoletin | 3.298 | 2 |
| tranexamic acid | -0.598 | 5 | 0175029-0000 | 3.293 | 6 |
| heptaminol | -0.604 | 5 | ciprofibrate | 3.291 | 4 |
| pinacidil | -0.604 | 4 | pararosaniline | 3.277 | 1 |
| AG-012559 | -0.648 | 3 | santonin | 3.275 | 4 |
| imipramine | -0.650 | 4 | milrinone | 3.267 | 3 |
| minaprine | -0.657 | 5 | cephaeline | 3.264 | 5 |
| berberine | -0.662 | 4 | sulconazole | 3.264 | 4 |
| mebeverine | -0.665 | 4 | capsaicin | 3.259 | 4 |
| mepacrine | -0.671 | 2 | etamsylate | 3.245 | 4 |
| Prestwick-1103 | -0.671 | 4 | edrophonium chloride | 3.243 | 5 |
| withaferin A | -0.671 | 4 | PF-00562151-00 | 3.238 | 8 |
| acetazolamide | -0.672 | 4 | ritodrine | 3.237 | 4 |
| chlorphenamine | -0.673 | 4 | tolazoline | 3.224 | 5 |
| sulfamerazine | -0.674 | 5 | pheneticillin | 3.219 | 4 |
| paclitaxel | -0.678 | 6 | PHA-00665752 | 3.210 | 1 |
| flecainide | -0.681 | 6 | trimethylcolchicinic acid | 3.195 | 4 |
| chloroquine | -0.696 | 4 | aminophenazone | 3.176 | 5 |
| pyrantel | -0.697 | 5 | alvespimycin | 3.174 | 12 |
| oxetacaine | -0.700 | 5 | piperine | 3.163 | 4 |
| amoxapine | -0.711 | 5 | tyrphostin AG-825 | 3.162 | 1 |
| novobiocin | -0.729 | 9 | tubocurarine chloride | 3.126 | 4 |
| Prestwick-1084 | -0.737 | 4 | quinpirole | 3.123 | 4 |
| carcinine | -0.743 | 4 | hydrastine hydrochloride | 3.121 | 4 |
| bupropion | -0.747 | 4 | baclofen | 3.120 | 5 |
| glycocholic acid | -0.751 | 4 | hyoscyamine | 3.118 | 5 |
| proadifen | -0.751 | 4 | acetylsalicylic acid | 3.116 | 13 |
| hydroquinine | -0.754 | 4 | tetracaine | 3.098 | 3 |
| fipexide | -0.764 | 3 | ampyrone | 3.097 | 5 |
| H-7 | -0.770 | 4 | midodrine | 3.091 | 5 |
| ioversol | -0.775 | 4 | cefalexin | 3.084 | 5 |
| 15(S)-15-methylprostaglandin E2 | -0.775 | 4 | amoxapine | 3.081 | 5 |
| cefotiam | -0.776 | 4 | orlistat | 3.081 | 5 |
| docosahexaenoic acid ethyl ester | -0.778 | 2 | pyrvinium | 3.081 | 6 |
| amoxicillin | -0.786 | 4 | fipexide | 3.072 | 3 |
| estrone | -0.794 | 4 | cefepime | 3.066 | 4 |
| NU-1025 | -0.803 | 2 | ifosfamide | 3.060 | 3 |
| Prestwick-665 | -0.807 | 5 | 12,13-EODE | 3.058 | 1 |
| methyldopa | -0.818 | 5 | splitomicin | 3.056 | 1 |
| irinotecan | -0.827 | 3 | torasemide | 3.052 | 4 |
| zaprinast | -0.829 | 4 | cinchonine | 3.052 | 4 |
| oxymetazoline | -0.841 | 4 | apomorphine | 3.051 | 5 |
| dehydrocholic acid | -0.844 | 5 | amprolium | 3.050 | 5 |
| ethosuximide | -0.846 | 4 | famotidine | 3.044 | 5 |
| cyclopentolate | -0.850 | 4 | astemizole | 3.023 | 5 |
| pyrithyldione | -0.855 | 4 | Prestwick-983 | 3.012 | 3 |
| 0179445-0000 | -0.857 | 8 | flutamide | 3.010 | 5 |
| acepromazine | -0.857 | 4 | meclofenamic acid | 3.008 | 5 |
| physostigmine | -0.859 | 4 | diphenhydramine | 2.998 | 5 |
| sulfamonomethoxine | -0.859 | 4 | SC-560 | 2.982 | 3 |
| isoxsuprine | -0.861 | 5 | withaferin A | 2.973 | 4 |
| mesoridazine | -0.872 | 4 | genistein | 2.972 | 17 |
| wortmannin | -0.876 | 2 | aciclovir | 2.970 | 6 |
| etacrynic acid | -0.881 | 3 | Trolox C | 2.966 | 4 |
| ambroxol | -0.884 | 4 | celastrol | 2.965 | 1 |
| etomidate | -0.886 | 3 | fisetin | 2.953 | 1 |
| fluoxetine | -0.891 | 4 | cyproheptadine | 2.947 | 5 |
| tetrahydroalstonine | -0.894 | 4 | chenodeoxycholic acid | 2.940 | 4 |
| oxedrine | -0.894 | 4 | naloxone | 2.940 | 6 |
| oleandomycin | -0.902 | 5 | boldine | 2.937 | 4 |
| medrysone | -0.905 | 6 | tranexamic acid | 2.924 | 5 |
| hydrastine hydrochloride | -0.906 | 4 | lymecycline | 2.915 | 4 |
| primaquine | -0.906 | 4 | chlorzoxazone | 2.913 | 4 |
| lumicolchicine | -0.907 | 3 | BAS-012416453 | 2.912 | 3 |
| nalidixic acid | -0.912 | 5 | triamcinolone | 2.875 | 5 |
| lisuride | -0.924 | 5 | norfloxacin | 2.860 | 5 |
| fosfosal | -0.932 | 4 | alimemazine | 2.859 | 4 |
| metoclopramide | -0.945 | 6 | parbendazole | 2.858 | 4 |
| puromycin | -0.947 | 4 | tolnaftate | 2.843 | 5 |
| phenylpropanolamine | -0.948 | 4 | artemisinin | 2.843 | 3 |
| chlorpropamide | -0.949 | 6 | puromycin | 2.829 | 4 |
| mefexamide | -0.963 | 4 | PNU-0251126 | 2.826 | 6 |
| pergolide | -0.976 | 4 | ebselen | 2.812 | 3 |
| quinethazone | -0.978 | 4 | dapsone | 2.807 | 5 |
| cefsulodin | -0.980 | 4 | paracetamol | 2.798 | 4 |
| SR-95531 | -0.982 | 4 | 0317956-0000 | 2.797 | 8 |
| dihydroergotamine | -0.992 | 5 | roxarsone | 2.789 | 4 |
| perphenazine | -0.992 | 5 | atropine methonitrate | 2.776 | 3 |
| dizocilpine | -1.009 | 5 | dobutamine | 2.775 | 4 |
| hyoscyamine | -1.013 | 5 | fluocinonide | 2.773 | 5 |
| imatinib | -1.015 | 2 | nomifensine | 2.768 | 5 |
| Prestwick-981 | -1.031 | 3 | dequalinium chloride | 2.767 | 4 |
| cefapirin | -1.033 | 4 | procyclidine | 2.760 | 4 |
| fusidic acid | -1.034 | 4 | adiphenine | 2.739 | 5 |
| disulfiram | -1.040 | 5 | fusaric acid | 2.723 | 4 |
| ellipticine | -1.054 | 4 | monastrol | 2.723 | 8 |
| glimepiride | -1.059 | 4 | heptaminol | 2.717 | 5 |
| sotalol | -1.079 | 4 | morantel | 2.717 | 5 |
| iodixanol | -1.084 | 3 | indometacin | 2.696 | 8 |
| methapyrilene | -1.087 | 4 | clorsulon | 2.678 | 4 |
| anabasine | -1.087 | 3 | convolamine | 2.671 | 4 |
| Prestwick-920 | -1.091 | 4 | colecalciferol | 2.670 | 4 |
| metformin | -1.093 | 10 | zoxazolamine | 2.657 | 4 |
| pirenzepine | -1.095 | 5 | cyproterone | 2.635 | 4 |
| pheneticillin | -1.113 | 4 | viomycin | 2.634 | 4 |
| ketotifen | -1.117 | 4 | sulfapyridine | 2.629 | 4 |
| colchicine | -1.122 | 6 | etacrynic acid | 2.628 | 3 |
| pivampicillin | -1.123 | 4 | prilocaine | 2.626 | 6 |
| loperamide | -1.125 | 6 | etilefrine | 2.618 | 4 |
| procyclidine | -1.127 | 4 | clioquinol | 2.612 | 5 |
| gallamine triethiodide | -1.127 | 5 | fenofibrate | 2.582 | 3 |
| racecadotril | -1.128 | 4 | phenindione | 2.566 | 4 |
| prochlorperazine | -1.128 | 16 | myosmine | 2.564 | 6 |
| ticarcillin | -1.146 | 3 | gabexate | 2.554 | 4 |
| raubasine | -1.149 | 4 | pentoxifylline | 2.540 | 5 |
| spironolactone | -1.153 | 5 | medrysone | 2.537 | 6 |
| halcinonide | -1.160 | 5 | nisoxetine | 2.528 | 4 |
| pentoxyverine | -1.170 | 4 | halofantrine | 2.526 | 3 |
| bacampicillin | -1.178 | 4 | prenylamine | 2.515 | 4 |
| celecoxib | -1.179 | 5 | tyloxapol | 2.511 | 4 |
| aminoglutethimide | -1.183 | 3 | metergoline | 2.508 | 4 |
| etodolac | -1.183 | 5 | acetazolamide | 2.492 | 4 |
| scopolamine | -1.185 | 4 | benzamil | 2.485 | 6 |
| famprofazone | -1.187 | 6 | zardaverine | 2.485 | 4 |
| tanespimycin | -1.201 | 62 | estropipate | 2.484 | 4 |
| eticlopride | -1.207 | 4 | betahistine | 2.484 | 4 |
| azacyclonol | -1.207 | 5 | trimethobenzamide | 2.480 | 5 |
| norfloxacin | -1.208 | 5 | velnacrine | 2.466 | 4 |
| clopamide | -1.208 | 4 | cloperastine | 2.465 | 6 |
| ursolic acid | -1.209 | 4 | solasodine | 2.458 | 6 |
| kawain | -1.210 | 5 | cinchonidine | 2.455 | 4 |
| arcaine | -1.216 | 4 | Prestwick-675 | 2.438 | 4 |
| acacetin | -1.218 | 6 | androsterone | 2.435 | 4 |
| glycopyrronium bromide | -1.220 | 5 | levamisole | 2.432 | 4 |
| naltrexone | -1.221 | 5 | fusidic acid | 2.426 | 4 |
| naloxone | -1.222 | 6 | tinidazole | 2.425 | 6 |
| suloctidil | -1.230 | 4 | meclofenoxate | 2.423 | 6 |
| streptozocin | -1.231 | 4 | dihydrostreptomycin | 2.422 | 5 |
| gibberellic acid | -1.232 | 4 | clopamide | 2.422 | 4 |
| trimethadione | -1.234 | 4 | ikarugamycin | 2.407 | 3 |
| GW-8510 | -1.238 | 4 | nystatin | 2.407 | 3 |
| PF-01378883-00 | -1.242 | 4 | spironolactone | 2.405 | 5 |
| furosemide | -1.242 | 4 | riboflavin | 2.405 | 4 |
| protriptyline | -1.248 | 4 | cromoglicic acid | 2.405 | 2 |
| zidovudine | -1.249 | 4 | alexidine | 2.401 | 4 |
| thiamine | -1.250 | 3 | rescinnamine | 2.399 | 3 |
| cantharidin | -1.252 | 1 | nizatidine | 2.394 | 4 |
| promethazine | -1.253 | 4 | tiaprofenic acid | 2.390 | 4 |
| SC-58125 | -1.255 | 4 | hydrocortisone | 2.389 | 3 |
| iobenguane | -1.257 | 4 | benfluorex | 2.372 | 4 |
| guanabenz | -1.264 | 5 | niridazole | 2.372 | 4 |
| thiamphenicol | -1.267 | 5 | seneciphylline | 2.368 | 4 |
| emetine | -1.272 | 4 | metamizole sodium | 2.364 | 6 |
| riluzole | -1.290 | 5 | tomelukast | 2.356 | 1 |
| fenbufen | -1.292 | 6 | pempidine | 2.347 | 5 |
| furaltadone | -1.296 | 6 | atractyloside | 2.341 | 5 |
| mepenzolate bromide | -1.300 | 5 | meticrane | 2.340 | 5 |
| mimosine | -1.302 | 3 | spiramycin | 2.333 | 6 |
| antimycin A | -1.310 | 5 | 1,4-chrysenequinone | 2.331 | 2 |
| etilefrine | -1.315 | 4 | netilmicin | 2.329 | 4 |
| adiphenine | -1.319 | 5 | bumetanide | 2.325 | 4 |
| benzbromarone | -1.323 | 3 | aconitine | 2.323 | 4 |
| apigenin | -1.328 | 4 | josamycin | 2.319 | 5 |
| syrosingopine | -1.330 | 4 | CP-645525-01 | 2.318 | 3 |
| sodium phenylbutyrate | -1.331 | 7 | ceftazidime | 2.316 | 3 |
| triamterene | -1.332 | 5 | strophanthidin | 2.280 | 4 |
| ajmaline | -1.346 | 3 | BCB000040 | 2.280 | 4 |
| quinostatin | -1.348 | 2 | digitoxigenin | 2.278 | 4 |
| nocodazole | -1.349 | 6 | carmustine | 2.272 | 3 |
| troleandomycin | -1.350 | 4 | methapyrilene | 2.268 | 4 |
| fludrocortisone | -1.352 | 8 | Prestwick-864 | 2.263 | 4 |
| valproic acid | -1.366 | 57 | nocodazole | 2.257 | 6 |
| clotrimazole | -1.368 | 5 | estriol | 2.246 | 4 |
| salbutamol | -1.370 | 5 | pentamidine | 2.232 | 5 |
| meclofenamic acid | -1.375 | 5 | chrysin | 2.227 | 3 |
| amylocaine | -1.382 | 5 | methotrexate | 2.222 | 8 |
| SB-202190 | -1.383 | 5 | colistin | 2.213 | 4 |
| tetraethylenepentamine | -1.388 | 6 | pentolonium | 2.212 | 5 |
| sulfametoxydiazine | -1.388 | 4 | pargyline | 2.211 | 4 |
| cefalexin | -1.391 | 5 | azapropazone | 2.200 | 3 |
| digoxigenin | -1.397 | 5 | esculin | 2.198 | 4 |
| acebutolol | -1.398 | 5 | sulfafurazole | 2.194 | 5 |
| azlocillin | -1.399 | 4 | STOCK1N-35215 | 2.191 | 3 |
| pheniramine | -1.401 | 5 | ganciclovir | 2.178 | 4 |
| dyclonine | -1.402 | 4 | benzonatate | 2.166 | 5 |
| ciclacillin | -1.404 | 4 | nitrofurantoin | 2.166 | 5 |
| proxymetacaine | -1.417 | 4 | harmine | 2.160 | 4 |
| chlortetracycline | -1.417 | 5 | 3-hydroxy-DL-kynurenine | 2.148 | 6 |
| sirolimus | -1.423 | 44 | SR-95639A | 2.146 | 4 |
| naftidrofuryl | -1.432 | 4 | doxazosin | 2.144 | 4 |
| fenspiride | -1.446 | 5 | lorglumide | 2.144 | 5 |
| pindolol | -1.447 | 5 | phenyl biguanide | 2.138 | 1 |
| BCB000038 | -1.449 | 4 | diperodon | 2.134 | 3 |
| isoconazole | -1.468 | 5 | hydroquinine | 2.131 | 4 |
| Prestwick-691 | -1.470 | 3 | testosterone | 2.128 | 5 |
| chlorzoxazone | -1.473 | 4 | dicycloverine | 2.122 | 5 |
| parthenolide | -1.475 | 4 | ergocalciferol | 2.104 | 4 |
| nitrofural | -1.475 | 4 | raloxifene | 2.103 | 7 |
| levobunolol | -1.478 | 4 | terazosin | 2.099 | 4 |
| bethanechol | -1.481 | 4 | beta-escin | 2.094 | 6 |
| diphenhydramine | -1.482 | 5 | sulfinpyrazone | 2.091 | 4 |
| 16-phenyltetranorprostaglandin E2 | -1.484 | 4 | paclitaxel | 2.089 | 6 |
| simvastatin | -1.485 | 4 | procaine | 2.084 | 5 |
| demecarium bromide | -1.487 | 4 | nabumetone | 2.080 | 4 |
| AG-013608 | -1.489 | 8 | dicloxacillin | 2.068 | 4 |
| enoxacin | -1.502 | 4 | thioridazine | 2.066 | 20 |
| AH-6809 | -1.506 | 2 | betazole | 2.061 | 5 |
| suxibuzone | -1.507 | 4 | acebutolol | 2.056 | 5 |
| atovaquone | -1.515 | 3 | megestrol | 2.044 | 4 |
| torasemide | -1.529 | 4 | carbenoxolone | 2.043 | 4 |
| isopropamide iodide | -1.534 | 4 | copper sulfate | 2.034 | 4 |
| esculin | -1.540 | 4 | roxithromycin | 2.031 | 4 |
| tolazamide | -1.559 | 3 | thiostrepton | 2.031 | 4 |
| nomifensine | -1.563 | 5 | sulfaguanidine | 2.028 | 5 |
| griseofulvin | -1.564 | 5 | indoprofen | 2.015 | 4 |
| thapsigargin | -1.585 | 3 | amodiaquine | 2.010 | 4 |
| trimetazidine | -1.588 | 4 | cisapride | 2.009 | 4 |
| quercetin | -1.591 | 6 | praziquantel | 2.000 | 4 |
| monocrotaline | -1.591 | 4 | betulinic acid | 1.985 | 4 |
| capsaicin | -1.603 | 4 | doxepin | 1.977 | 3 |
| lidoflazine | -1.605 | 3 | hesperidin | 1.968 | 4 |
| doxepin | -1.606 | 3 | yohimbine | 1.960 | 5 |
| metrizamide | -1.606 | 4 | suramin sodium | 1.960 | 4 |
| laudanosine | -1.606 | 4 | coralyne | 1.957 | 4 |
| ceftazidime | -1.608 | 3 | chlorcyclizine | 1.947 | 6 |
| cinoxacin | -1.612 | 4 | ambroxol | 1.940 | 4 |
| myosmine | -1.615 | 6 | diltiazem | 1.940 | 5 |
| heliotrine | -1.623 | 6 | naftifine | 1.931 | 4 |
| quipazine | -1.631 | 4 | debrisoquine | 1.926 | 4 |
| chlorphenesin | -1.632 | 4 | digoxin | 1.925 | 4 |
| amrinone | -1.633 | 4 | suprofen | 1.911 | 4 |
| carbimazole | -1.637 | 3 | cefotetan | 1.910 | 3 |
| dydrogesterone | -1.638 | 4 | Prestwick-674 | 1.910 | 6 |
| aminocaproic acid | -1.641 | 3 | levonorgestrel | 1.906 | 6 |
| zimeldine | -1.653 | 5 | suxibuzone | 1.902 | 4 |
| Prestwick-1083 | -1.661 | 3 | tranylcypromine | 1.902 | 5 |
| procarbazine | -1.669 | 3 | azlocillin | 1.893 | 4 |
| Chicago Sky Blue 6B | -1.676 | 4 | gallamine triethiodide | 1.891 | 5 |
| ticlopidine | -1.677 | 5 | butamben | 1.890 | 4 |
| betahistine | -1.683 | 4 | pregnenolone | 1.889 | 4 |
| F0447-0125 | -1.689 | 4 | guaifenesin | 1.886 | 6 |
| antazoline | -1.690 | 4 | pinacidil | 1.876 | 4 |
| crotamiton | -1.696 | 4 | practolol | 1.872 | 4 |
| cisapride | -1.704 | 4 | sulfathiazole | 1.868 | 5 |
| alpha-ergocryptine | -1.705 | 6 | oxyphenbutazone | 1.867 | 4 |
| carisoprodol | -1.706 | 4 | syrosingopine | 1.865 | 4 |
| procainamide | -1.708 | 4 | benzathine benzylpenicillin | 1.865 | 4 |
| tretinoin | -1.709 | 22 | fluphenazine | 1.863 | 18 |
| methanthelinium bromide | -1.710 | 4 | cicloheximide | 1.849 | 4 |
| fenoprofen | -1.711 | 6 | cloxacillin | 1.838 | 4 |
| papaverine | -1.714 | 4 | kaempferol | 1.830 | 4 |
| DL-thiorphan | -1.715 | 2 | Y-27632 | 1.827 | 2 |
| verapamil | -1.716 | 6 | flupentixol | 1.824 | 4 |
| fendiline | -1.727 | 3 | dimethadione | 1.823 | 4 |
| apomorphine | -1.729 | 5 | primaquine | 1.819 | 4 |
| sulfabenzamide | -1.729 | 4 | adipiodone | 1.818 | 4 |
| debrisoquine | -1.733 | 4 | sulfamethoxypyridazine | 1.807 | 5 |
| amphotericin B | -1.733 | 4 | xamoterol | 1.806 | 3 |
| prenylamine | -1.758 | 4 | cotinine | 1.801 | 6 |
| clorsulon | -1.761 | 4 | sulindac sulfide | 1.789 | 1 |
| methylbenzethonium chloride | -1.771 | 6 | ginkgolide A | 1.789 | 4 |
| prednisolone | -1.779 | 5 | 15-delta prostaglandin J2 | 1.782 | 15 |
| tetroquinone | -1.779 | 4 | piromidic acid | 1.770 | 4 |
| denatonium benzoate | -1.786 | 4 | betonicine | 1.768 | 6 |
| arecoline | -1.789 | 4 | chlorprothixene | 1.768 | 4 |
| captopril | -1.789 | 5 | gabapentin | 1.767 | 4 |
| butoconazole | -1.795 | 4 | imipenem | 1.766 | 4 |
| spectinomycin | -1.796 | 4 | meropenem | 1.764 | 4 |
| iloprost | -1.803 | 3 | ribostamycin | 1.757 | 4 |
| lomefloxacin | -1.806 | 6 | pralidoxime | 1.748 | 4 |
| alverine | -1.809 | 4 | 0173570-0000 | 1.744 | 6 |
| dl-alpha tocopherol | -1.812 | 4 | quercetin | 1.715 | 6 |
| bezafibrate | -1.817 | 4 | thiamazole | 1.704 | 6 |
| hesperidin | -1.818 | 4 | flurbiprofen | 1.702 | 5 |
| bambuterol | -1.821 | 4 | vorinostat | 1.682 | 12 |
| pentoxifylline | -1.823 | 5 | thioproperazine | 1.653 | 5 |
| myricetin | -1.823 | 4 | N-acetyl-L-leucine | 1.652 | 4 |
| difenidol | -1.829 | 3 | LM-1685 | 1.641 | 3 |
| clonidine | -1.836 | 4 | antazoline | 1.636 | 4 |
| podophyllotoxin | -1.836 | 4 | tretinoin | 1.623 | 22 |
| sulindac | -1.842 | 7 | epiandrosterone | 1.617 | 4 |
| indoprofen | -1.843 | 4 | letrozole | 1.600 | 4 |
| Prestwick-857 | -1.853 | 4 | isoxsuprine | 1.599 | 5 |
| alfuzosin | -1.853 | 5 | levobunolol | 1.590 | 4 |
| metolazone | -1.857 | 5 | 4-hydroxyphenazone | 1.589 | 5 |
| nitrofurantoin | -1.864 | 5 | perhexiline | 1.586 | 4 |
| molsidomine | -1.864 | 4 | HC toxin | 1.585 | 1 |
| iocetamic acid | -1.872 | 4 | theophylline | 1.580 | 4 |
| xylometazoline | -1.872 | 4 | etidronic acid | 1.577 | 4 |
| fenoterol | -1.873 | 3 | tropine | 1.566 | 4 |
| etamsylate | -1.873 | 4 | cycloserine | 1.561 | 4 |
| dimethadione | -1.874 | 4 | nipecotic acid | 1.559 | 4 |
| phentolamine | -1.880 | 7 | pentetic acid | 1.554 | 5 |
| testosterone | -1.882 | 5 | prednisolone | 1.552 | 5 |
| lasalocid | -1.887 | 4 | streptozocin | 1.546 | 4 |
| mephenesin | -1.889 | 5 | arachidonic acid | 1.525 | 3 |
| quinidine | -1.893 | 3 | amiodarone | 1.524 | 5 |
| prednisone | -1.897 | 5 | bendroflumethiazide | 1.521 | 6 |
| piracetam | -1.901 | 4 | tanespimycin | 1.493 | 62 |
| betamethasone | -1.903 | 3 | proxyphylline | 1.484 | 4 |
| L-methionine sulfoximine | -1.909 | 4 | tetracycline | 1.478 | 5 |
| tetracycline | -1.920 | 5 | sisomicin | 1.474 | 4 |
| trimethoprim | -1.923 | 5 | clenbuterol | 1.473 | 5 |
| pancuronium bromide | -1.925 | 4 | ioversol | 1.469 | 4 |
| diperodon | -1.927 | 3 | neomycin | 1.466 | 5 |
| reserpine | -1.929 | 3 | felbinac | 1.461 | 4 |
| propofol | -1.930 | 4 | mefloquine | 1.450 | 5 |
| fludroxycortide | -1.931 | 5 | valinomycin | 1.443 | 4 |
| naproxen | -1.934 | 9 | ricinine | 1.440 | 4 |
| sulfathiazole | -1.943 | 5 | demecarium bromide | 1.434 | 4 |
| dextromethorphan | -1.949 | 4 | clebopride | 1.433 | 4 |
| ceforanide | -1.956 | 4 | mepacrine | 1.423 | 2 |
| rolitetracycline | -1.971 | 4 | piracetam | 1.420 | 4 |
| famotidine | -1.977 | 5 | diphemanil metilsulfate | 1.412 | 5 |
| ciclosporin | -1.978 | 6 | epirizole | 1.407 | 5 |
| propafenone | -1.979 | 4 | 6-bromoindirubin-3-oxime | 1.404 | 7 |
| dihydrostreptomycin | -1.980 | 5 | pyridoxine | 1.395 | 4 |
| mercaptopurine | -1.983 | 2 | emetine | 1.355 | 4 |
| galantamine | -1.985 | 4 | cefmetazole | 1.354 | 4 |
| trichlormethiazide | -1.990 | 4 | naproxen | 1.341 | 9 |
| nicotinic acid | -1.992 | 4 | clorgiline | 1.340 | 4 |
| cetirizine | -2.008 | 4 | 0179445-0000 | 1.339 | 8 |
| buspirone | -2.016 | 4 | etodolac | 1.327 | 5 |
| Prestwick-674 | -2.020 | 6 | mephenesin | 1.324 | 5 |
| memantine | -2.023 | 4 | cinoxacin | 1.319 | 4 |
| flavoxate | -2.023 | 4 | bromocriptine | 1.317 | 5 |
| propylthiouracil | -2.026 | 4 | pyrithyldione | 1.309 | 4 |
| bromopride | -2.027 | 6 | tiratricol | 1.309 | 4 |
| Prestwick-1080 | -2.030 | 4 | ethoxyquin | 1.294 | 5 |
| levamisole | -2.034 | 4 | bethanechol | 1.288 | 4 |
| sulfaphenazole | -2.034 | 4 | noscapine | 1.286 | 4 |
| anisomycin | -2.043 | 4 | furosemide | 1.271 | 4 |
| procaine | -2.045 | 5 | 0316684-0000 | 1.269 | 4 |
| pipenzolate bromide | -2.045 | 4 | ajmaline | 1.268 | 3 |
| sulconazole | -2.046 | 4 | vitexin | 1.262 | 4 |
| betaxolol | -2.047 | 4 | chlormezanone | 1.257 | 4 |
| 1,4-chrysenequinone | -2.047 | 2 | scopolamine | 1.255 | 4 |
| convolamine | -2.054 | 4 | droperidol | 1.237 | 4 |
| digitoxigenin | -2.054 | 4 | latamoxef | 1.231 | 3 |
| meclofenoxate | -2.064 | 6 | piperacetazine | 1.228 | 4 |
| diazoxide | -2.064 | 5 | pivmecillinam | 1.222 | 4 |
| epiandrosterone | -2.067 | 4 | AR-A014418 | 1.209 | 3 |
| etofylline | -2.067 | 5 | dosulepin | 1.198 | 4 |
| carteolol | -2.069 | 4 | fluspirilene | 1.193 | 4 |
| orphenadrine | -2.069 | 6 | vigabatrin | 1.166 | 3 |
| probucol | -2.073 | 6 | gibberellic acid | 1.145 | 4 |
| dimenhydrinate | -2.074 | 4 | finasteride | 1.139 | 6 |
| dequalinium chloride | -2.075 | 4 | sulfaphenazole | 1.132 | 4 |
| scopolamine N-oxide | -2.078 | 5 | bisacodyl | 1.131 | 4 |
| midodrine | -2.079 | 5 | benzethonium chloride | 1.130 | 3 |
| MK-886 | -2.085 | 2 | articaine | 1.129 | 3 |
| loracarbef | -2.086 | 4 | altretamine | 1.107 | 4 |
| diflunisal | -2.089 | 5 | fluticasone | 1.101 | 4 |
| sulfacetamide | -2.092 | 4 | isocarboxazid | 1.099 | 5 |
| viomycin | -2.094 | 4 | cetirizine | 1.092 | 4 |
| roxithromycin | -2.095 | 4 | pheniramine | 1.080 | 5 |
| rolipram | -2.098 | 4 | nortriptyline | 1.012 | 4 |
| meclocycline | -2.099 | 4 | amiloride | 1.007 | 5 |
| iopromide | -2.100 | 4 | oligomycin | 1.000 | 1 |
| milrinone | -2.104 | 3 | proguanil | 1.000 | 3 |
| penbutolol | -2.106 | 3 | carcinine | 0.997 | 4 |
| STOCK1N-28457 | -2.112 | 3 | acenocoumarol | 0.997 | 5 |
| sulfadiazine | -2.115 | 5 | metanephrine | 0.990 | 5 |
| aztreonam | -2.116 | 5 | chloramphenicol | 0.985 | 4 |
| ritodrine | -2.120 | 4 | oxybutynin | 0.985 | 4 |
| Prestwick-864 | -2.120 | 4 | moroxydine | 0.966 | 5 |
| pargyline | -2.122 | 4 | sotalol | 0.962 | 4 |
| aconitine | -2.128 | 4 | triamterene | 0.962 | 5 |
| tolbutamide | -2.128 | 7 | H-7 | 0.953 | 4 |
| ribostamycin | -2.129 | 4 | zidovudine | 0.945 | 4 |
| nicardipine | -2.130 | 4 | cyclic adenosine monophosphate | 0.942 | 4 |
| (-)-isoprenaline | -2.132 | 4 | tridihexethyl | 0.936 | 4 |
| levothyroxine sodium | -2.133 | 4 | propantheline bromide | 0.933 | 4 |
| ionomycin | -2.142 | 3 | trifluoperazine | 0.929 | 16 |
| hydrastinine | -2.144 | 5 | cantharidin | 0.924 | 1 |
| chlorpromazine | -2.148 | 19 | proxymetacaine | 0.923 | 4 |
| altretamine | -2.148 | 4 | guanethidine | 0.919 | 3 |
| colecalciferol | -2.151 | 4 | Prestwick-972 | 0.919 | 3 |
| betulinic acid | -2.162 | 4 | sulfadiazine | 0.914 | 5 |
| tetracaine | -2.163 | 3 | clozapine | 0.912 | 17 |
| bendroflumethiazide | -2.168 | 6 | naftidrofuryl | 0.911 | 4 |
| solasodine | -2.171 | 6 | trichostatin A | 0.891 | 182 |
| trifluoperazine | -2.173 | 16 | Prestwick-642 | 0.888 | 4 |
| thioperamide | -2.173 | 5 | dinoprost | 0.882 | 4 |
| acemetacin | -2.182 | 4 | isosorbide | 0.862 | 4 |
| protoveratrine A | -2.183 | 4 | ronidazole | 0.861 | 3 |
| scopoletin | -2.189 | 2 | kawain | 0.857 | 5 |
| brompheniramine | -2.191 | 4 | co-dergocrine mesilate | 0.856 | 4 |
| 6-benzylaminopurine | -2.201 | 5 | 7-aminocephalosporanic acid | 0.850 | 4 |
| lymecycline | -2.207 | 4 | scopolamine N-oxide | 0.845 | 5 |
| carbenoxolone | -2.209 | 4 | tiapride | 0.845 | 5 |
| BAS-012416453 | -2.213 | 3 | methanthelinium bromide | 0.843 | 4 |
| fluorometholone | -2.216 | 4 | ethionamide | 0.839 | 3 |
| monobenzone | -2.218 | 4 | nifurtimox | 0.833 | 4 |
| napelline | -2.219 | 4 | erythromycin | 0.830 | 5 |
| hexamethonium bromide | -2.220 | 5 | desipramine | 0.826 | 4 |
| dobutamine | -2.223 | 4 | flufenamic acid | 0.821 | 6 |
| ergocalciferol | -2.227 | 4 | gemfibrozil | 0.820 | 5 |
| progesterone | -2.240 | 4 | remoxipride | 0.819 | 4 |
| riboflavin | -2.247 | 4 | bucladesine | 0.811 | 6 |
| sulfaguanidine | -2.264 | 5 | orphenadrine | 0.794 | 6 |
| amitriptyline | -2.265 | 6 | benzocaine | 0.780 | 4 |
| staurosporine | -2.271 | 2 | sulfadoxine | 0.777 | 3 |
| guaifenesin | -2.276 | 6 | sulfamethoxazole | 0.765 | 5 |
| felodipine | -2.277 | 7 | dexverapamil | 0.765 | 1 |
| epirizole | -2.278 | 5 | mifepristone | 0.760 | 4 |
| thalidomide | -2.280 | 7 | STOCK1N-28457 | 0.758 | 3 |
| thioridazine | -2.282 | 20 | nalidixic acid | 0.750 | 5 |
| rofecoxib | -2.287 | 6 | ramifenazone | 0.750 | 4 |
| canrenoic acid | -2.291 | 4 | buflomedil | 0.748 | 4 |
| desoxycortone | -2.295 | 4 | sulfamerazine | 0.735 | 5 |
| xamoterol | -2.298 | 3 | L-methionine sulfoximine | 0.718 | 4 |
| benzathine benzylpenicillin | -2.309 | 4 | mianserin | 0.718 | 5 |
| alfadolone | -2.317 | 3 | hydroxyachillin | 0.716 | 4 |
| cefmetazole | -2.319 | 4 | caffeic acid | 0.716 | 3 |
| vidarabine | -2.326 | 4 | foliosidine | 0.694 | 6 |
| atractyloside | -2.329 | 5 | memantine | 0.689 | 4 |
| metanephrine | -2.329 | 5 | BCB000038 | 0.680 | 4 |
| prilocaine | -2.338 | 6 | LY-294002 | 0.680 | 61 |
| pyrazinamide | -2.344 | 4 | ethisterone | 0.663 | 6 |
| dacarbazine | -2.349 | 4 | meteneprost | 0.653 | 4 |
| adipiodone | -2.349 | 4 | 2-aminobenzenesulfonamide | 0.652 | 4 |
| mestranol | -2.353 | 4 | proglumide | 0.649 | 5 |
| baclofen | -2.355 | 5 | mycophenolic acid | 0.642 | 3 |
| tacrine | -2.363 | 4 | trazodone | 0.641 | 3 |
| trichostatin A | -2.364 | 182 | doxorubicin | 0.625 | 3 |
| cotinine | -2.368 | 6 | skimmianine | 0.622 | 4 |
| chenodeoxycholic acid | -2.368 | 4 | probucol | 0.617 | 6 |
| carbinoxamine | -2.369 | 4 | hymecromone | 0.613 | 4 |
| thiostrepton | -2.378 | 4 | oxybuprocaine | 0.611 | 4 |
| phenacetin | -2.382 | 4 | iopanoic acid | 0.609 | 4 |
| isocarboxazid | -2.386 | 5 | hycanthone | 0.603 | 4 |
| skimmianine | -2.389 | 4 | biotin | 0.584 | 3 |
| biotin | -2.399 | 3 | hydralazine | 0.584 | 6 |
| clioquinol | -2.405 | 5 | CP-690334-01 | 0.577 | 8 |
| isoflupredone | -2.412 | 3 | dl-alpha tocopherol | 0.575 | 4 |
| rescinnamine | -2.412 | 3 | bambuterol | 0.574 | 4 |
| ebselen | -2.417 | 3 | naphazoline | 0.558 | 5 |
| proguanil | -2.419 | 3 | isoniazid | 0.555 | 5 |
| lovastatin | -2.430 | 4 | clotrimazole | 0.547 | 5 |
| paracetamol | -2.430 | 4 | tetrandrine | 0.546 | 4 |
| dinoprostone | -2.431 | 4 | fenbendazole | 0.537 | 4 |
| cinchocaine | -2.431 | 5 | fluoxetine | 0.536 | 4 |
| zuclopenthixol | -2.436 | 4 | selegiline | 0.512 | 4 |
| Prestwick-972 | -2.438 | 3 | N-acetyl-L-aspartic acid | 0.496 | 4 |
| piperine | -2.458 | 4 | bromperidol | 0.486 | 3 |
| W-13 | -2.463 | 2 | levothyroxine sodium | 0.484 | 4 |
| meticrane | -2.464 | 5 | cefapirin | 0.478 | 4 |
| hydrochlorothiazide | -2.467 | 5 | bephenium hydroxynaphthoate | 0.465 | 5 |
| thioproperazine | -2.468 | 5 | difenidol | 0.464 | 3 |
| N6-methyladenosine | -2.470 | 4 | mefexamide | 0.458 | 4 |
| solanine | -2.477 | 4 | atovaquone | 0.457 | 3 |
| Prestwick-860 | -2.477 | 4 | cefsulodin | 0.444 | 4 |
| erythromycin | -2.496 | 5 | canavanine | 0.429 | 3 |
| nifenazone | -2.498 | 5 | fluorometholone | 0.424 | 4 |
| sulfaquinoxaline | -2.498 | 3 | SC-19220 | 0.417 | 4 |
| thioguanosine | -2.503 | 4 | flunisolide | 0.402 | 6 |
| nadide | -2.504 | 4 | felodipine | 0.393 | 7 |
| estriol | -2.505 | 4 | trichlormethiazide | 0.390 | 4 |
| coralyne | -2.508 | 4 | terguride | 0.384 | 8 |
| strophanthidin | -2.517 | 4 | khellin | 0.378 | 5 |
| droperidol | -2.524 | 4 | metoprolol | 0.358 | 4 |
| Prestwick-984 | -2.524 | 4 | minoxidil | 0.358 | 5 |
| nadolol | -2.526 | 4 | pepstatin | 0.350 | 4 |
| pridinol | -2.530 | 4 | carteolol | 0.349 | 4 |
| tranylcypromine | -2.531 | 5 | trifluridine | 0.348 | 4 |
| primidone | -2.531 | 4 | bufexamac | 0.342 | 4 |
| citiolone | -2.536 | 6 | novobiocin | 0.334 | 9 |
| sisomicin | -2.537 | 4 | telenzepine | 0.333 | 4 |
| loxapine | -2.538 | 4 | moxonidine | 0.329 | 3 |
| 3-acetylcoumarin | -2.541 | 5 | midecamycin | 0.325 | 5 |
| albendazole | -2.548 | 3 | isoetarine | 0.277 | 4 |
| ascorbic acid | -2.552 | 4 | tetryzoline | 0.271 | 3 |
| nifurtimox | -2.553 | 4 | alfuzosin | 0.267 | 5 |
| N-acetyl-L-aspartic acid | -2.556 | 4 | valproic acid | 0.264 | 57 |
| sertaconazole | -2.560 | 4 | aztreonam | 0.264 | 5 |
| 2-aminobenzenesulfonamide | -2.566 | 4 | GW-8510 | 0.256 | 4 |
| dantrolene | -2.567 | 6 | diflorasone | 0.241 | 4 |
| kinetin | -2.571 | 4 | azacyclonol | 0.226 | 5 |
| tacrolimus | -2.572 | 3 | desoxycortone | 0.226 | 4 |
| pimethixene | -2.573 | 3 | karakoline | 0.218 | 6 |
| 8-azaguanine | -2.577 | 4 | arecoline | 0.206 | 4 |
| cefotetan | -2.579 | 3 | ciclacillin | 0.203 | 4 |
| phenindione | -2.580 | 4 | maprotiline | 0.202 | 4 |
| fluphenazine | -2.581 | 18 | thioperamide | 0.198 | 5 |
| acetohexamide | -2.589 | 4 | pancuronium bromide | 0.198 | 4 |
| fluorocurarine | -2.592 | 4 | repaglinide | 0.188 | 4 |
| zardaverine | -2.597 | 4 | eucatropine | 0.186 | 6 |
| picrotoxinin | -2.601 | 4 | monobenzone | 0.185 | 4 |
| ivermectin | -2.605 | 5 | Prestwick-685 | 0.185 | 5 |
| glafenine | -2.606 | 4 | N-acetylmuramic acid | 0.171 | 4 |
| bufexamac | -2.608 | 4 | zomepirac | 0.159 | 4 |
| tiaprofenic acid | -2.621 | 4 | vincamine | 0.156 | 6 |
| chlorprothixene | -2.623 | 4 | tetroquinone | 0.154 | 4 |
| tetryzoline | -2.635 | 3 | etofenamate | 0.153 | 4 |
| tinidazole | -2.636 | 6 | disopyramide | 0.150 | 4 |
| carbarsone | -2.637 | 4 | aminophylline | 0.144 | 4 |
| ethotoin | -2.639 | 6 | nilutamide | 0.144 | 4 |
| hydralazine | -2.646 | 6 | amikacin | 0.137 | 4 |
| isosorbide | -2.649 | 4 | tiletamine | 0.129 | 4 |
| piperacetazine | -2.661 | 4 | tenoxicam | 0.123 | 4 |
| mebhydrolin | -2.663 | 4 | dexibuprofen | 0.121 | 4 |
| metergoline | -2.667 | 4 | xylometazoline | 0.109 | 4 |
| equilin | -2.670 | 5 | isopropamide iodide | 0.101 | 4 |
| SB-203580 | -2.672 | 5 | dinoprostone | 0.098 | 4 |
| ethionamide | -2.685 | 3 | oxamic acid | 0.085 | 1 |
| nizatidine | -2.688 | 4 | nicotinic acid | 0.081 | 4 |
| eucatropine | -2.691 | 6 | Prestwick-860 | 0.079 | 4 |
| epivincamine | -2.698 | 4 | solanine | 0.078 | 4 |
| flupentixol | -2.700 | 4 | mepyramine | 0.072 | 4 |
| suprofen | -2.703 | 4 | enoxacin | 0.063 | 4 |
| enalapril | -2.704 | 4 | myricetin | 0.061 | 4 |
| serotonin | -2.715 | 5 | aminoglutethimide | 0.019 | 3 |
| delsoline | -2.722 | 4 | bezafibrate | 0.016 | 4 |
| etifenin | -2.727 | 4 | isoxicam | 0.010 | 5 |
| sulfamethoxypyridazine | -2.740 | 5 | nialamide | -0.002 | 4 |
| omeprazole | -2.753 | 4 | indapamide | -0.004 | 6 |
| moxonidine | -2.758 | 3 | cortisone | -0.005 | 3 |
| aminophenazone | -2.758 | 5 | mebhydrolin | -0.007 | 4 |
| naringin | -2.762 | 4 | Prestwick-664 | -0.019 | 6 |
| etamivan | -2.765 | 4 | tiabendazole | -0.044 | 4 |
| theophylline | -2.768 | 4 | gefitinib | -0.053 | 1 |
| fluticasone | -2.776 | 4 | oxamniquine | -0.059 | 4 |
| hecogenin | -2.797 | 4 | ciclopirox | -0.062 | 4 |
| dosulepin | -2.818 | 4 | hydrochlorothiazide | -0.073 | 5 |
| tolnaftate | -2.826 | 5 | benzthiazide | -0.074 | 4 |
| amiloride | -2.829 | 5 | glycopyrronium bromide | -0.083 | 5 |
| iproniazid | -2.830 | 5 | mestranol | -0.088 | 4 |
| doxazosin | -2.843 | 4 | carbamazepine | -0.093 | 8 |
| niridazole | -2.843 | 4 | Prestwick-689 | -0.097 | 4 |
| sulfamethizole | -2.848 | 4 | atropine oxide | -0.107 | 5 |
| theobromine | -2.858 | 4 | carbachol | -0.109 | 4 |
| (-)-MK-801 | -2.864 | 4 | chlorpromazine | -0.110 | 19 |
| deptropine | -2.866 | 4 | isocorydine | -0.123 | 4 |
| phensuximide | -2.868 | 4 | danazol | -0.126 | 4 |
| tomatidine | -2.894 | 4 | papaverine | -0.128 | 4 |
| bephenium hydroxynaphthoate | -2.898 | 5 | sulpiride | -0.151 | 5 |
| cortisone | -2.899 | 3 | isotretinoin | -0.167 | 4 |
| methazolamide | -2.909 | 4 | reserpine | -0.178 | 3 |
| ginkgolide A | -2.919 | 4 | hemicholinium | -0.186 | 4 |
| picotamide | -2.931 | 5 | zaprinast | -0.191 | 4 |
| nitrendipine | -2.931 | 5 | protoveratrine A | -0.195 | 4 |
| cefotaxime | -2.936 | 5 | salsolinol | -0.207 | 3 |
| dexibuprofen | -2.938 | 4 | SC-58125 | -0.214 | 4 |
| telenzepine | -2.942 | 4 | dopamine | -0.217 | 1 |
| co-dergocrine mesilate | -2.950 | 4 | sulfamonomethoxine | -0.218 | 4 |
| raloxifene | -2.955 | 7 | pirlindole | -0.235 | 3 |
| lactobionic acid | -2.957 | 4 | florfenicol | -0.244 | 4 |
| gabexate | -2.959 | 4 | thiethylperazine | -0.260 | 4 |
| 7-aminocephalosporanic acid | -2.961 | 4 | benzydamine | -0.261 | 4 |
| oxybenzone | -2.967 | 4 | pramocaine | -0.281 | 5 |
| cefalotin | -2.980 | 4 | cefadroxil | -0.286 | 4 |
| diflorasone | -2.983 | 4 | (+/-)-catechin | -0.289 | 4 |
| Prestwick-685 | -2.983 | 5 | famprofazone | -0.303 | 6 |
| felbinac | -2.986 | 4 | procarbazine | -0.307 | 3 |
| cyproterone | -2.991 | 4 | ozagrel | -0.315 | 4 |
| caffeic acid | -2.991 | 3 | celecoxib | -0.321 | 5 |
| dicycloverine | -2.996 | 5 | amitriptyline | -0.321 | 6 |
| timolol | -2.999 | 4 | merbromin | -0.324 | 5 |
| tropine | -3.008 | 4 | alclometasone | -0.330 | 4 |
| miconazole | -3.010 | 5 | cyclobenzaprine | -0.347 | 4 |
| clindamycin | -3.021 | 5 | pipemidic acid | -0.348 | 3 |
| mephentermine | -3.022 | 5 | cefaclor | -0.349 | 4 |
| thiamazole | -3.028 | 6 | altizide | -0.349 | 4 |
| practolol | -3.029 | 4 | trimethadione | -0.355 | 4 |
| mepyramine | -3.030 | 4 | pseudopelletierine | -0.362 | 4 |
| diphemanil metilsulfate | -3.031 | 5 | metoclopramide | -0.362 | 6 |
| sulfadimidine | -3.031 | 6 | mevalolactone | -0.369 | 3 |
| tiapride | -3.032 | 5 | napelline | -0.383 | 4 |
| beta-escin | -3.042 | 6 | orciprenaline | -0.400 | 4 |
| ioxaglic acid | -3.054 | 3 | crotamiton | -0.413 | 4 |
| metacycline | -3.055 | 4 | hexestrol | -0.413 | 4 |
| velnacrine | -3.056 | 4 | quinisocaine | -0.430 | 4 |
| levopropoxyphene | -3.061 | 4 | calcium folinate | -0.437 | 5 |
| finasteride | -3.068 | 6 | oleandomycin | -0.443 | 5 |
| isometheptene | -3.089 | 4 | tolbutamide | -0.452 | 7 |
| thiethylperazine | -3.095 | 4 | lithocholic acid | -0.454 | 6 |
| methyldopate | -3.097 | 4 | iproniazid | -0.459 | 5 |
| meropenem | -3.112 | 4 | flavoxate | -0.470 | 4 |
| gelsemine | -3.113 | 4 | Prestwick-665 | -0.470 | 5 |
| ouabain | -3.114 | 4 | ceforanide | -0.480 | 4 |
| tremorine | -3.116 | 4 | CAY-10397 | -0.492 | 3 |
| atropine | -3.131 | 4 | octopamine | -0.516 | 4 |
| furazolidone | -3.152 | 4 | metolazone | -0.531 | 5 |
| cefazolin | -3.160 | 5 | metixene | -0.543 | 4 |
| pizotifen | -3.160 | 4 | carbinoxamine | -0.571 | 4 |
| flunisolide | -3.182 | 6 | topiramate | -0.573 | 1 |
| flunarizine | -3.182 | 4 | bemegride | -0.578 | 4 |
| mebendazole | -3.186 | 5 | paromomycin | -0.586 | 4 |
| nabumetone | -3.190 | 4 | profenamine | -0.590 | 4 |
| terazosin | -3.197 | 4 | methazolamide | -0.598 | 4 |
| clemizole | -3.203 | 5 | miconazole | -0.599 | 5 |
| letrozole | -3.207 | 4 | salbutamol | -0.605 | 5 |
| LY-294002 | -3.213 | 61 | asiaticoside | -0.615 | 4 |
| altizide | -3.218 | 4 | halcinonide | -0.617 | 5 |
| dinoprost | -3.224 | 4 | prazosin | -0.621 | 6 |
| terfenadine | -3.224 | 3 | alverine | -0.649 | 4 |
| dilazep | -3.233 | 5 | sulfamethizole | -0.654 | 4 |
| tyloxapol | -3.235 | 4 | benfotiamine | -0.655 | 5 |
| vitexin | -3.236 | 4 | lovastatin | -0.659 | 4 |
| clebopride | -3.237 | 4 | Chicago Sky Blue 6B | -0.679 | 4 |
| gemfibrozil | -3.241 | 5 | succinylsulfathiazole | -0.696 | 4 |
| moracizine | -3.247 | 4 | rilmenidine | -0.699 | 4 |
| colistin | -3.265 | 4 | naftopidil | -0.712 | 3 |
| amprolium | -3.275 | 5 | 1,5-isoquinolinediol | -0.723 | 1 |
| bumetanide | -3.279 | 4 | Prestwick-1085 | -0.735 | 4 |
| nipecotic acid | -3.284 | 4 | pizotifen | -0.745 | 4 |
| (+/-)-catechin | -3.289 | 4 | sulfabenzamide | -0.805 | 4 |
| urapidil | -3.291 | 4 | canadine | -0.822 | 4 |
| propoxycaine | -3.297 | 4 | rimexolone | -0.824 | 4 |
| cinchonidine | -3.299 | 4 | niflumic acid | -0.827 | 4 |
| promazine | -3.312 | 6 | prasterone | -0.829 | 4 |
| benperidol | -3.317 | 4 | naltrexone | -0.833 | 5 |
| SC-19220 | -3.329 | 4 | dilazep | -0.838 | 5 |
| homochlorcyclizine | -3.335 | 4 | lisinopril | -0.843 | 3 |
| valinomycin | -3.336 | 4 | ethotoin | -0.855 | 6 |
| rilmenidine | -3.337 | 4 | glafenine | -0.856 | 4 |
| mifepristone | -3.338 | 4 | cefalotin | -0.876 | 4 |
| yohimbic acid | -3.344 | 3 | fenoprofen | -0.883 | 6 |
| methoxamine | -3.345 | 4 | butacaine | -0.884 | 4 |
| apramycin | -3.355 | 4 | prochlorperazine | -0.913 | 16 |
| tenoxicam | -3.358 | 4 | disulfiram | -0.918 | 5 |
| pempidine | -3.369 | 5 | guanfacine | -0.922 | 5 |
| propranolol | -3.374 | 4 | mefenamic acid | -0.925 | 5 |
| artemisinin | -3.376 | 3 | 3-aminobenzamide | -0.935 | 1 |
| bromperidol | -3.378 | 3 | ifenprodil | -0.937 | 4 |
| flumetasone | -3.391 | 6 | picrotoxinin | -0.960 | 4 |
| ifenprodil | -3.400 | 4 | hexetidine | -0.967 | 4 |
| cefepime | -3.400 | 4 | phenacetin | -0.989 | 4 |
| butacaine | -3.401 | 4 | 11-deoxy-16,16-dimethylprostaglandin E2 | -1.010 | 4 |
| chloramphenicol | -3.416 | 4 | trioxysalen | -1.011 | 4 |
| benzonatate | -3.420 | 5 | tobramycin | -1.013 | 4 |
| levonorgestrel | -3.428 | 6 | abamectin | -1.018 | 4 |
| S-propranolol | -3.439 | 4 | glimepiride | -1.021 | 4 |
| diclofenamide | -3.446 | 4 | apramycin | -1.035 | 4 |
| phthalylsulfathiazole | -3.449 | 5 | palmatine | -1.049 | 4 |
| salsolinol | -3.451 | 3 | yohimbic acid | -1.069 | 3 |
| luteolin | -3.464 | 4 | diazoxide | -1.081 | 5 |
| meprylcaine | -3.471 | 4 | betulin | -1.110 | 3 |
| octopamine | -3.473 | 4 | pirenperone | -1.114 | 5 |
| decamethonium bromide | -3.477 | 4 | eticlopride | -1.128 | 4 |
| fenofibrate | -3.482 | 3 | vancomycin | -1.129 | 4 |
| estropipate | -3.489 | 4 | chloropyrazine | -1.142 | 4 |
| tobramycin | -3.505 | 4 | zimeldine | -1.146 | 5 |
| alimemazine | -3.509 | 4 | nafcillin | -1.159 | 4 |
| pilocarpine | -3.511 | 4 | heliotrine | -1.160 | 6 |
| quinisocaine | -3.513 | 4 | spectinomycin | -1.168 | 4 |
| gabapentin | -3.514 | 4 | urapidil | -1.186 | 4 |
| pirenperone | -3.528 | 5 | azaperone | -1.198 | 4 |
| cobalt chloride | -3.535 | 3 | troglitazone | -1.202 | 16 |
| buflomedil | -3.541 | 4 | lomefloxacin | -1.221 | 6 |
| paromomycin | -3.556 | 4 | liothyronine | -1.233 | 4 |
| doxorubicin | -3.556 | 3 | helveticoside | -1.235 | 6 |
| guanethidine | -3.557 | 3 | doxylamine | -1.250 | 5 |
| guanfacine | -3.559 | 5 | estradiol | -1.272 | 15 |
| acenocoumarol | -3.560 | 5 | idazoxan | -1.274 | 4 |
| morantel | -3.567 | 5 | piperlongumine | -1.277 | 2 |
| hexylcaine | -3.575 | 4 | monorden | -1.285 | 22 |
| lobeline | -3.586 | 4 | pyrazinamide | -1.294 | 4 |
| chloropyrazine | -3.595 | 4 | vinpocetine | -1.298 | 4 |
| oxyphenbutazone | -3.599 | 4 | phenazopyridine | -1.340 | 4 |
| foliosidine | -3.603 | 6 | acetohexamide | -1.345 | 4 |
| roxarsone | -3.613 | 4 | acemetacin | -1.348 | 4 |
| 3-hydroxy-DL-kynurenine | -3.625 | 6 | hydrocotarnine | -1.356 | 4 |
| benzamil | -3.631 | 6 | meclocycline | -1.370 | 4 |
| trimethobenzamide | -3.636 | 5 | drofenine | -1.373 | 4 |
| norcyclobenzaprine | -3.643 | 4 | ondansetron | -1.394 | 4 |
| ricinine | -3.644 | 4 | conessine | -1.395 | 4 |
| iopanoic acid | -3.646 | 4 | bromopride | -1.401 | 6 |
| perhexiline | -3.651 | 4 | riluzole | -1.416 | 5 |
| nefopam | -3.658 | 5 | ipratropium bromide | -1.416 | 3 |
| metamizole sodium | -3.677 | 6 | F0447-0125 | -1.417 | 4 |
| benzethonium chloride | -3.677 | 3 | sulfadimidine | -1.423 | 6 |
| spiramycin | -3.687 | 6 | iloprost | -1.437 | 3 |
| 3-acetamidocoumarin | -3.688 | 4 | delsoline | -1.443 | 4 |
| isoxicam | -3.689 | 5 | rifampicin | -1.454 | 4 |
| amiodarone | -3.729 | 5 | etynodiol | -1.472 | 4 |
| rifampicin | -3.729 | 4 | oxprenolol | -1.476 | 4 |
| etanidazole | -3.739 | 4 | mebendazole | -1.479 | 5 |
| ozagrel | -3.743 | 4 | deptropine | -1.503 | 4 |
| drofenine | -3.758 | 4 | propoxycaine | -1.519 | 4 |
| tocainide | -3.769 | 4 | nifuroxazide | -1.522 | 4 |
| dapsone | -3.769 | 5 | benperidol | -1.523 | 4 |
| prasterone | -3.770 | 4 | rofecoxib | -1.540 | 6 |
| nilutamide | -3.778 | 4 | flunarizine | -1.543 | 4 |
| demeclocycline | -3.779 | 6 | butyl hydroxybenzoate | -1.551 | 5 |
| sitosterol | -3.809 | 4 | glycocholic acid | -1.565 | 4 |
| amiprilose | -3.830 | 4 | mephenytoin | -1.579 | 4 |
| idoxuridine | -3.830 | 5 | phenylpropanolamine | -1.585 | 4 |
| selegiline | -3.855 | 4 | terfenadine | -1.592 | 3 |
| monensin | -3.856 | 6 | tomatidine | -1.597 | 4 |
| articaine | -3.873 | 3 | zuclopenthixol | -1.606 | 4 |
| ursodeoxycholic acid | -3.877 | 3 | 2-deoxy-D-glucose | -1.607 | 1 |
| levocabastine | -3.891 | 4 | tolmetin | -1.610 | 4 |
| piretanide | -3.891 | 4 | meclozine | -1.613 | 5 |
| trimipramine | -3.894 | 4 | promethazine | -1.614 | 4 |
| etofenamate | -3.896 | 4 | ribavirin | -1.619 | 4 |
| diethylstilbestrol | -3.910 | 6 | piroxicam | -1.628 | 4 |
| hydroflumethiazide | -3.932 | 5 | phenelzine | -1.633 | 6 |
| cinchonine | -3.934 | 4 | leflunomide | -1.646 | 4 |
| clenbuterol | -3.935 | 5 | ramipril | -1.662 | 4 |
| nortriptyline | -3.935 | 4 | clomifene | -1.681 | 4 |
| chlorcyclizine | -3.941 | 6 | sulfadimethoxine | -1.694 | 5 |
| noscapine | -3.949 | 4 | omeprazole | -1.723 | 4 |
| sulpiride | -3.971 | 5 | sulfanilamide | -1.728 | 4 |
| oxantel | -3.977 | 4 | menadione | -1.752 | 2 |
| hexetidine | -3.978 | 4 | diethylstilbestrol | -1.764 | 6 |
| vigabatrin | -3.981 | 3 | triflupromazine | -1.768 | 4 |
| pentetic acid | -3.987 | 5 | nicardipine | -1.769 | 4 |
| phenazopyridine | -3.992 | 4 | tacrolimus | -1.774 | 3 |
| remoxipride | -4.001 | 4 | homatropine | -1.783 | 5 |
| ciclopirox | -4.010 | 4 | methacholine chloride | -1.784 | 3 |
| ofloxacin | -4.023 | 5 | propofol | -1.785 | 4 |
| epitiostanol | -4.030 | 4 | imipramine | -1.788 | 4 |
| dicloxacillin | -4.034 | 4 | idoxuridine | -1.794 | 5 |
| oxybuprocaine | -4.037 | 4 | flumetasone | -1.808 | 6 |
| clidinium bromide | -4.040 | 4 | bicuculline | -1.811 | 4 |
| cefuroxime | -4.041 | 4 | epivincamine | -1.819 | 4 |
| profenamine | -4.057 | 4 | dehydrocholic acid | -1.821 | 5 |
| ethambutol | -4.077 | 5 | 3-acetamidocoumarin | -1.826 | 4 |
| harmaline | -4.081 | 4 | Prestwick-1084 | -1.826 | 4 |
| dipivefrine | -4.093 | 4 | buspirone | -1.833 | 4 |
| piperlongumine | -4.098 | 2 | retrorsine | -1.866 | 4 |
| alclometasone | -4.104 | 4 | domperidone | -1.872 | 4 |
| vancomycin | -4.128 | 4 | mexiletine | -1.890 | 6 |
| N-acetylmuramic acid | -4.130 | 4 | levocabastine | -1.895 | 4 |
| cefaclor | -4.140 | 4 | tioguanine | -1.897 | 1 |
| bepridil | -4.141 | 4 | nadide | -1.900 | 4 |
| N-acetyl-L-leucine | -4.143 | 4 | metformin | -1.916 | 10 |
| ondansetron | -4.146 | 4 | fluvastatin | -1.947 | 4 |
| abamectin | -4.148 | 4 | biperiden | -1.954 | 5 |
| ethisterone | -4.174 | 6 | brompheniramine | -1.966 | 4 |
| pentamidine | -4.185 | 5 | ofloxacin | -1.967 | 5 |
| etynodiol | -4.197 | 4 | neostigmine bromide | -1.980 | 4 |
| terguride | -4.204 | 8 | sulmazole | -1.984 | 3 |
| diethylcarbamazine | -4.211 | 4 | levodopa | -1.991 | 5 |
| butamben | -4.216 | 4 | todralazine | -1.998 | 5 |
| asiaticoside | -4.219 | 4 | calcium pantothenate | -1.999 | 4 |
| cefalonium | -4.231 | 3 | propidium iodide | -2.004 | 4 |
| trioxysalen | -4.235 | 4 | ethambutol | -2.004 | 5 |
| tolmetin | -4.240 | 4 | clemizole | -2.032 | 5 |
| nifuroxazide | -4.243 | 4 | etiocholanolone | -2.056 | 6 |
| megestrol | -4.267 | 4 | clidinium bromide | -2.058 | 4 |
| pronetalol | -4.273 | 4 | anisomycin | -2.062 | 4 |
| mefenamic acid | -4.281 | 5 | chlorogenic acid | -2.072 | 4 |
| probenecid | -4.294 | 4 | iopamidol | -2.085 | 4 |
| orciprenaline | -4.317 | 4 | mesalazine | -2.099 | 5 |
| levodopa | -4.319 | 5 | kinetin | -2.132 | 4 |
| praziquantel | -4.328 | 4 | cyclopentolate | -2.141 | 4 |
| Prestwick-983 | -4.330 | 3 | fluorocurarine | -2.152 | 4 |
| fluvastatin | -4.348 | 4 | sitosterol | -2.160 | 4 |
| mevalolactone | -4.353 | 3 | chloropyramine | -2.188 | 4 |
| leflunomide | -4.358 | 4 | enilconazole | -2.190 | 4 |
| mesalazine | -4.364 | 5 | tacrine | -2.201 | 4 |
| pramocaine | -4.370 | 5 | saquinavir | -2.214 | 4 |
| isoniazid | -4.374 | 5 | dydrogesterone | -2.216 | 4 |
| etiocholanolone | -4.385 | 6 | clindamycin | -2.217 | 5 |
| harpagoside | -4.397 | 4 | pronetalol | -2.230 | 4 |
| nafcillin | -4.398 | 4 | gliquidone | -2.237 | 4 |
| terconazole | -4.403 | 4 | sirolimus | -2.265 | 44 |
| metoprolol | -4.412 | 4 | etamivan | -2.265 | 4 |
| fluspirilene | -4.422 | 4 | eldeline | -2.266 | 6 |
| pivmecillinam | -4.435 | 4 | 0198306-0000 | -2.272 | 4 |
| phenelzine | -4.435 | 6 | alpha-ergocryptine | -2.287 | 6 |
| benfotiamine | -4.444 | 5 | ketoprofen | -2.312 | 6 |
| niflumic acid | -4.449 | 4 | harman | -2.325 | 4 |
| indapamide | -4.478 | 6 | ursodeoxycholic acid | -2.326 | 3 |
| azaperone | -4.488 | 4 | pilocarpine | -2.327 | 4 |
| boldine | -4.509 | 4 | (-)-atenolol | -2.332 | 4 |
| neomycin | -4.514 | 5 | dicoumarol | -2.346 | 6 |
| betulin | -4.517 | 3 | phthalylsulfathiazole | -2.349 | 5 |
| propantheline bromide | -4.531 | 4 | S-propranolol | -2.388 | 4 |
| tiletamine | -4.541 | 4 | norethisterone | -2.393 | 4 |
| Prestwick-967 | -4.547 | 4 | sulfacetamide | -2.408 | 4 |
| biperiden | -4.579 | 5 | isometheptene | -2.441 | 4 |
| gliquidone | -4.586 | 4 | meptazinol | -2.441 | 4 |
| flurbiprofen | -4.620 | 5 | ellipticine | -2.455 | 4 |
| repaglinide | -4.621 | 4 | cobalt chloride | -2.475 | 3 |
| zomepirac | -4.623 | 4 | R-atenolol | -2.481 | 4 |
| cyclic adenosine monophosphate | -4.625 | 4 | serotonin | -2.481 | 5 |
| disopyramide | -4.642 | 4 | monensin | -2.485 | 6 |
| dropropizine | -4.650 | 4 | diphenylpyraline | -2.487 | 6 |
| glibenclamide | -4.653 | 4 | dexpanthenol | -2.496 | 4 |
| prazosin | -4.657 | 6 | nefopam | -2.503 | 5 |
| calcium pantothenate | -4.661 | 4 | methyldopate | -2.511 | 4 |
| dexpropranolol | -4.677 | 3 | cefoxitin | -2.556 | 4 |
| mephenytoin | -4.682 | 4 | ethaverine | -2.578 | 4 |
| calcium folinate | -4.692 | 5 | alsterpaullone | -2.592 | 3 |
| Prestwick-1085 | -4.696 | 4 | alprenolol | -2.599 | 4 |
| azapropazone | -4.697 | 3 | Prestwick-857 | -2.627 | 4 |
| guanadrel | -4.698 | 5 | thioguanosine | -2.643 | 4 |
| ethoxyquin | -4.700 | 5 | nifenazone | -2.662 | 5 |
| bupivacaine | -4.706 | 4 | diloxanide | -2.683 | 4 |
| dicoumarol | -4.735 | 6 | bupivacaine | -2.696 | 4 |
| diloxanide | -4.744 | 4 | dimenhydrinate | -2.724 | 4 |
| benfluorex | -4.757 | 4 | enalapril | -2.773 | 4 |
| ramipril | -4.760 | 4 | hexylcaine | -2.780 | 4 |
| xylazine | -4.761 | 4 | BCB000039 | -2.793 | 3 |
| (-)-atenolol | -4.762 | 4 | moxisylyte | -2.811 | 5 |
| hymecromone | -4.763 | 4 | dipivefrine | -2.819 | 4 |
| florfenicol | -4.775 | 4 | benzylpenicillin | -2.829 | 4 |
| isoetarine | -4.780 | 4 | nitrofural | -2.849 | 4 |
| benzocaine | -4.791 | 4 | tetramisole | -2.869 | 4 |
| minocycline | -4.824 | 5 | nadolol | -2.914 | 4 |
| cloperastine | -4.824 | 6 | rolitetracycline | -2.917 | 4 |
| canavanine | -4.826 | 3 | pindolol | -2.922 | 5 |
| carbachol | -4.833 | 4 | promazine | -2.941 | 6 |
| chloropyramine | -4.838 | 4 | loxapine | -2.968 | 4 |
| etidronic acid | -4.845 | 4 | dyclonine | -2.970 | 4 |
| helveticoside | -4.848 | 6 | cefixime | -2.981 | 4 |
| securinine | -4.864 | 4 | triprolidine | -3.026 | 4 |
| idazoxan | -4.872 | 4 | ketorolac | -3.027 | 4 |
| nordihydroguaiaretic acid | -4.876 | 15 | natamycin | -3.045 | 4 |
| metyrapone | -4.877 | 4 | allantoin | -3.052 | 5 |
| fursultiamine | -4.879 | 4 | mephentermine | -3.063 | 5 |
| mexiletine | -4.903 | 6 | 3-acetylcoumarin | -3.066 | 5 |
| bretylium tosilate | -4.934 | 4 | lactobionic acid | -3.069 | 4 |
| benzthiazide | -4.940 | 4 | norcyclobenzaprine | -3.083 | 4 |
| saquinavir | -4.941 | 4 | erastin | -3.094 | 4 |
| hydroxyachillin | -4.972 | 4 | etifenin | -3.102 | 4 |
| todralazine | -4.979 | 5 | decamethonium bromide | -3.110 | 4 |
| proxyphylline | -4.989 | 4 | probenecid | -3.182 | 4 |
| vinpocetine | -4.994 | 4 | cefotaxime | -3.216 | 5 |
| homatropine | -5.003 | 5 | metrizamide | -3.225 | 4 |
| liothyronine | -5.018 | 4 | fenbufen | -3.236 | 6 |
| tridihexethyl | -5.034 | 4 | dorzolamide | -3.255 | 4 |
| naftopidil | -5.036 | 3 | primidone | -3.369 | 4 |
| nalbuphine | -5.078 | 5 | tribenoside | -3.390 | 4 |
| isotretinoin | -5.096 | 4 | 6-benzylaminopurine | -3.399 | 5 |
| cyclobenzaprine | -5.104 | 4 | securinine | -3.422 | 4 |
| pralidoxime | -5.107 | 4 | chlortetracycline | -3.464 | 5 |
| ribavirin | -5.107 | 4 | dipyridamole | -3.498 | 6 |
| pepstatin | -5.114 | 4 | phensuximide | -3.517 | 4 |
| norethisterone | -5.132 | 4 | pipenzolate bromide | -3.550 | 4 |
| meclozine | -5.155 | 5 | gossypol | -3.580 | 6 |
| pregnenolone | -5.158 | 4 | piribedil | -3.599 | 4 |
| alsterpaullone | -5.184 | 3 | flucytosine | -3.601 | 4 |
| cefixime | -5.195 | 4 | piretanide | -3.615 | 4 |
| molindone | -5.200 | 4 | xylazine | -3.625 | 4 |
| 6-azathymine | -5.202 | 4 | procainamide | -3.638 | 4 |
| Prestwick-682 | -5.240 | 4 | hydroflumethiazide | -3.641 | 5 |
| ramifenazone | -5.244 | 4 | pentoxyverine | -3.675 | 4 |
| iohexol | -5.245 | 4 | bretylium tosilate | -3.691 | 4 |
| moxisylyte | -5.260 | 5 | rolipram | -3.735 | 4 |
| tiabendazole | -5.340 | 4 | oxedrine | -3.750 | 4 |
| benzylpenicillin | -5.389 | 4 | Prestwick-682 | -3.752 | 4 |
| iopamidol | -5.395 | 4 | galantamine | -3.762 | 4 |
| conessine | -5.399 | 4 | nordihydroguaiaretic acid | -3.767 | 15 |
| harman | -5.409 | 4 | nalbuphine | -3.767 | 5 |
| flumequine | -5.427 | 4 | molindone | -3.768 | 4 |
| aminophylline | -5.441 | 4 | 6-azathymine | -3.784 | 4 |
| Prestwick-642 | -5.508 | 4 | propranolol | -3.794 | 4 |
| pyridoxine | -5.511 | 4 | guanadrel | -3.820 | 5 |
| retrorsine | -5.548 | 4 | metrifonate | -3.868 | 5 |
| Prestwick-664 | -5.554 | 6 | dropropizine | -3.879 | 4 |
| trifluridine | -5.565 | 4 | moracizine | -3.882 | 4 |
| doxylamine | -5.570 | 5 | oxantel | -3.928 | 4 |
| allantoin | -5.580 | 5 | mesoridazine | -3.965 | 4 |
| succinylsulfathiazole | -5.585 | 4 | doxycycline | -3.971 | 5 |
| trihexyphenidyl | -5.594 | 3 | terconazole | -4.028 | 4 |
| pipemidic acid | -5.622 | 3 | gramine | -4.048 | 4 |
| tetrandrine | -5.630 | 4 | Prestwick-967 | -4.055 | 4 |
| palmatine | -5.652 | 4 | carbarsone | -4.077 | 4 |
| (+)-isoprenaline | -5.707 | 4 | cefalonium | -4.086 | 3 |
| Prestwick-692 | -5.747 | 4 | cefuroxime | -4.100 | 4 |
| clomifene | -5.806 | 4 | (-)-isoprenaline | -4.137 | 4 |
| dexpanthenol | -5.844 | 4 | metacycline | -4.143 | 4 |
| bicuculline | -5.858 | 4 | griseofulvin | -4.177 | 5 |
| doxycycline | -5.868 | 5 | naringin | -4.212 | 4 |
| neostigmine bromide | -5.904 | 4 | N-phenylanthranilic acid | -4.216 | 1 |
| enilconazole | -5.949 | 4 | amrinone | -4.254 | 4 |
| bisacodyl | -5.997 | 4 | flumequine | -4.266 | 4 |
| oxprenolol | -6.017 | 4 | fursultiamine | -4.284 | 4 |
| sulfadimethoxine | -6.036 | 5 | minocycline | -4.293 | 5 |
| piribedil | -6.050 | 4 | trihexyphenidyl | -4.306 | 3 |
| sulfanilamide | -6.103 | 4 | gelsemine | -4.330 | 4 |
| lithocholic acid | -6.129 | 6 | methoxamine | -4.333 | 4 |
| adrenosterone | -6.298 | 4 | harmaline | -4.394 | 4 |
| flucytosine | -6.370 | 4 | amiprilose | -4.394 | 4 |
| eldeline | -6.392 | 6 | cefotiam | -4.572 | 4 |
| chlorogenic acid | -6.433 | 4 | iocetamic acid | -4.573 | 4 |
| canadine | -6.451 | 4 | epitiostanol | -4.598 | 4 |
| dorzolamide | -6.458 | 4 | (+)-isoprenaline | -4.617 | 4 |
| tetramisole | -6.511 | 4 | adrenosterone | -4.619 | 4 |
| metrifonate | -6.511 | 5 | glibenclamide | -4.654 | 4 |
| tribenoside | -6.522 | 4 | mebeverine | -4.792 | 4 |
| bemegride | -6.530 | 4 | pergolide | -5.023 | 4 |
| sparteine | -6.556 | 4 | sparteine | -5.027 | 4 |
| ronidazole | -6.601 | 3 | arcaine | -5.085 | 4 |
| gramine | -6.615 | 4 | theobromine | -5.093 | 4 |
| metixene | -6.628 | 4 | prednisone | -5.096 | 5 |
| merbromin | -6.695 | 5 | meglumine | -5.149 | 4 |
| R-atenolol | -6.730 | 4 | trimethoprim | -5.302 | 5 |
| natamycin | -6.776 | 4 | diclofenamide | -5.578 | 4 |
| triprolidine | -6.824 | 4 | diethylcarbamazine | -5.672 | 4 |
| sulmazole | -6.967 | 3 | metyrapone | -5.841 | 4 |
| lisinopril | -7.060 | 3 | iohexol | -5.846 | 4 |
| meptazinol | -7.294 | 4 | etanidazole | -5.936 | 4 |
| meglumine | -7.365 | 4 | flecainide | -7.118 | 6 |
| butyl hydroxybenzoate | -7.434 | 5 | Prestwick-692 | -7.243 | 4 |
